# Supplementary material for: A Mettl16/m6A/mybl2b/Igf2bp1 axis ensures cell cycle progression of embryonic hematopoietic stem and progenitor cells
Source: EMBO J. 2024 Apr 11;43(10):1990–2014. doi: 10.1038/s44318-024-00082-9 (PMC11099167; doi:10.1038/s44318-024-00082-9)
Supplement: Supplementary file 1 — Appendix [file 44318_2024_82_MOESM1_ESM.pdf]

Table of contents

Appendix Figure S1 1

Appendix Figure S2 2

Appendix Figure S1

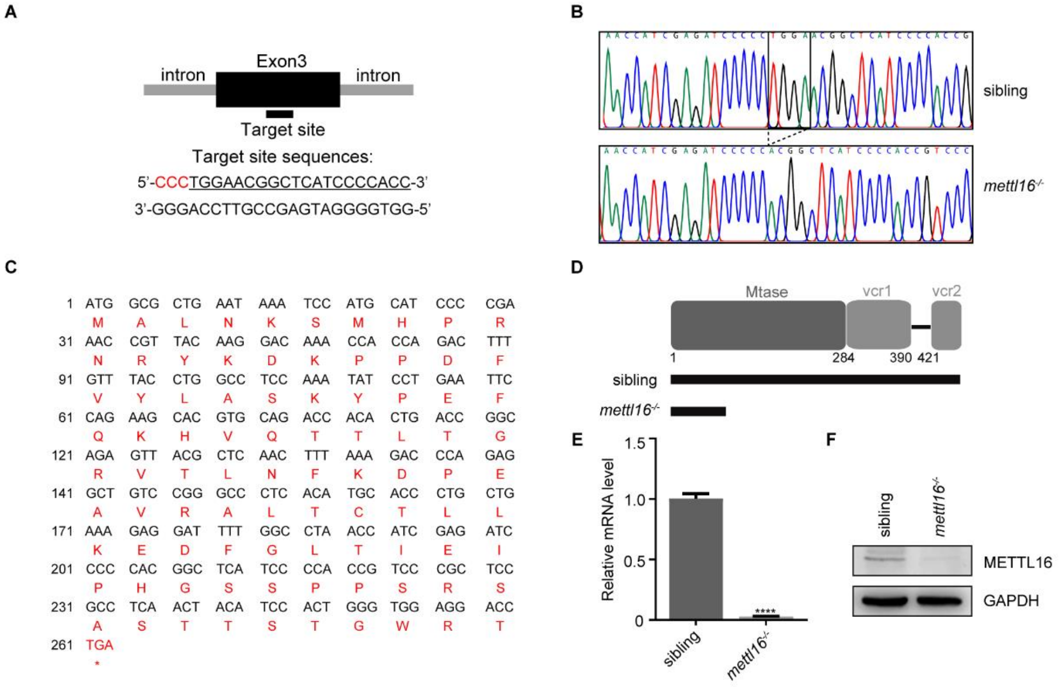

Appendix Figure S1. Generation of *mettl16* knockout zebrafish. (**A**) Diagram showing position of the target site and its sequence (underline) in zebrafish *mettl16* locus. PAM sequence (CCC) is shown in red. (**B**) Sanger sequencing results revealing a 4-bp genomic DNA fragment deletion from the target site in *mettl16* mutants. n≥ 10 per group, performed with 3 biological replicates. (**C** and **D**) Graphic representation of the truncated protein sequence in *mettl16* mutants. (**E** and **F**) The mRNA (E) and protein (F) level of *mettl16* in *mettl16* mutants at 3 dpf. n≥ 15 (E) or 20 (F) per group, performed with 3 biological replicates. Data are represented as mean ± SEM (E), **** adjusted *P*<0.0001, student's unpaired two-sided t-test (E).

Appendix Figure S2

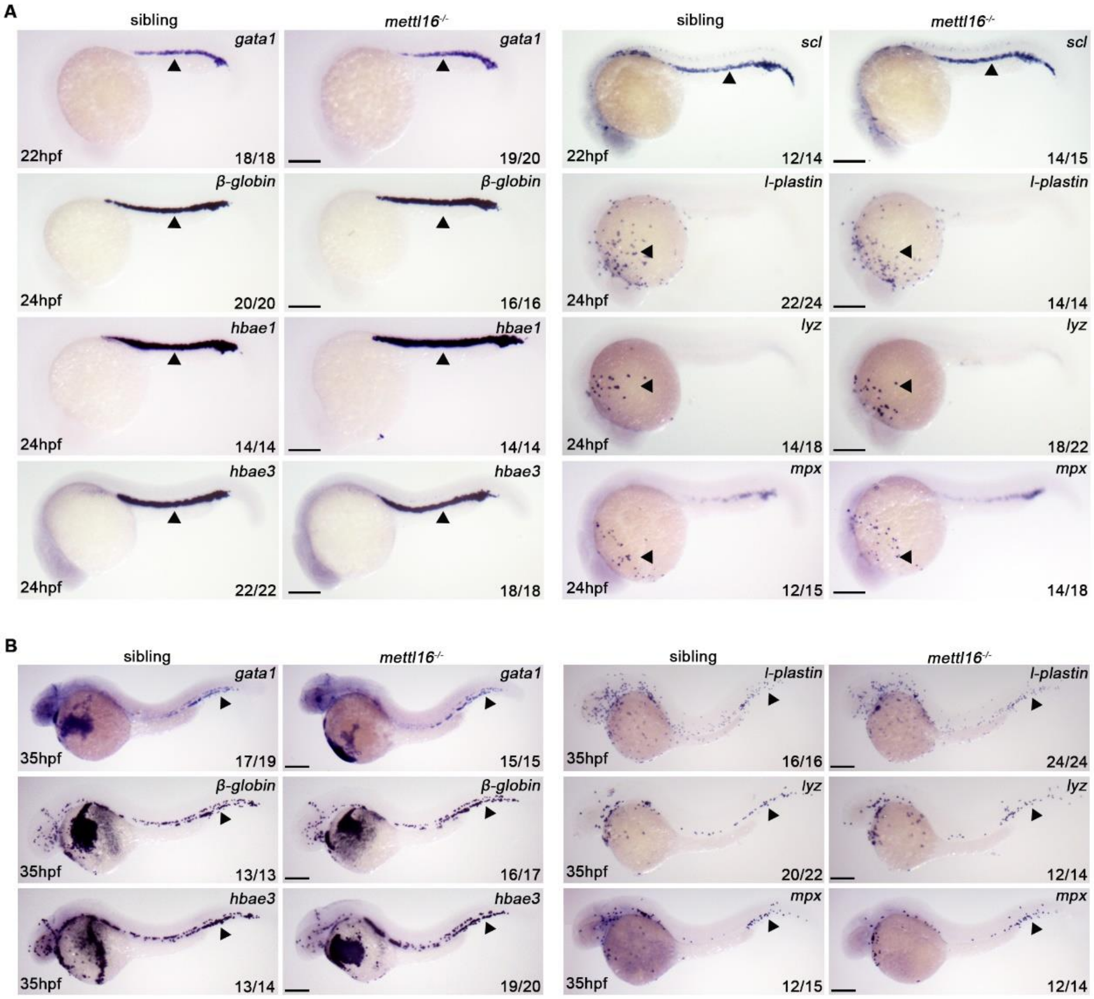

Appendix Figure S2. Primitive hematopoiesis and EMPs are undisturbed in *mettl16*$^{-/-}$ zebrafish. (**A**) Expression of differentiation markers for primitive hematopoiesis-*gata1* (erythroid), *b-globin* (erythroid), *hbae1* (erythroid), *hbae3* (erythroid), *scl* (hemangioblast), *l-plastin* (myeloid), *lyz* (myeloid) and *mpx* (myeloid) in sibling and *mettl16*$^{-/-}$ embryos at 24 hpf by WISH. Numbers at the bottom right indicate the number of embryos with similar staining pattern among all embryos examined. The black arrowheads indicate erythroid or myeloid cells. n= 3 dependent experiments. Scale bars, 50 μm. (**B**) WISH analysis showing the expression of differentiation markers for committed erythromyeloid progenitors-*gata1* (erythroid), *b-globin* (erythroid), *hbae3* (erythroid), *l-plastin* (myeloid), *lyz* (myeloid), and *mpx* (myeloid) in the posterior blood island (PBI) of siblings and *mettl16* mutants at 35 hpf. Numbers at the bottom right indicate the number of embryos with similar staining pattern among all embryos examined. The black arrowheads indicate erythroid or myeloid cells. n= 3 dependent experiments. Scale bar, 50 μm.
