# Supplementary figures and images for: A Mettl16/m6A/mybl2b/Igf2bp1 axis ensures cell cycle progression of embryonic hematopoietic stem and progenitor cells
Source: EMBO J. 2024 Apr 11;43(10):1990–2014. doi: 10.1038/s44318-024-00082-9 (PMC11099167; doi:10.1038/s44318-024-00082-9)

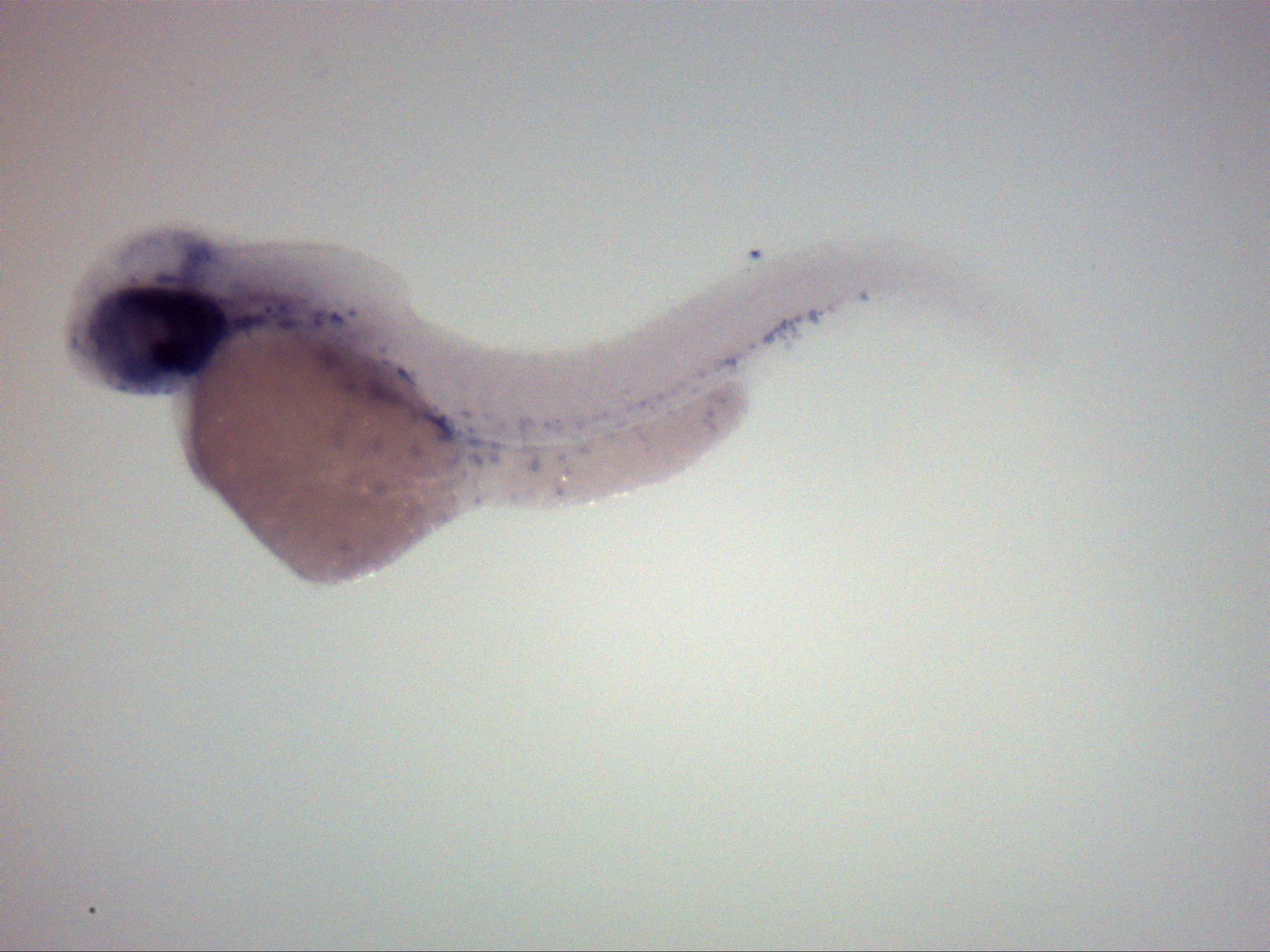

Supplement: Supplementary file 7 — Source data Fig. 2 [file 44318_2024_82_MOESM7_ESM.zip › Figure 2/2A-H/2A 2d cmyb sibling.tif]

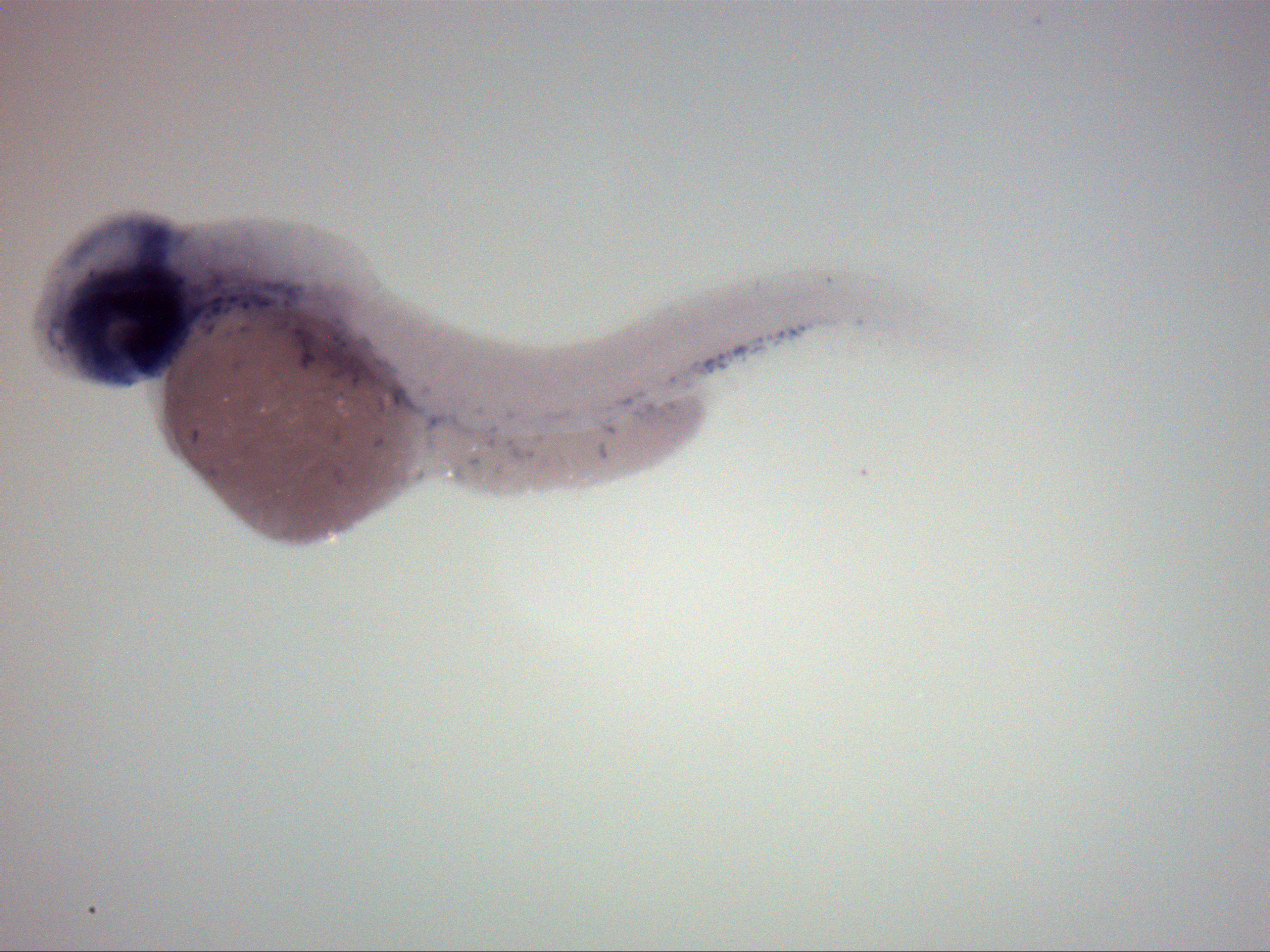

Supplement: Supplementary file 7 — Source data Fig. 2 [file 44318_2024_82_MOESM7_ESM.zip › Figure 2/2A-H/2B 2d cmyb mettl16--.tif]

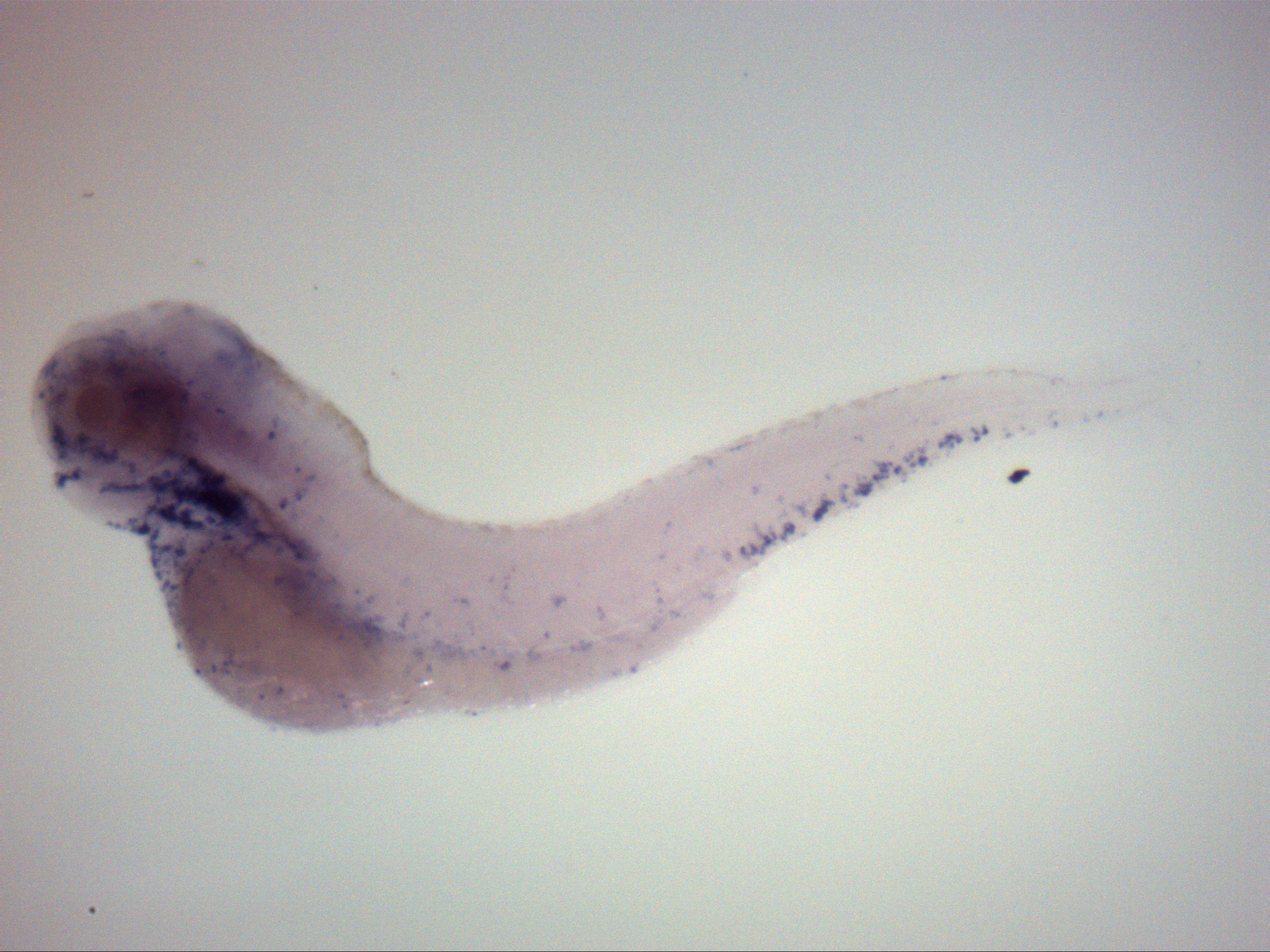

Supplement: Supplementary file 7 — Source data Fig. 2 [file 44318_2024_82_MOESM7_ESM.zip › Figure 2/2A-H/2C 3d cmyb sibling.tif]

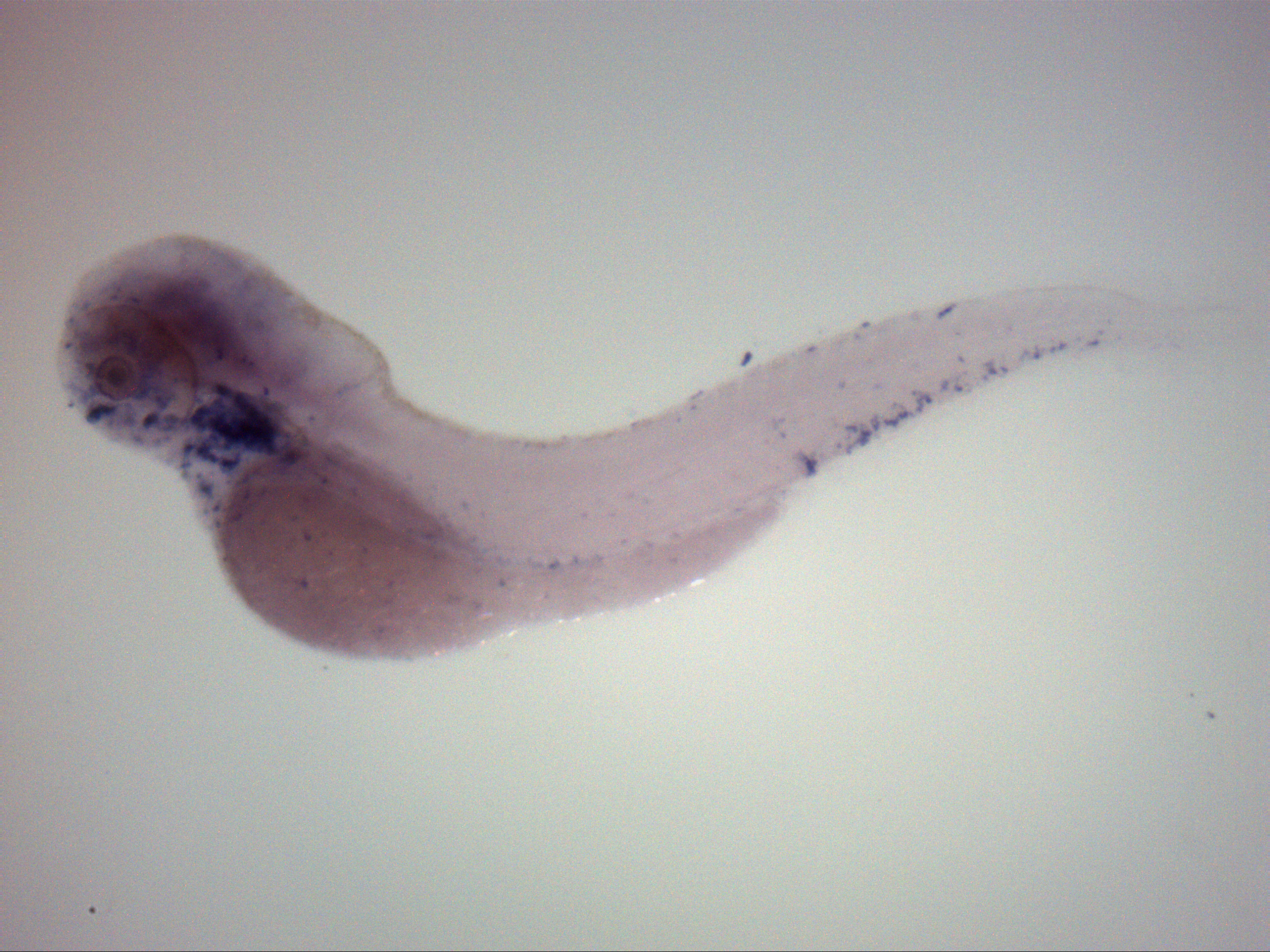

Supplement: Supplementary file 7 — Source data Fig. 2 [file 44318_2024_82_MOESM7_ESM.zip › Figure 2/2A-H/2D 3d cmyb mettl16--.tif]

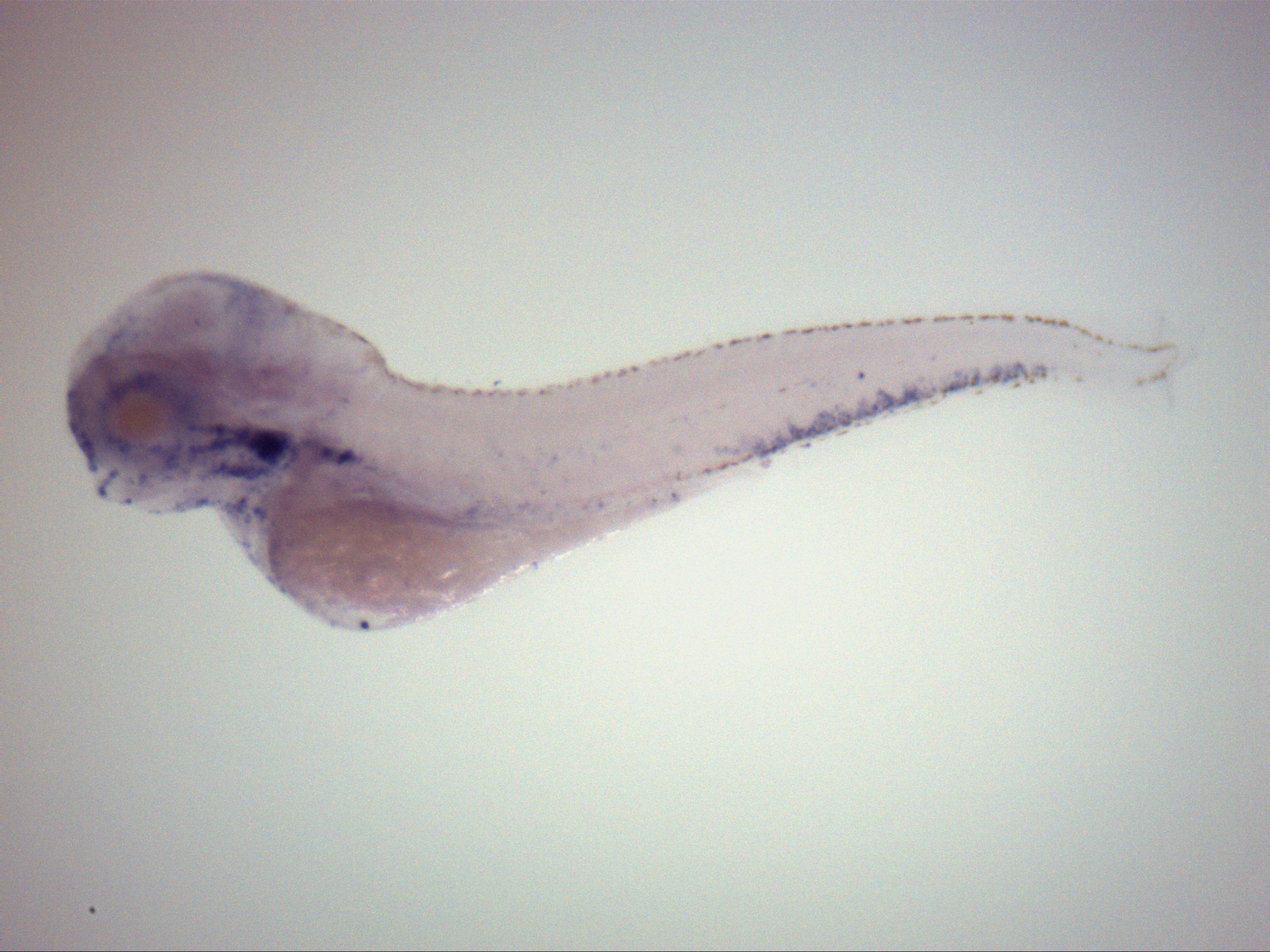

Supplement: Supplementary file 7 — Source data Fig. 2 [file 44318_2024_82_MOESM7_ESM.zip › Figure 2/2A-H/2E 4d cmyb sibling.tif]

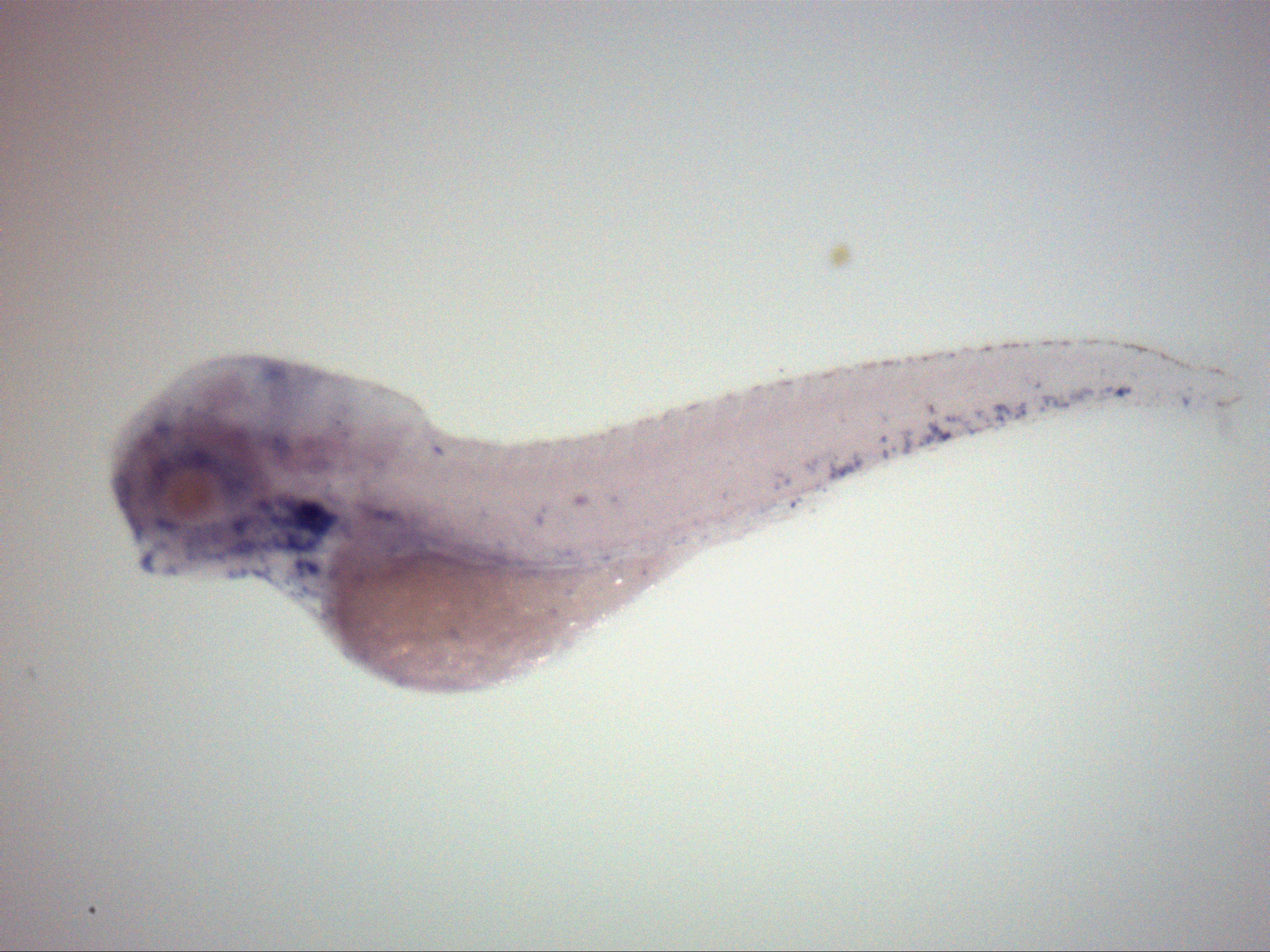

Supplement: Supplementary file 7 — Source data Fig. 2 [file 44318_2024_82_MOESM7_ESM.zip › Figure 2/2A-H/2F 4d cmyb mettl16--.tif]

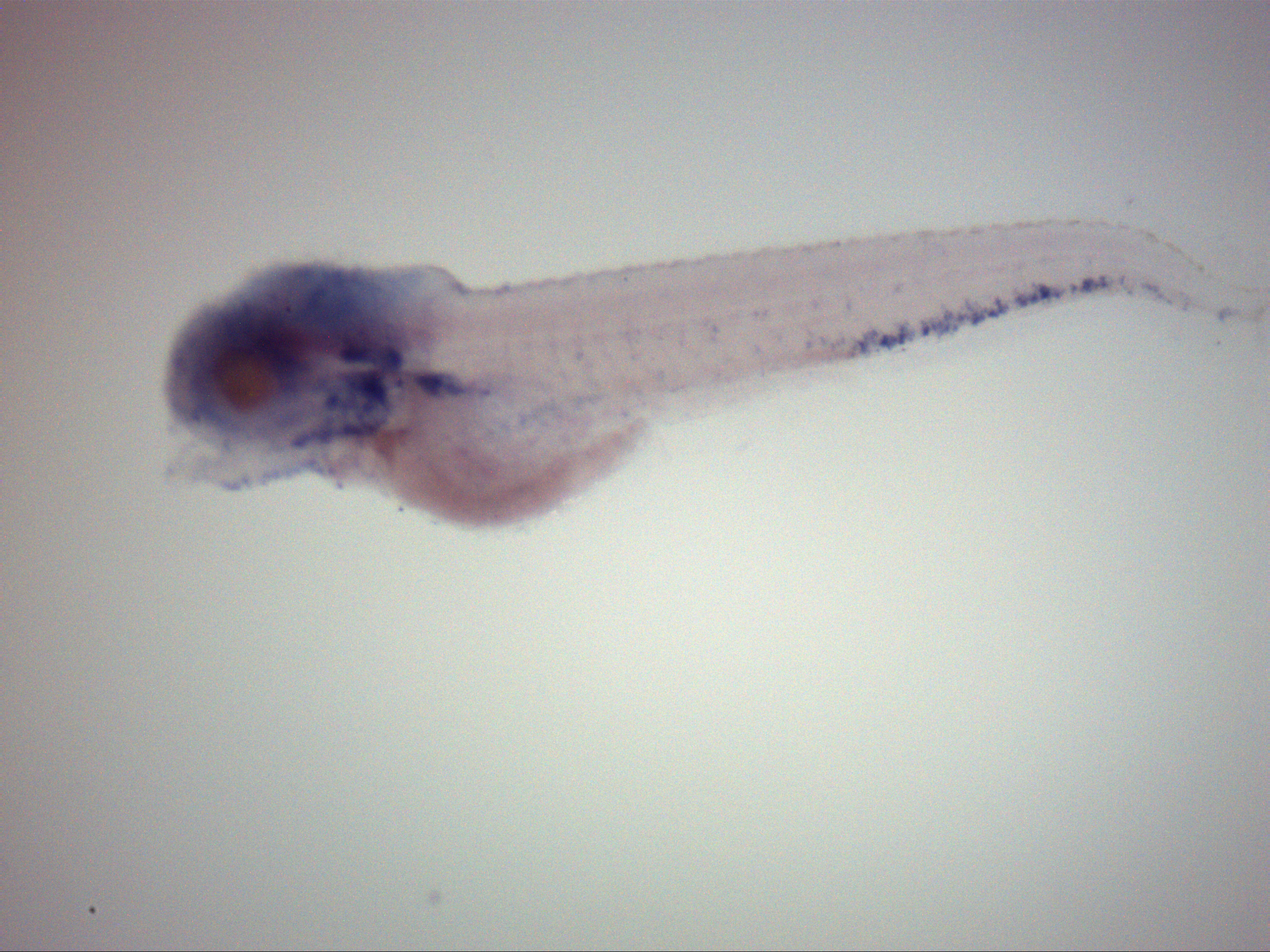

Supplement: Supplementary file 7 — Source data Fig. 2 [file 44318_2024_82_MOESM7_ESM.zip › Figure 2/2A-H/2G 5d cmyb sibling.tif]

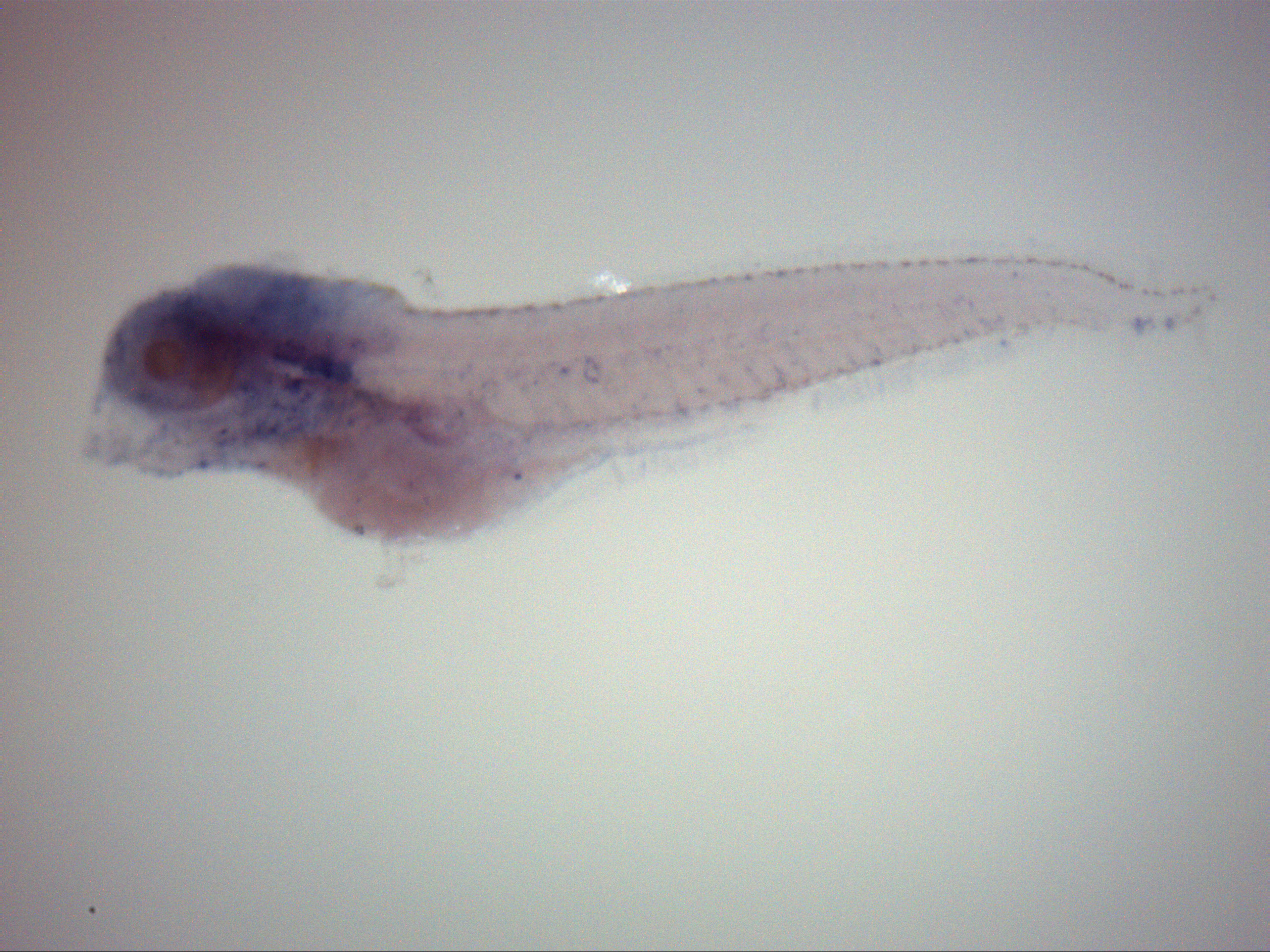

Supplement: Supplementary file 7 — Source data Fig. 2 [file 44318_2024_82_MOESM7_ESM.zip › Figure 2/2A-H/2H 5d cmyb mettl16--.tif]

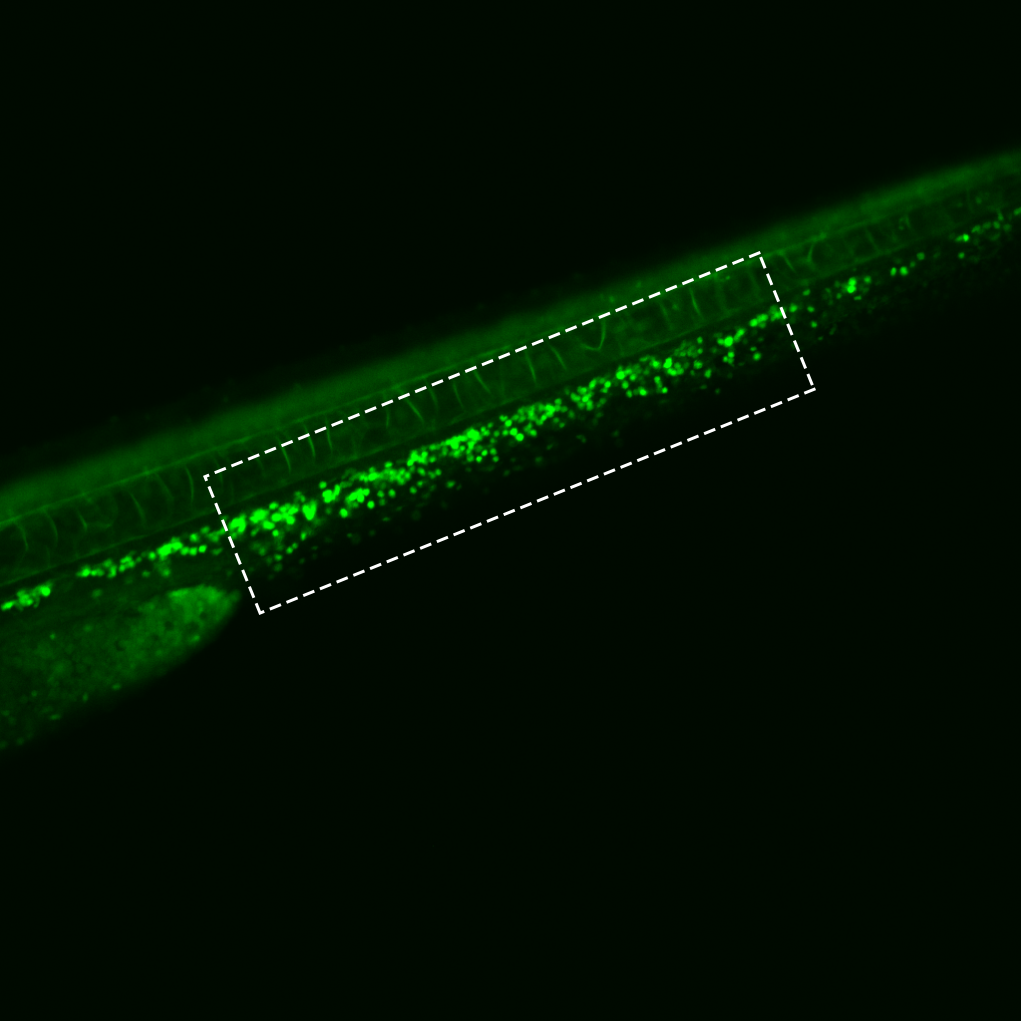

Supplement: Supplementary file 7 — Source data Fig. 2 [file 44318_2024_82_MOESM7_ESM.zip › Figure 2/2J/2dpf GFP mettl16--.tif]

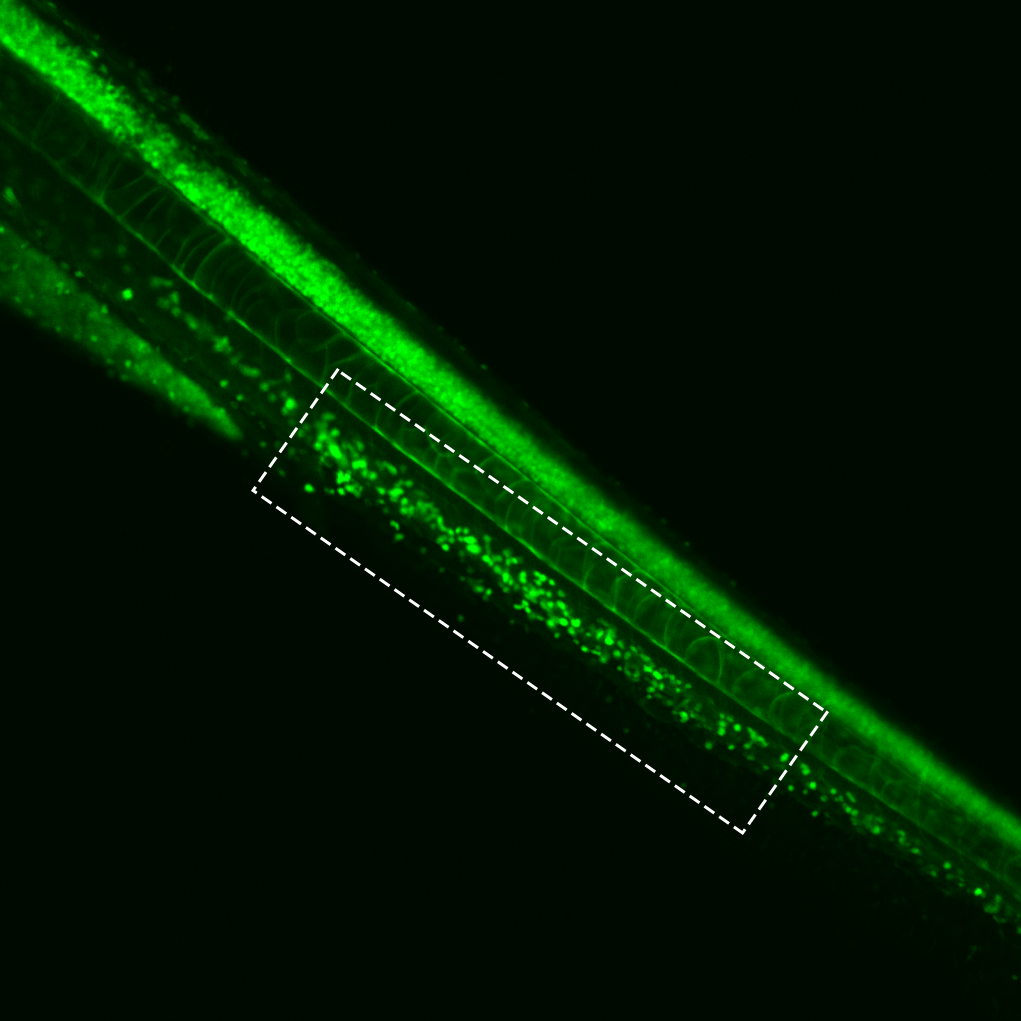

Supplement: Supplementary file 7 — Source data Fig. 2 [file 44318_2024_82_MOESM7_ESM.zip › Figure 2/2J/2dpf GFP sibling.tif]

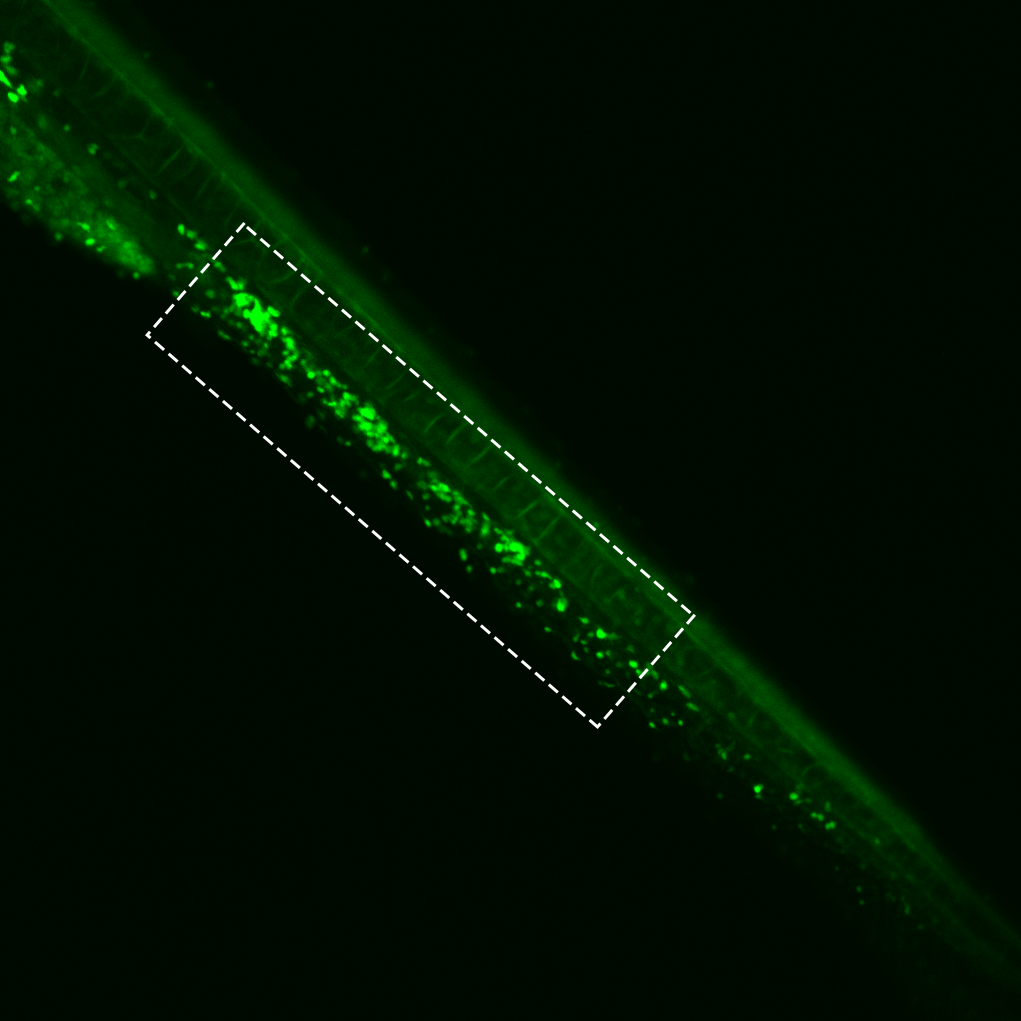

Supplement: Supplementary file 7 — Source data Fig. 2 [file 44318_2024_82_MOESM7_ESM.zip › Figure 2/2J/3dpf GFP mettl16--.tif]

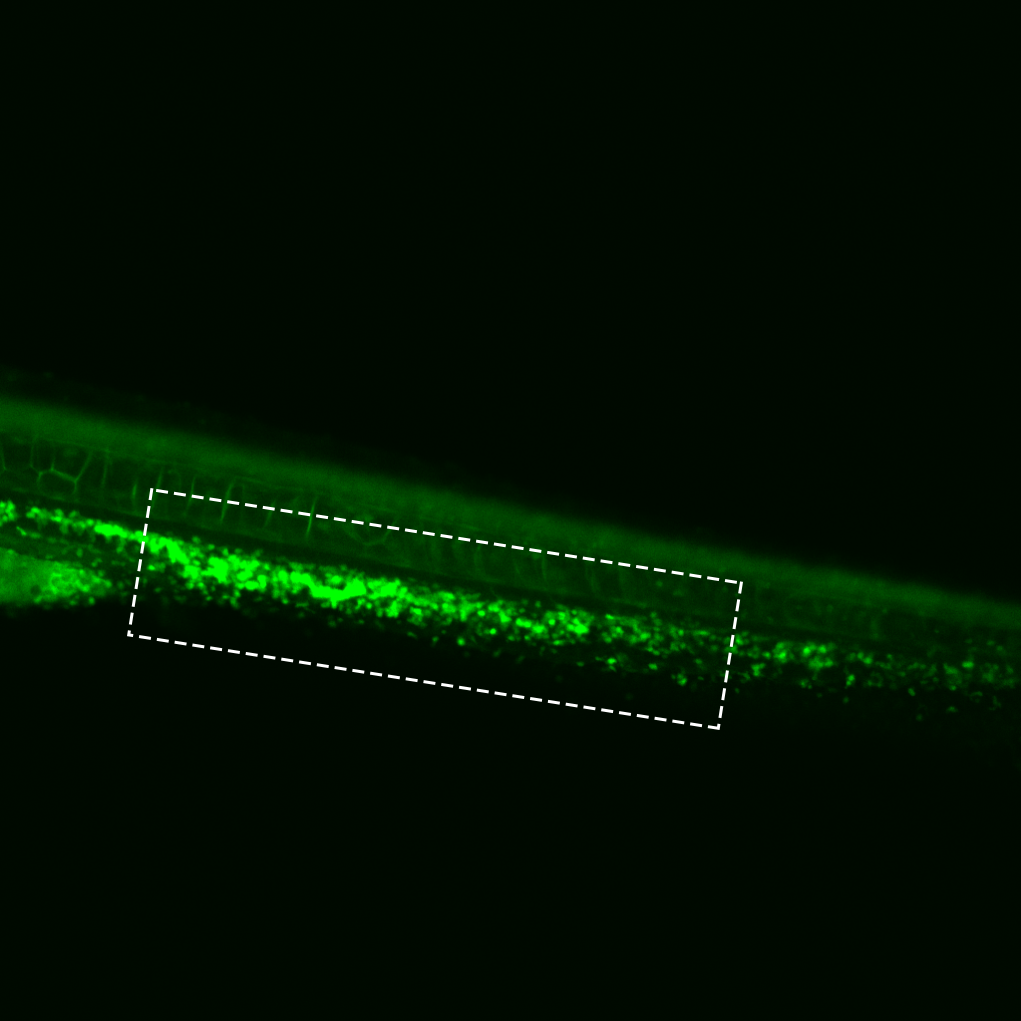

Supplement: Supplementary file 7 — Source data Fig. 2 [file 44318_2024_82_MOESM7_ESM.zip › Figure 2/2J/3dpf GFP sibling.tif]

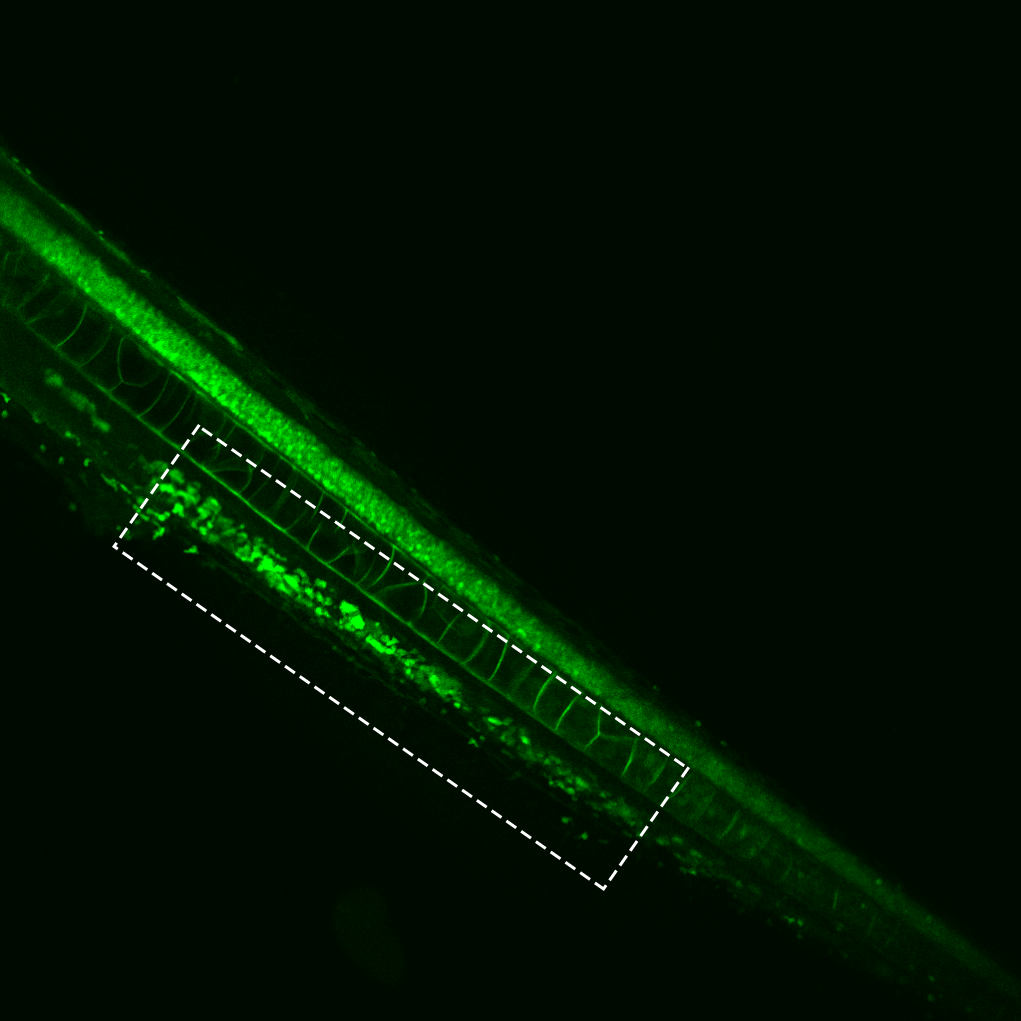

Supplement: Supplementary file 7 — Source data Fig. 2 [file 44318_2024_82_MOESM7_ESM.zip › Figure 2/2J/4dpf GFP mettl16--.tif]

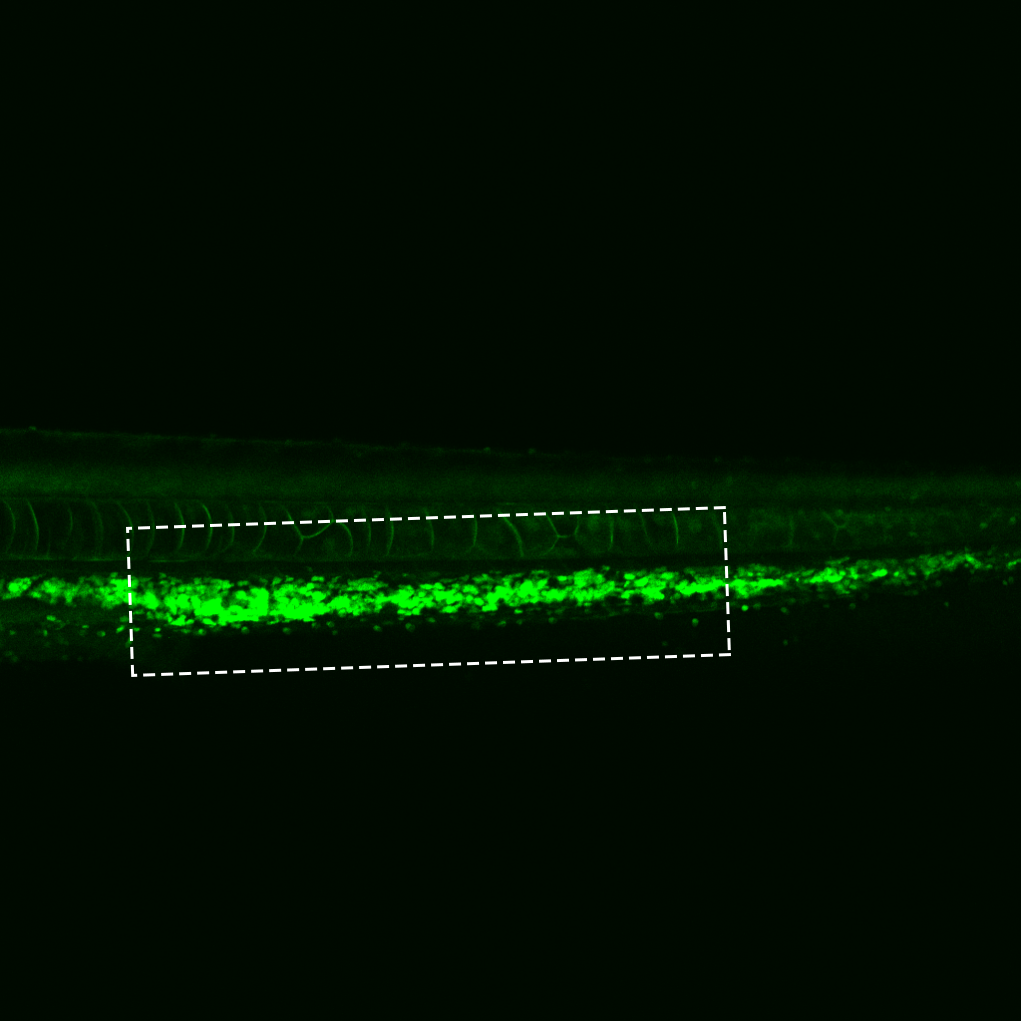

Supplement: Supplementary file 7 — Source data Fig. 2 [file 44318_2024_82_MOESM7_ESM.zip › Figure 2/2J/4dpf GFP sibling.tif]

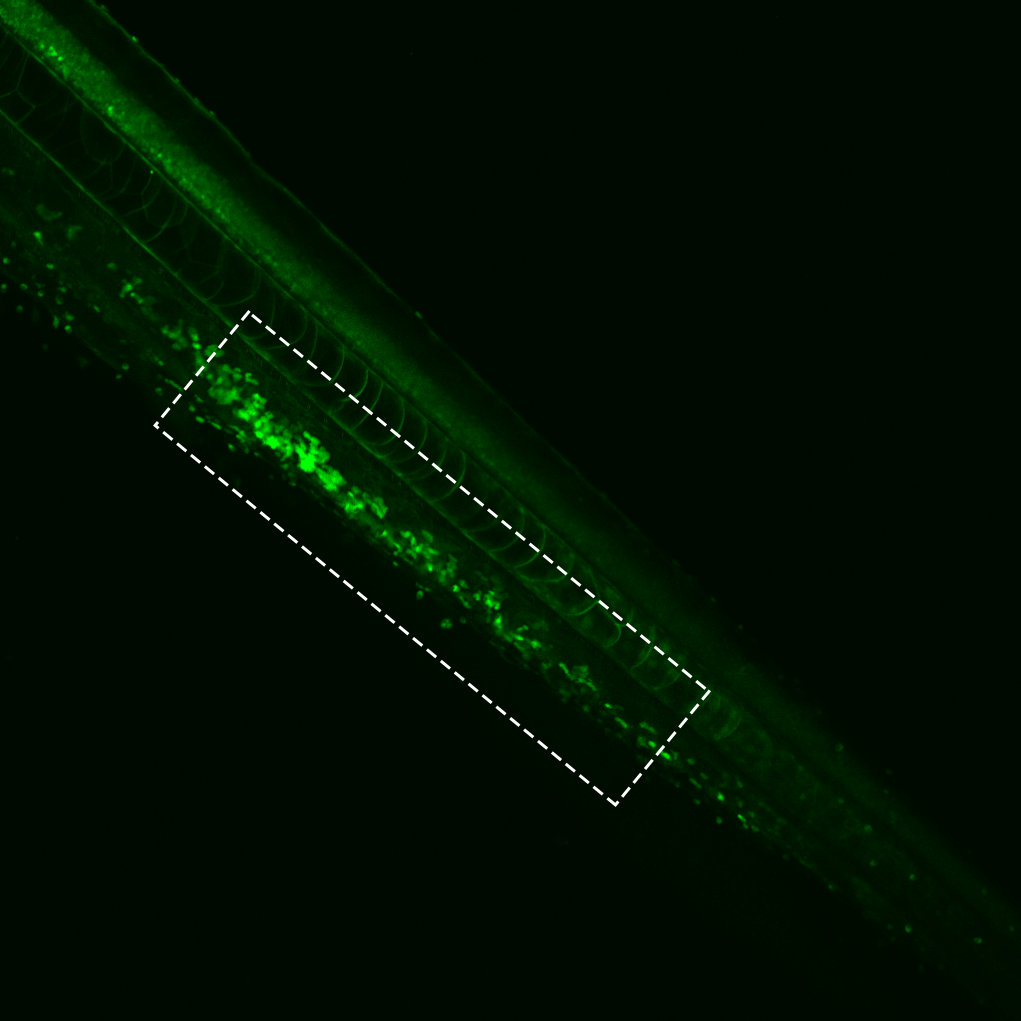

Supplement: Supplementary file 7 — Source data Fig. 2 [file 44318_2024_82_MOESM7_ESM.zip › Figure 2/2J/5dpf GFP mettl16--.tif]

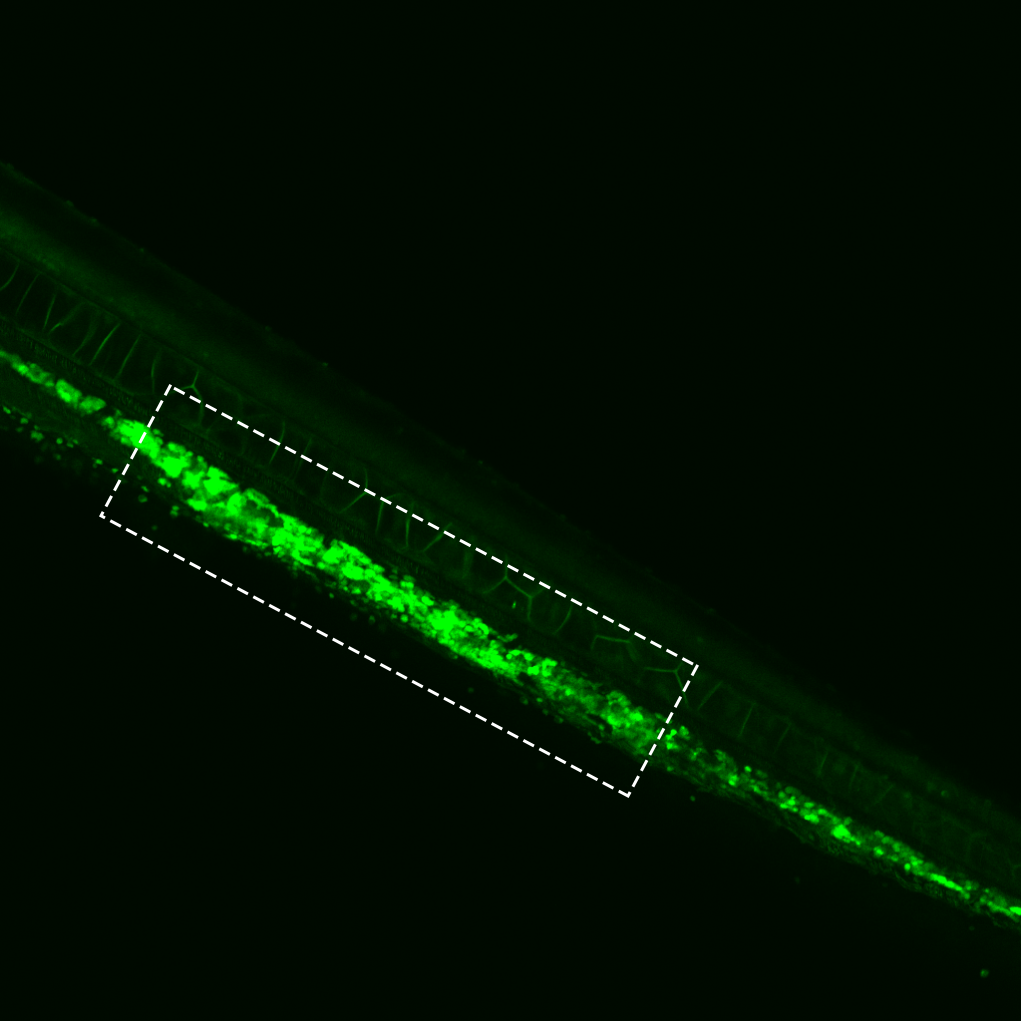

Supplement: Supplementary file 7 — Source data Fig. 2 [file 44318_2024_82_MOESM7_ESM.zip › Figure 2/2J/5dpf GFP sibling.tif]

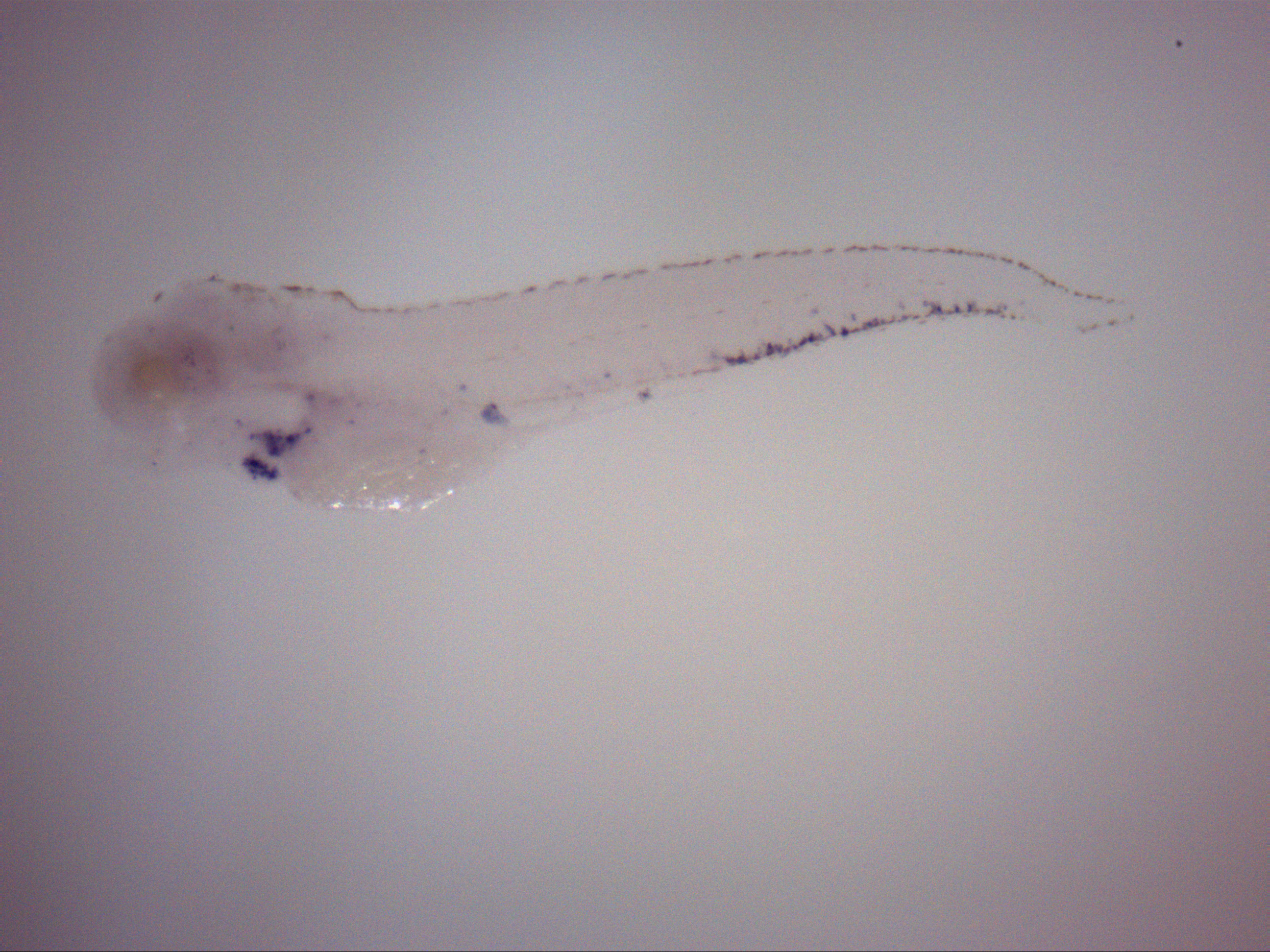

Supplement: Supplementary file 7 — Source data Fig. 2 [file 44318_2024_82_MOESM7_ESM.zip › Figure 2/2L/5d b-globin mettl16--.tif]

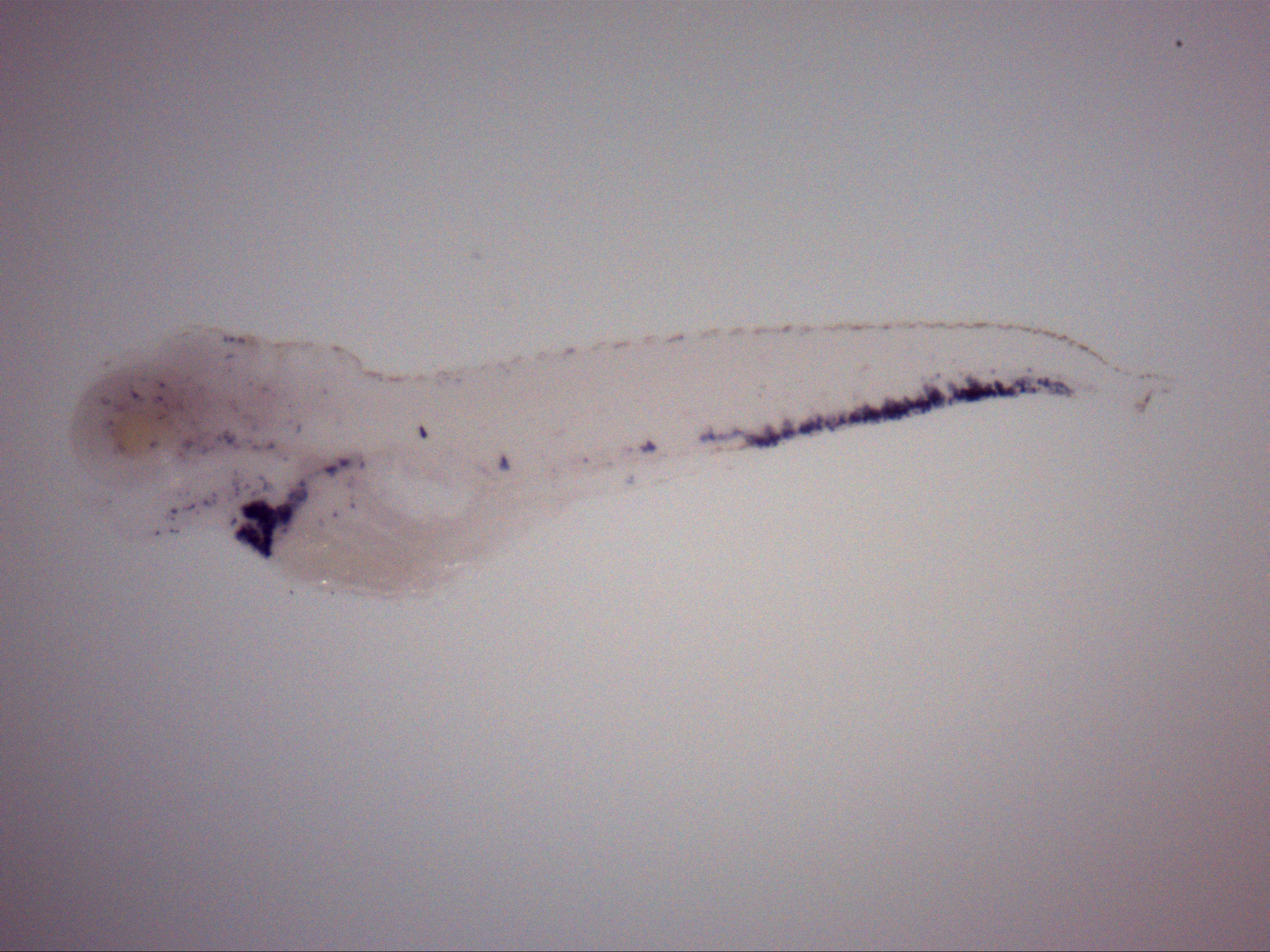

Supplement: Supplementary file 7 — Source data Fig. 2 [file 44318_2024_82_MOESM7_ESM.zip › Figure 2/2L/5d b-globin sibling.tif]

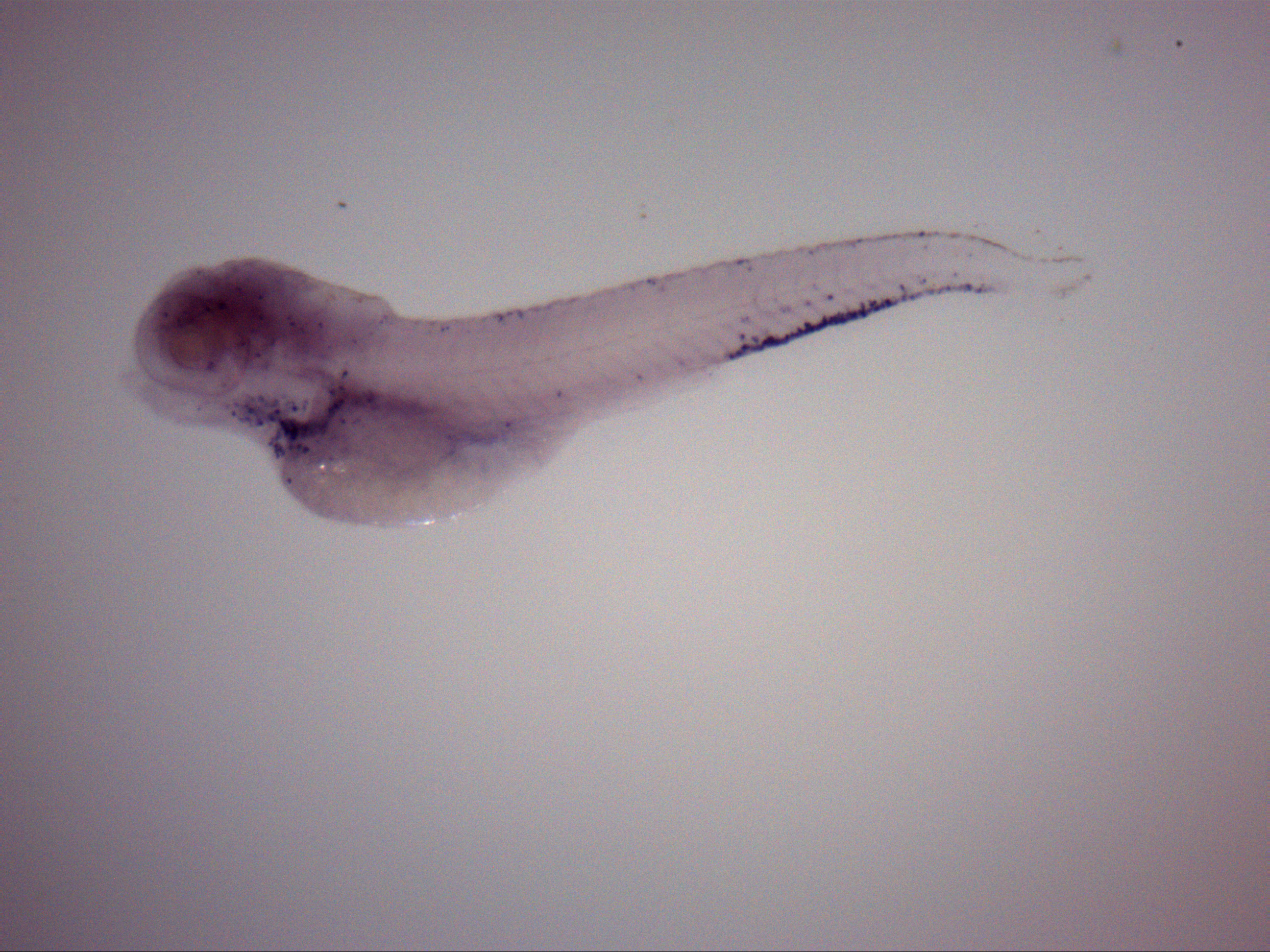

Supplement: Supplementary file 7 — Source data Fig. 2 [file 44318_2024_82_MOESM7_ESM.zip › Figure 2/2L/5d hbae3 mettl16--.tif]

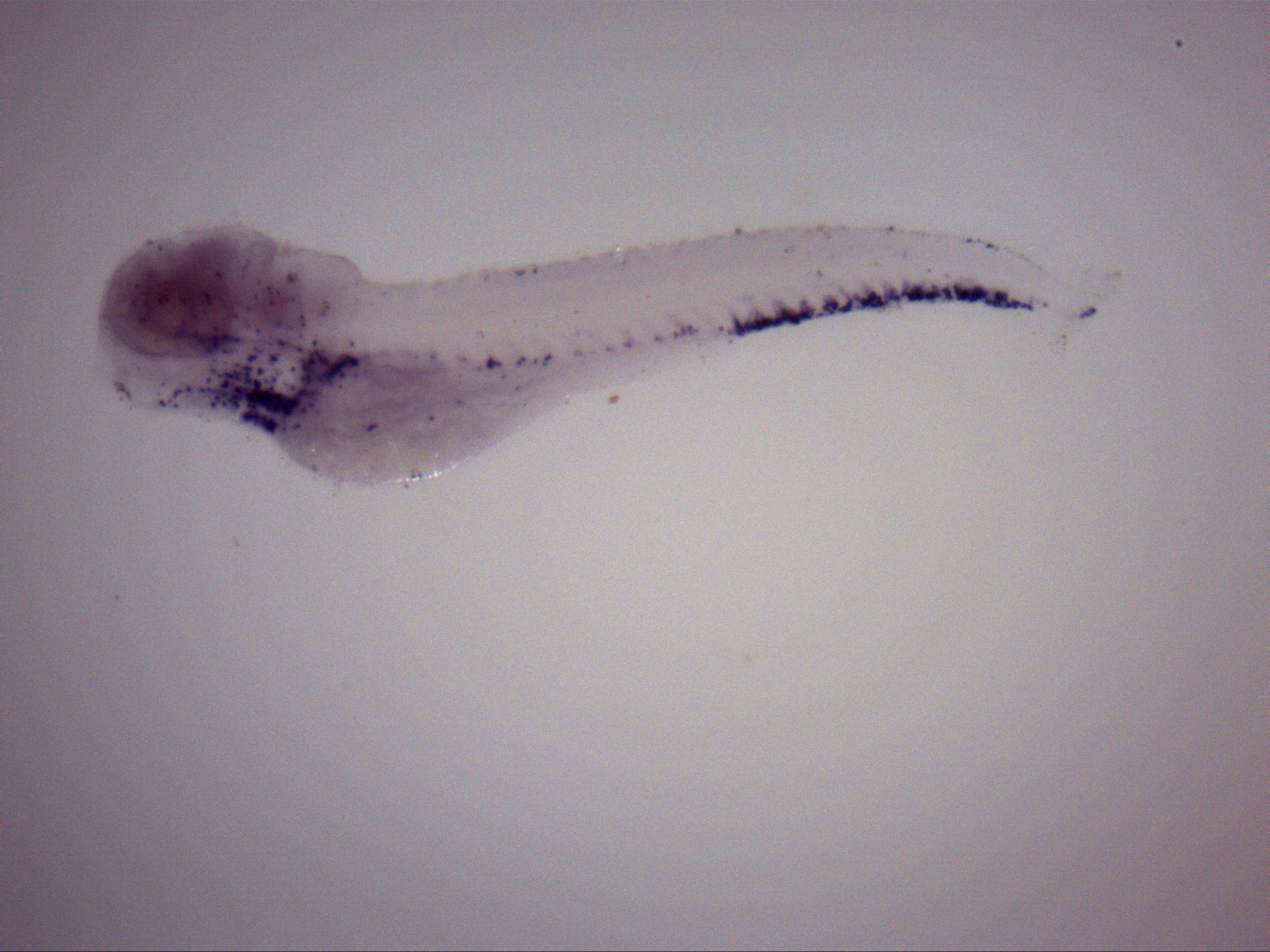

Supplement: Supplementary file 7 — Source data Fig. 2 [file 44318_2024_82_MOESM7_ESM.zip › Figure 2/2L/5d hbae3 sibling.tif]

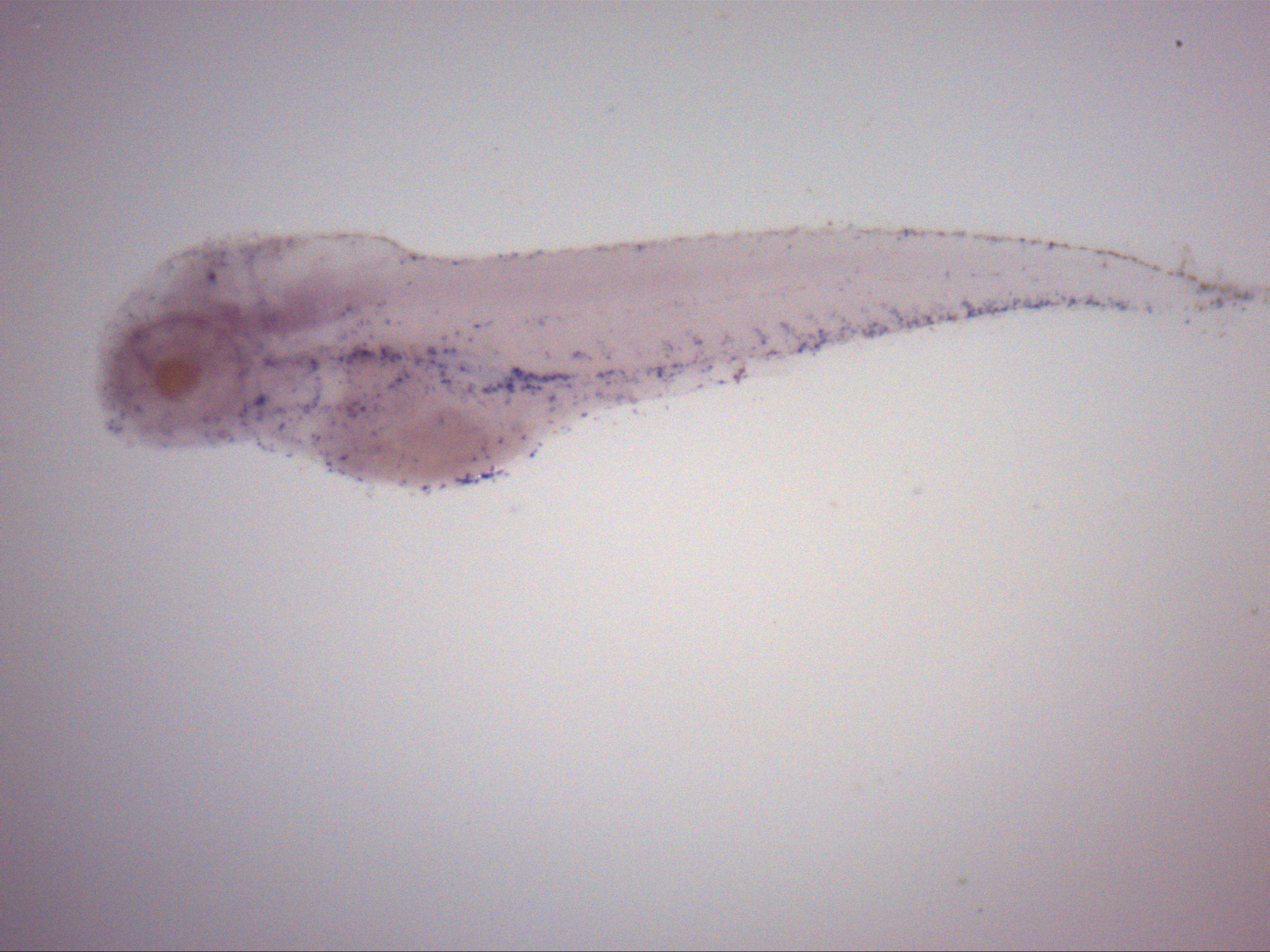

Supplement: Supplementary file 7 — Source data Fig. 2 [file 44318_2024_82_MOESM7_ESM.zip › Figure 2/2L/5d l-plastin mettl16--.tif]

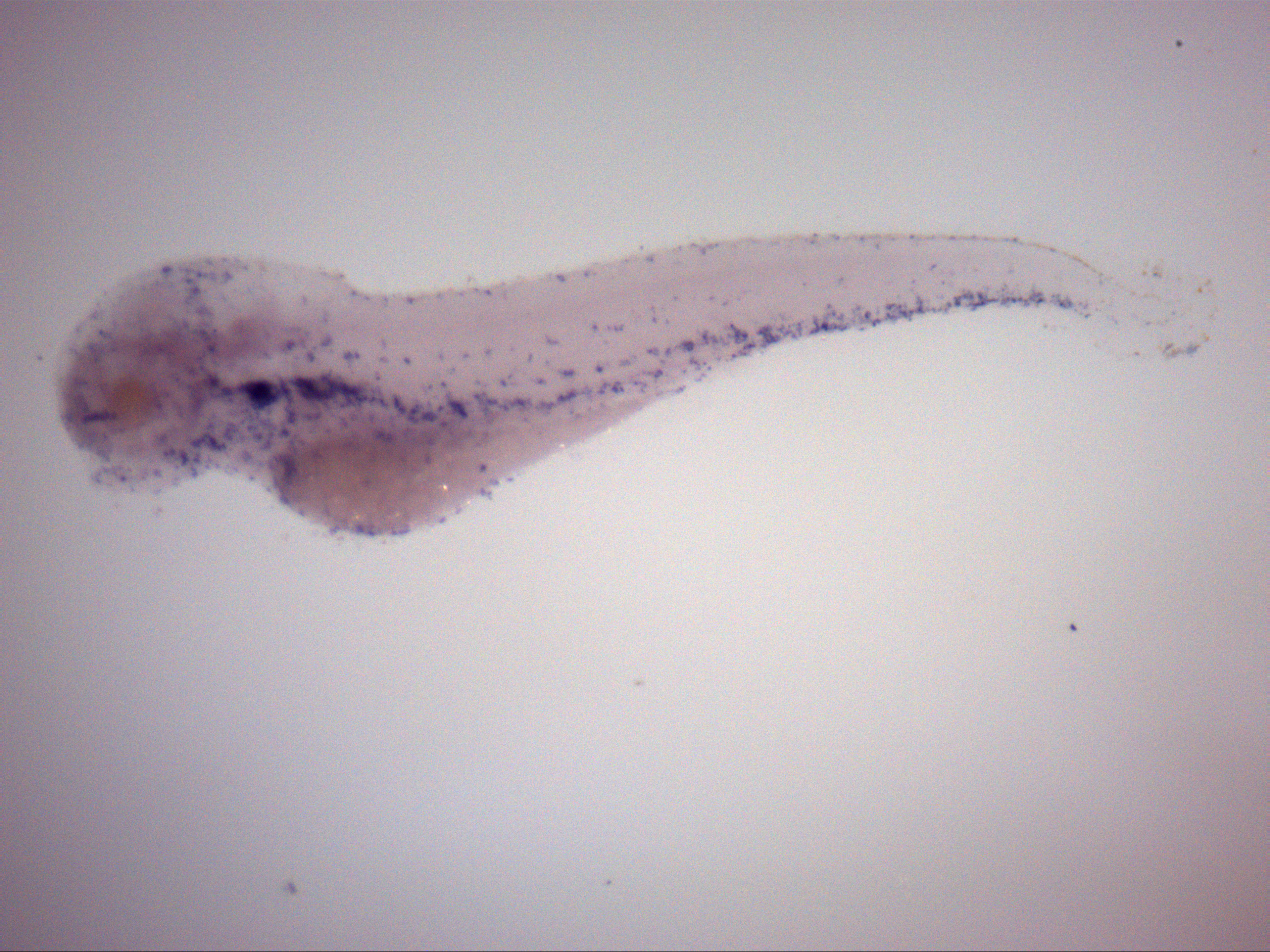

Supplement: Supplementary file 7 — Source data Fig. 2 [file 44318_2024_82_MOESM7_ESM.zip › Figure 2/2L/5d l-plastin sibling.tif]

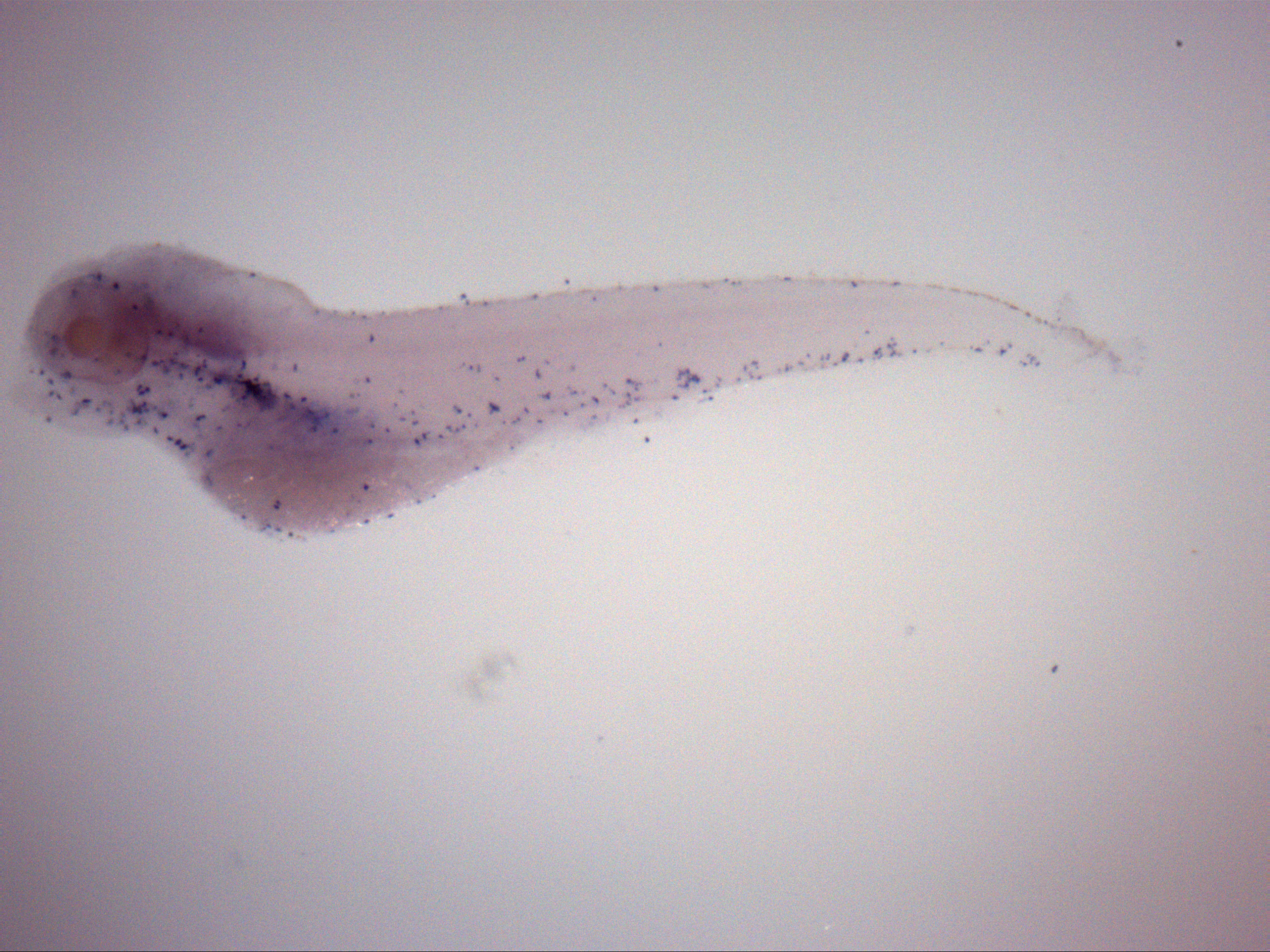

Supplement: Supplementary file 7 — Source data Fig. 2 [file 44318_2024_82_MOESM7_ESM.zip › Figure 2/2L/5d mpx mettl16--.tif]

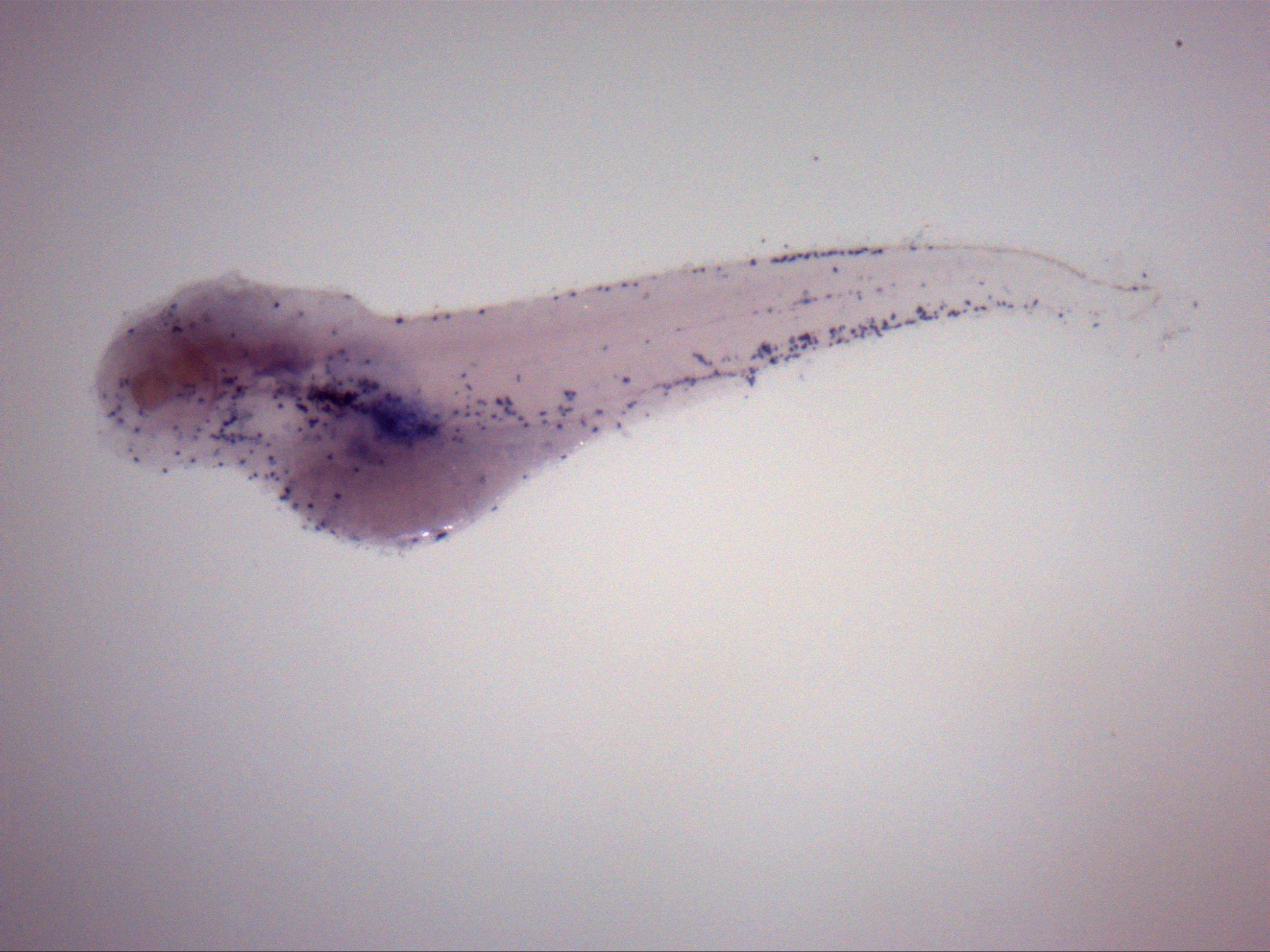

Supplement: Supplementary file 7 — Source data Fig. 2 [file 44318_2024_82_MOESM7_ESM.zip › Figure 2/2L/5d mpx sibling.tif]

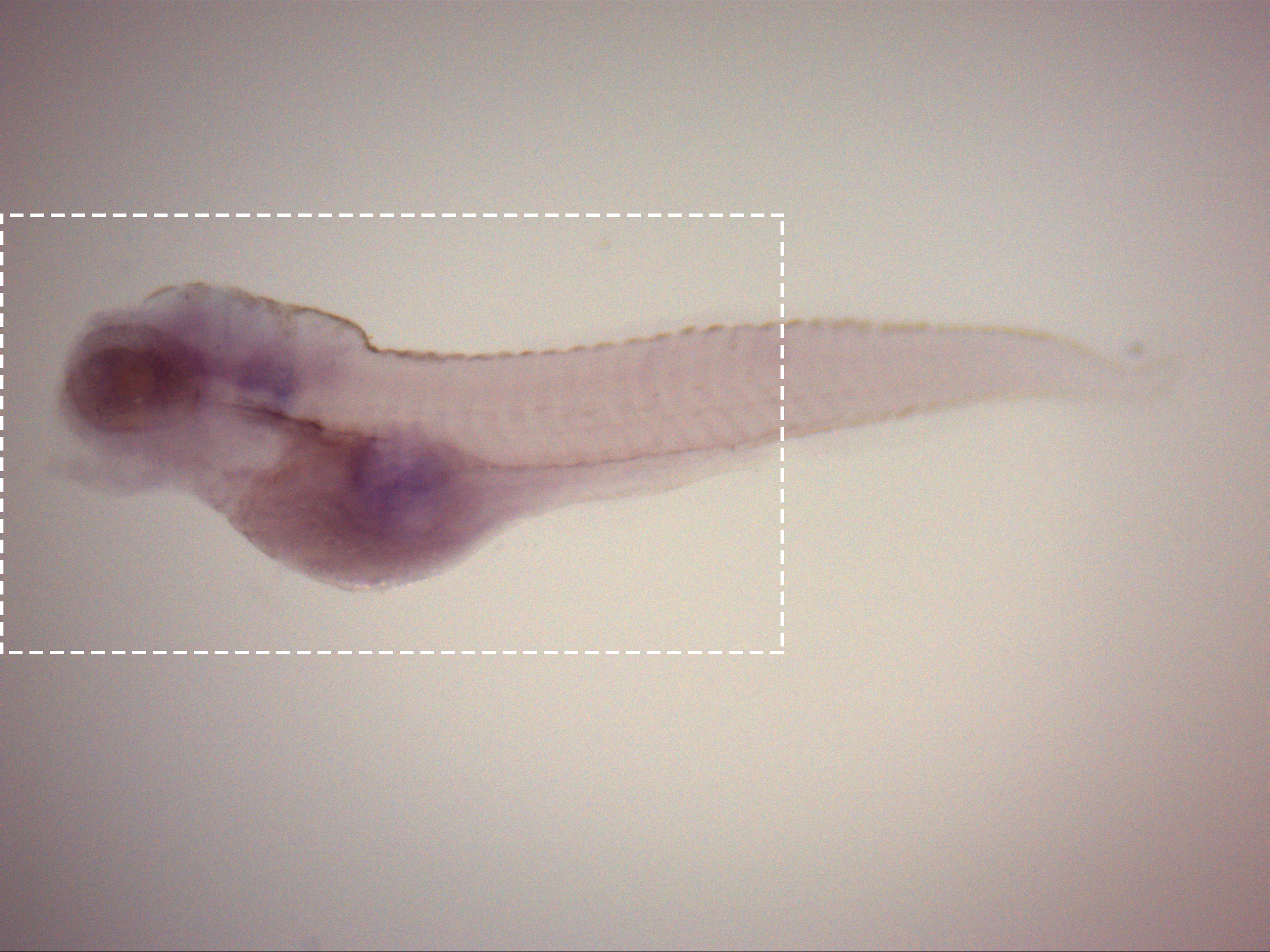

Supplement: Supplementary file 7 — Source data Fig. 2 [file 44318_2024_82_MOESM7_ESM.zip › Figure 2/2L/5dpf rag1 mettl16--.tif]

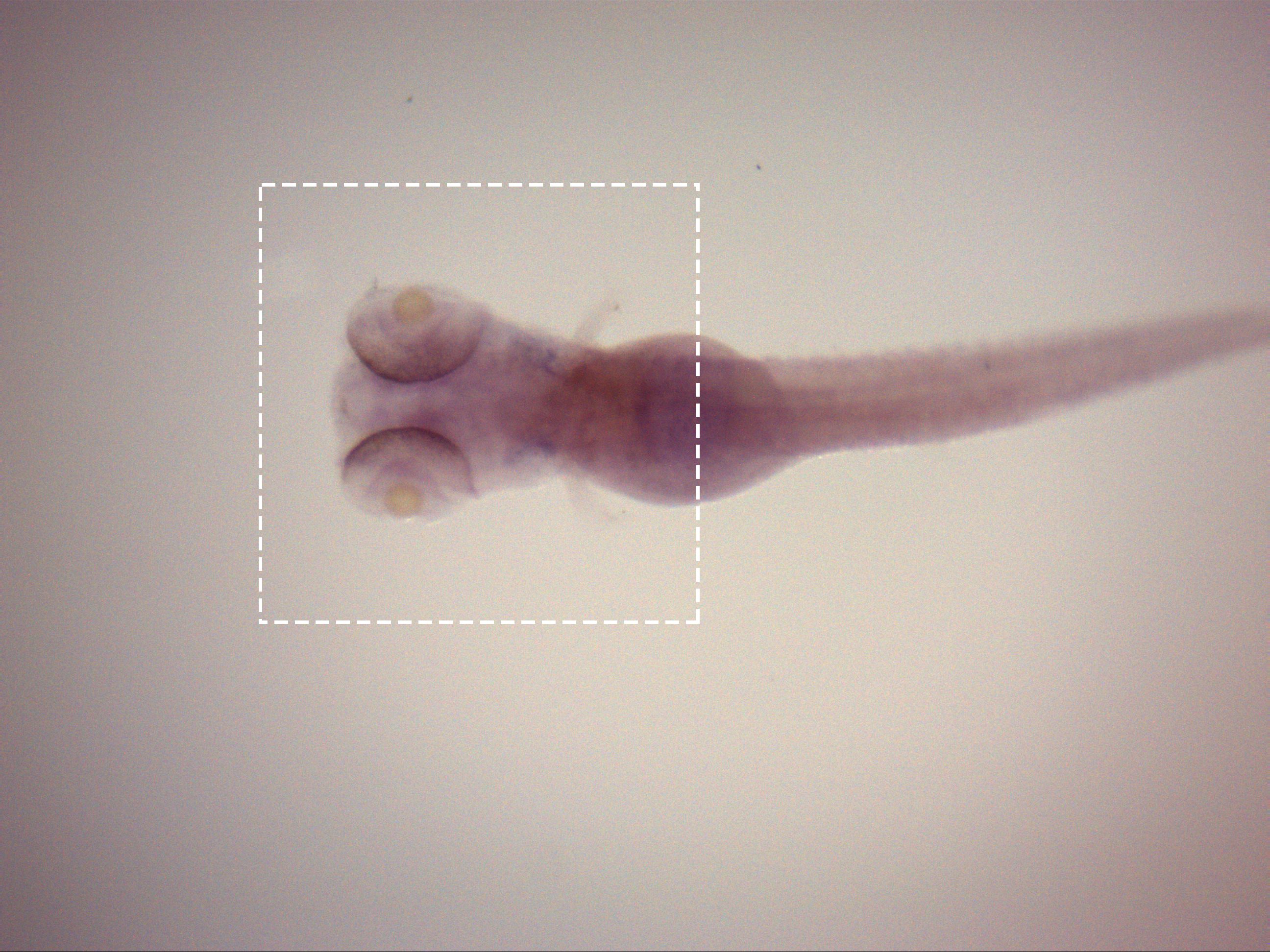

Supplement: Supplementary file 7 — Source data Fig. 2 [file 44318_2024_82_MOESM7_ESM.zip › Figure 2/2L/5dpf rag1 mettl16--1.tif]

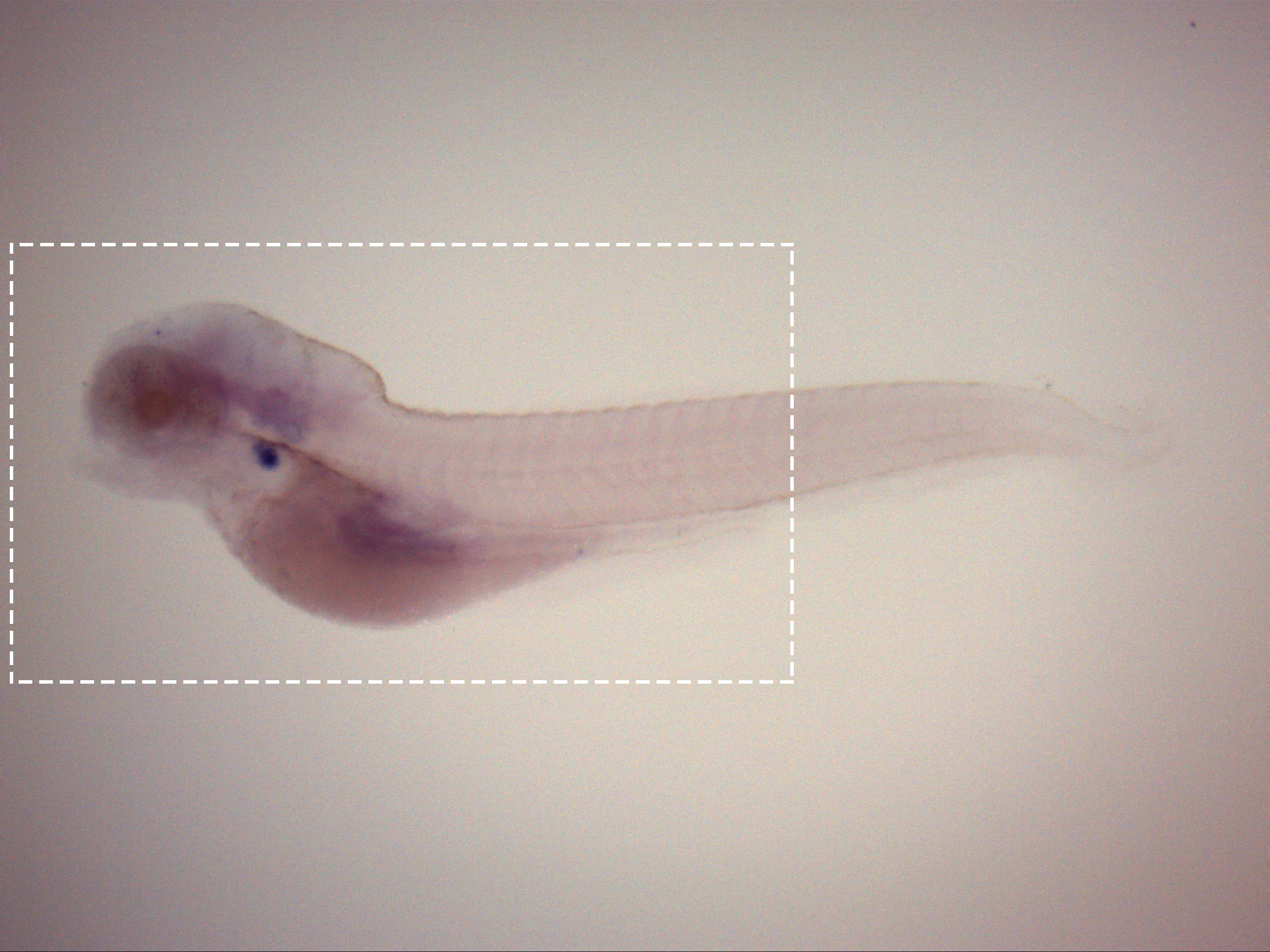

Supplement: Supplementary file 7 — Source data Fig. 2 [file 44318_2024_82_MOESM7_ESM.zip › Figure 2/2L/5dpf rag1 sibling.tif]

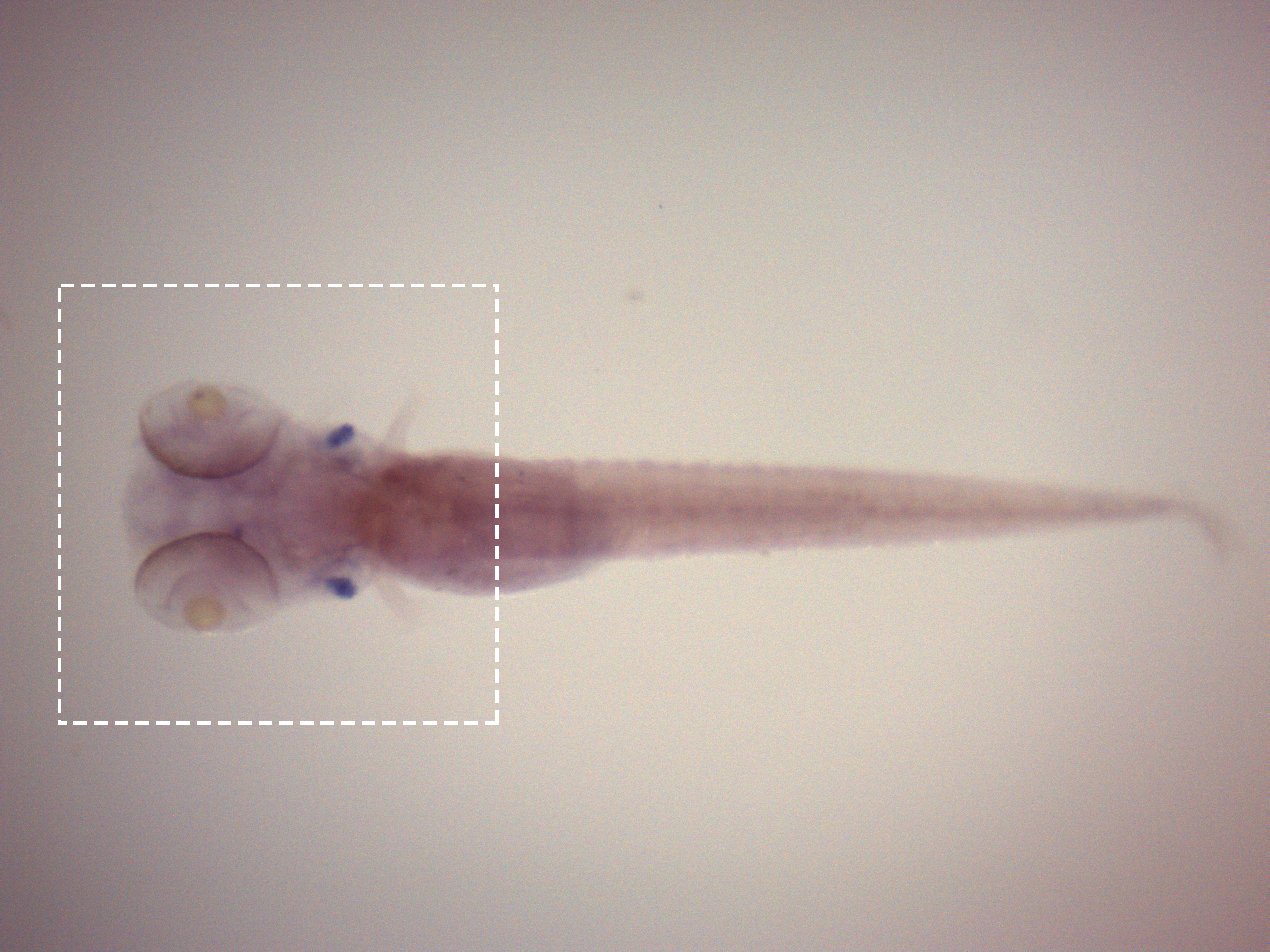

Supplement: Supplementary file 7 — Source data Fig. 2 [file 44318_2024_82_MOESM7_ESM.zip › Figure 2/2L/5dpf rag1 sibling-1.tif]

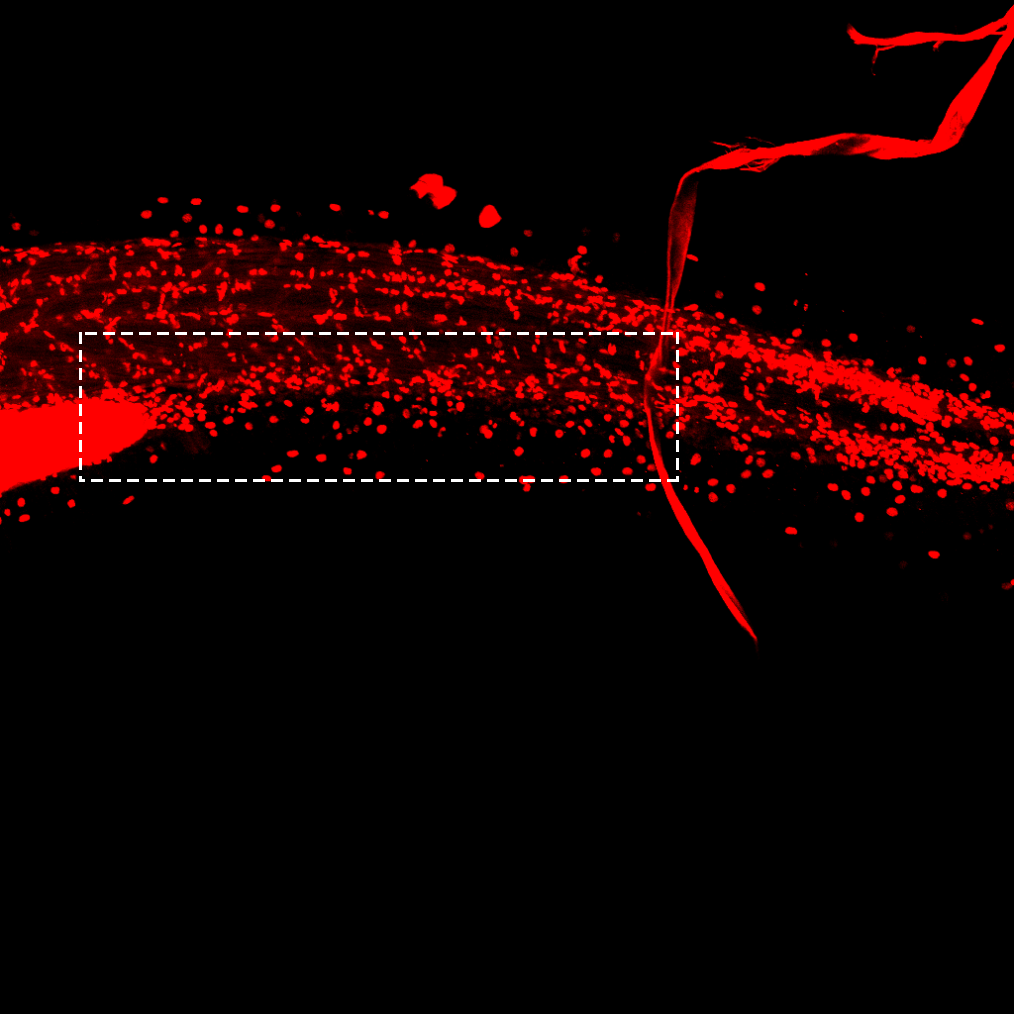

Supplement: Supplementary file 8 — Source data Fig. 3 [file 44318_2024_82_MOESM8_ESM.zip › Figure 3/3A/2dpf mettl16-- EDU.tif]

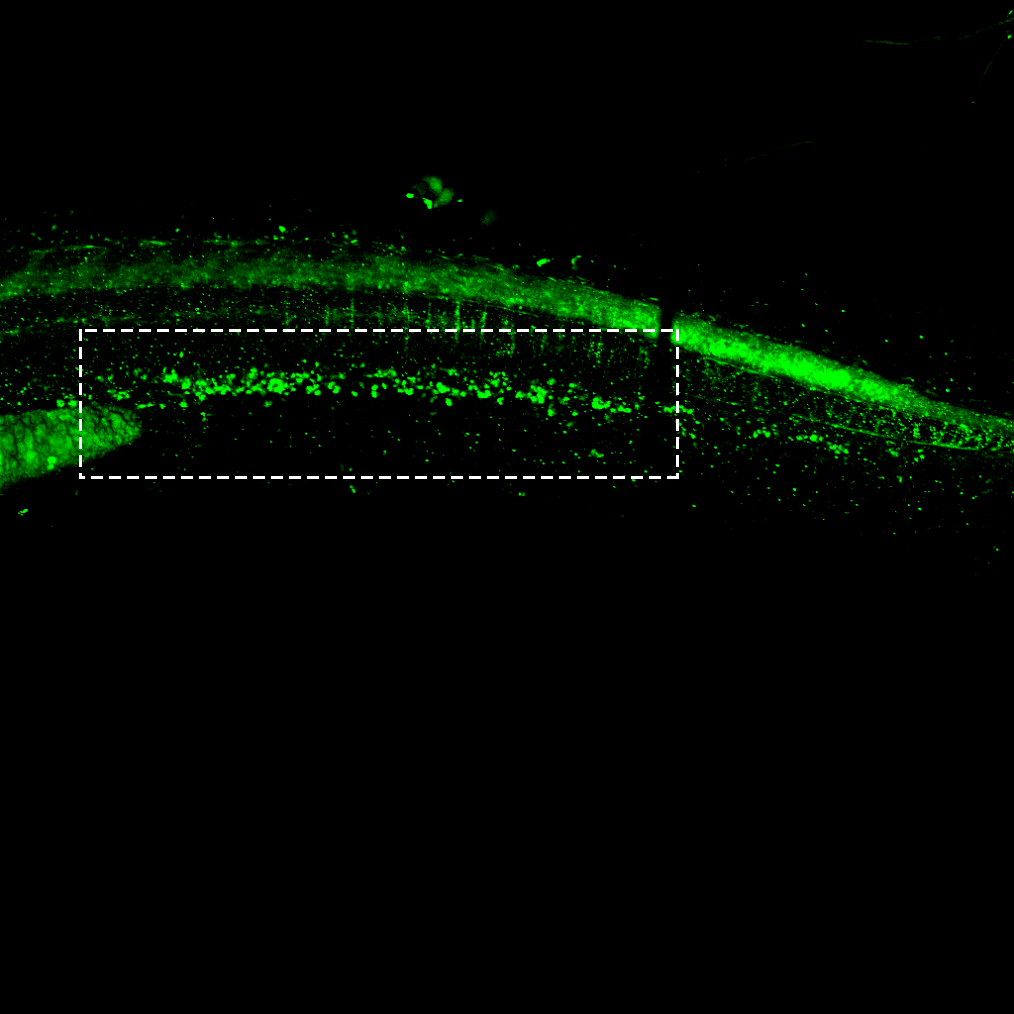

Supplement: Supplementary file 8 — Source data Fig. 3 [file 44318_2024_82_MOESM8_ESM.zip › Figure 3/3A/2dpf mettl16-- GFP.tif]

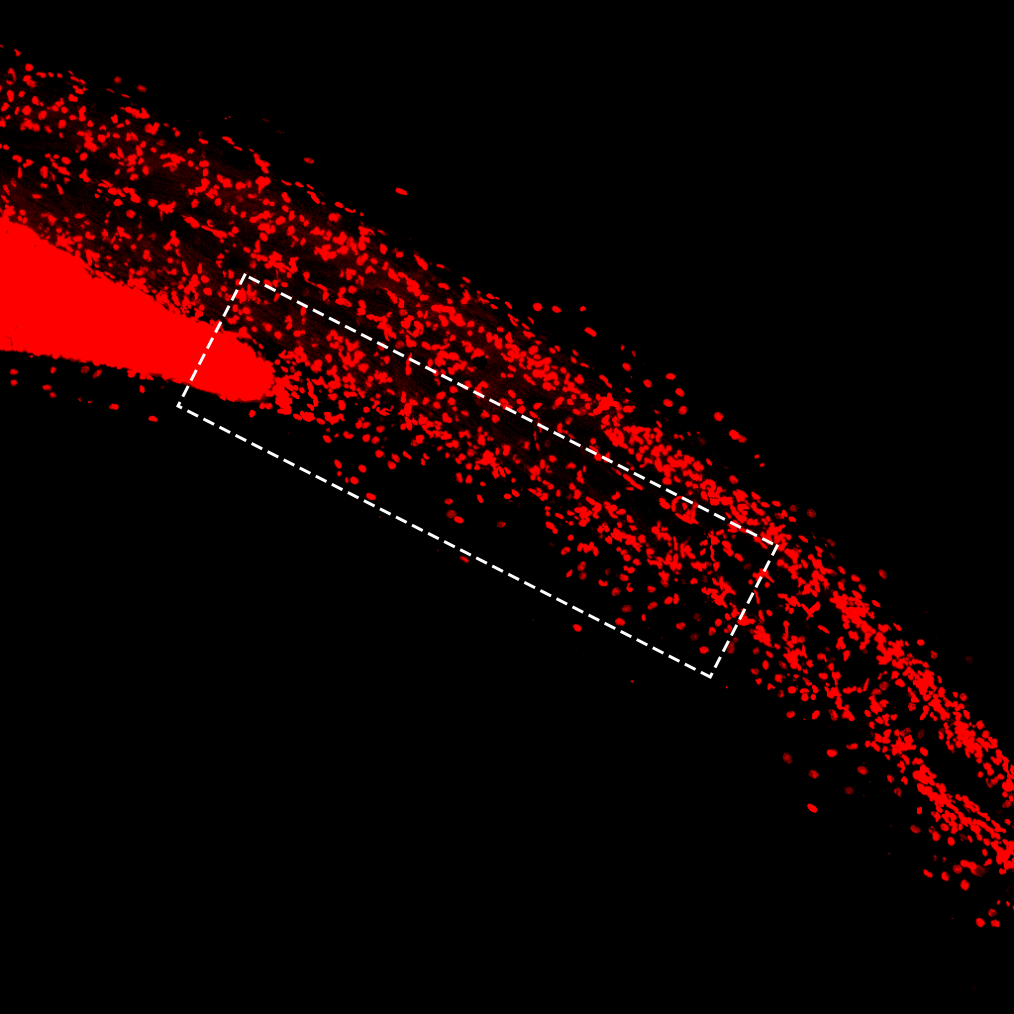

Supplement: Supplementary file 8 — Source data Fig. 3 [file 44318_2024_82_MOESM8_ESM.zip › Figure 3/3A/2dpf sibling EDU.tif]

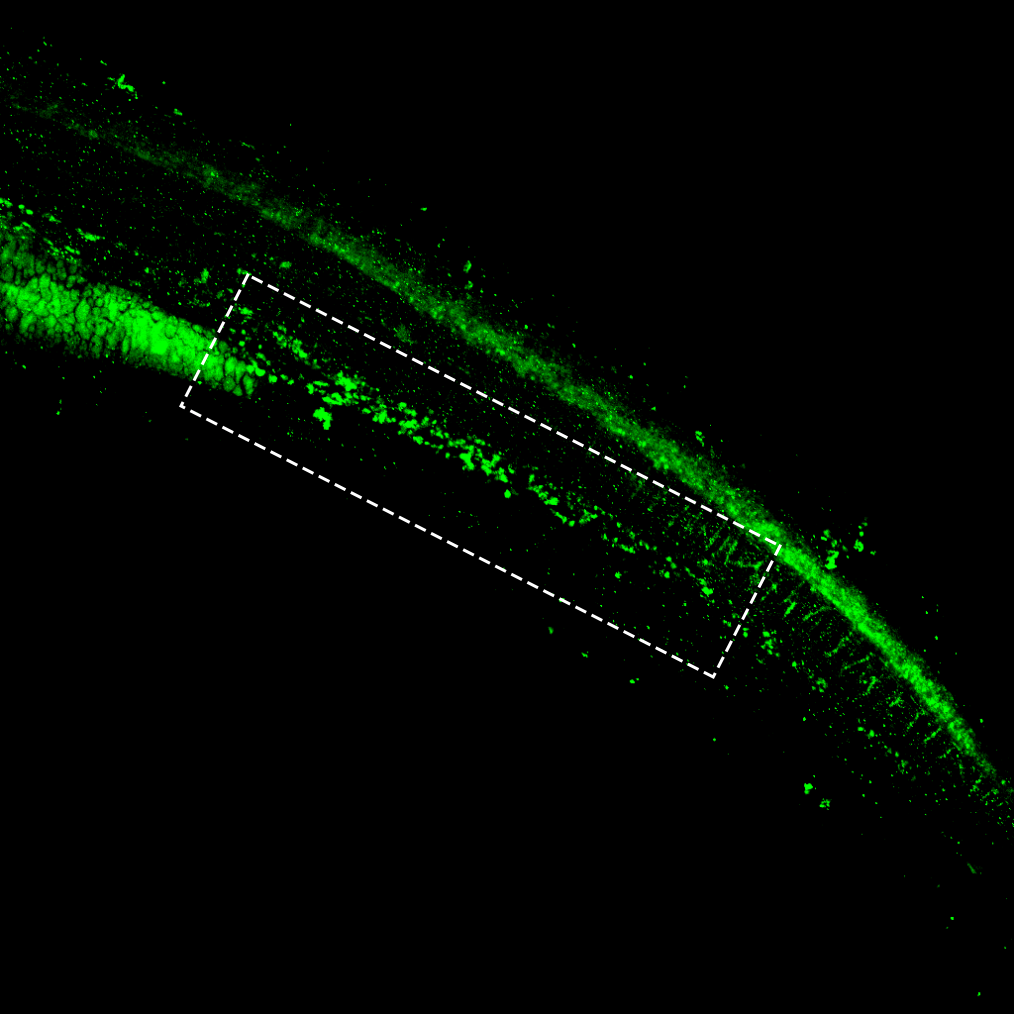

Supplement: Supplementary file 8 — Source data Fig. 3 [file 44318_2024_82_MOESM8_ESM.zip › Figure 3/3A/2dpf sibling GFP.tif]

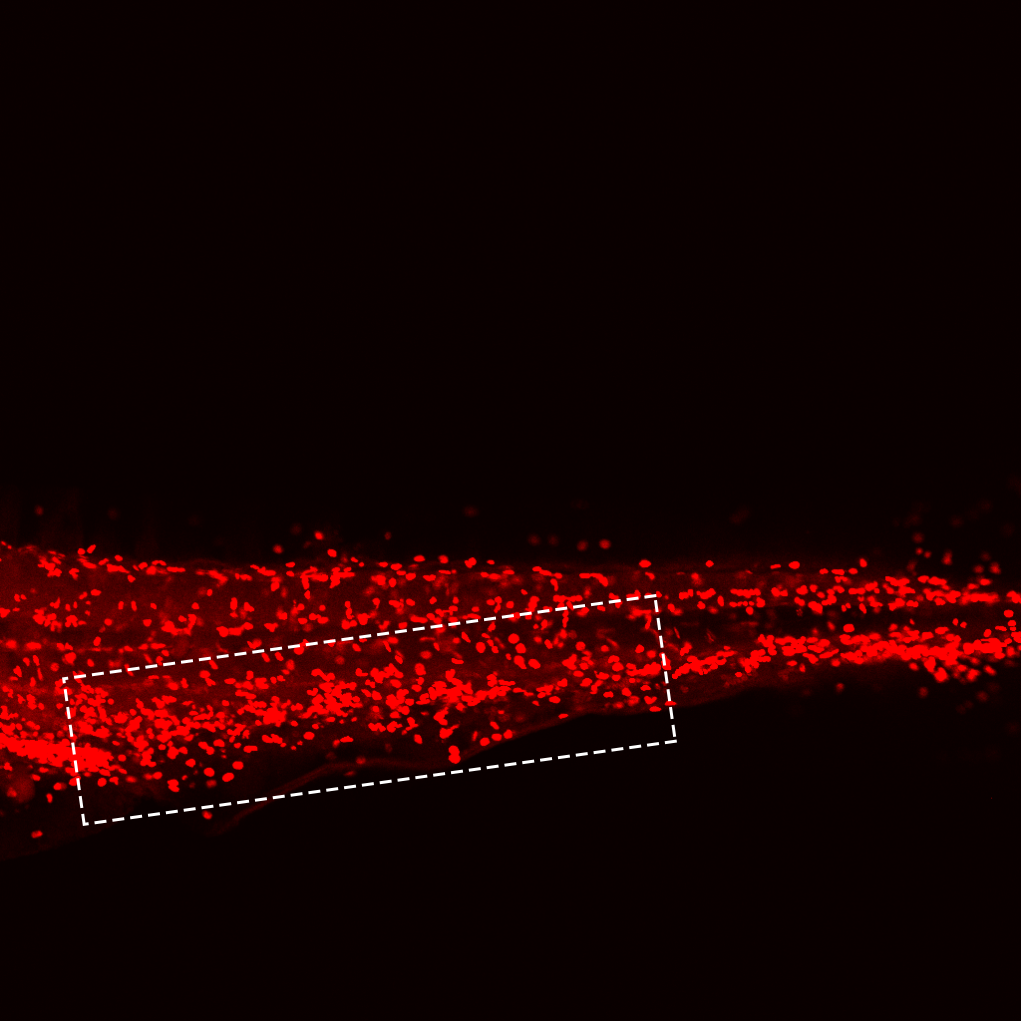

Supplement: Supplementary file 8 — Source data Fig. 3 [file 44318_2024_82_MOESM8_ESM.zip › Figure 3/3A/3dpf mettl16-- EDU.tif]

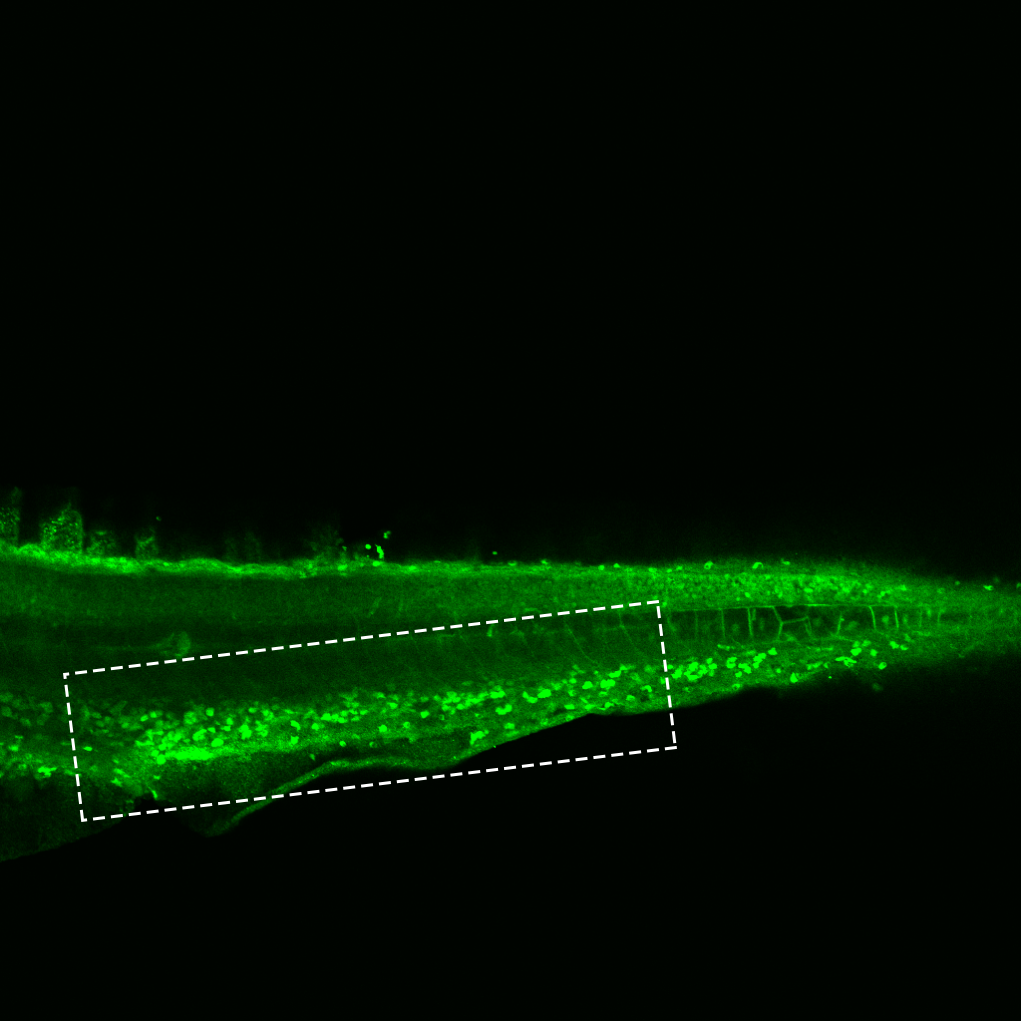

Supplement: Supplementary file 8 — Source data Fig. 3 [file 44318_2024_82_MOESM8_ESM.zip › Figure 3/3A/3dpf mettl16-- GFP.tif]

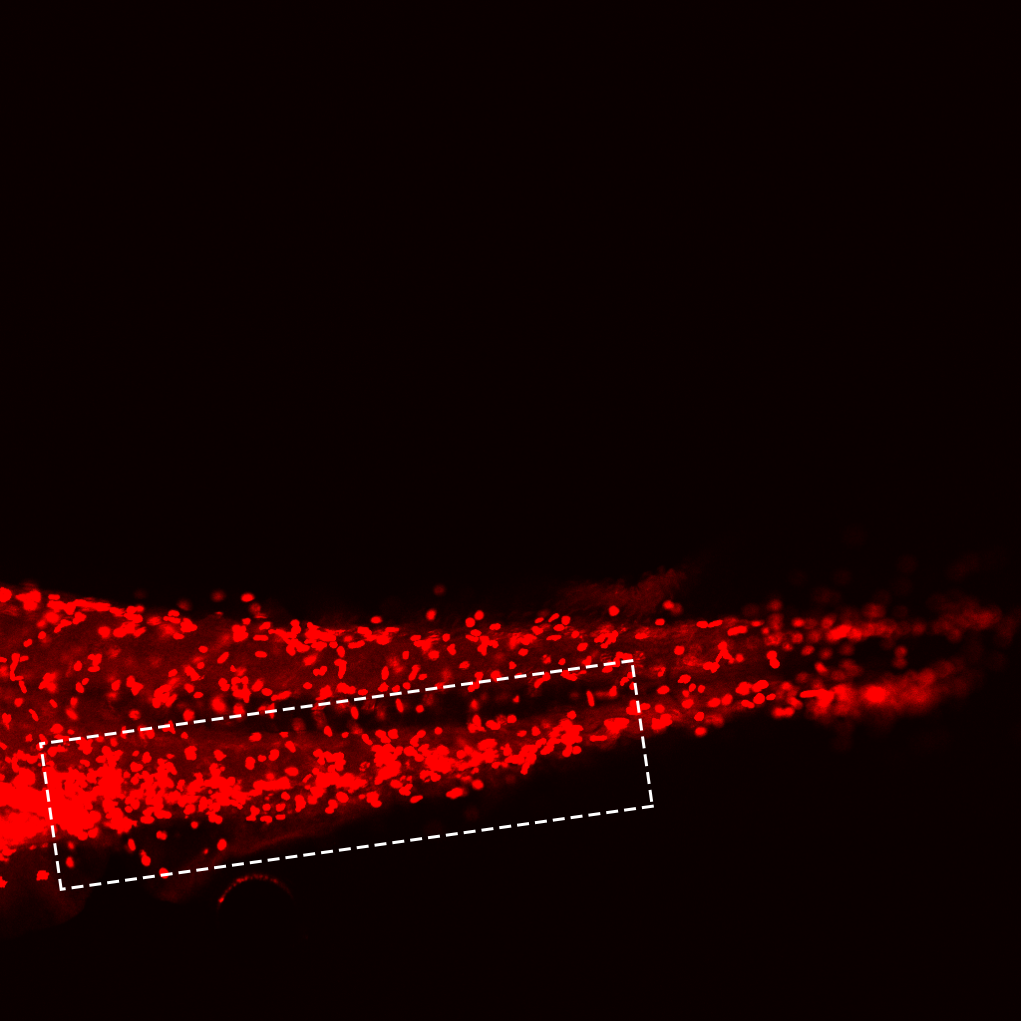

Supplement: Supplementary file 8 — Source data Fig. 3 [file 44318_2024_82_MOESM8_ESM.zip › Figure 3/3A/3dpf sibling EDU.tif]

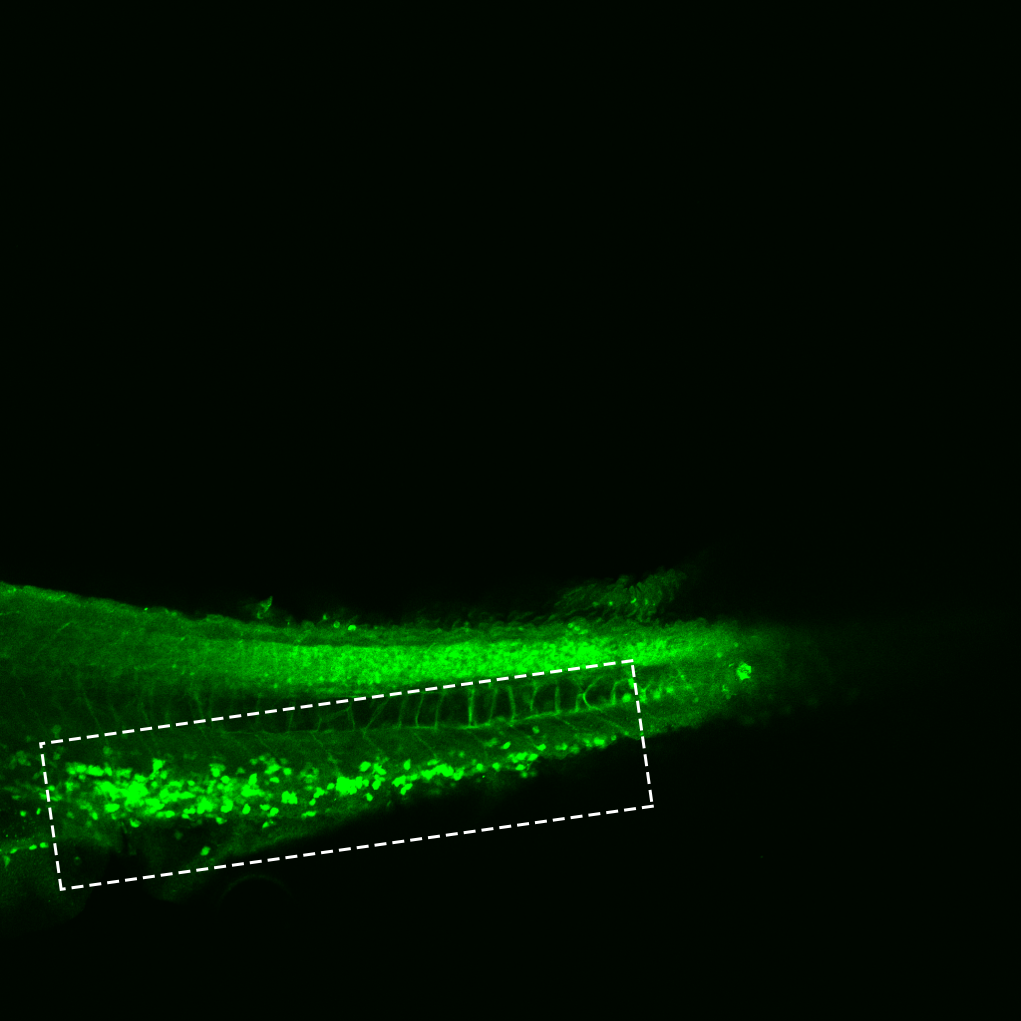

Supplement: Supplementary file 8 — Source data Fig. 3 [file 44318_2024_82_MOESM8_ESM.zip › Figure 3/3A/3dpf sibling GFP.tif]

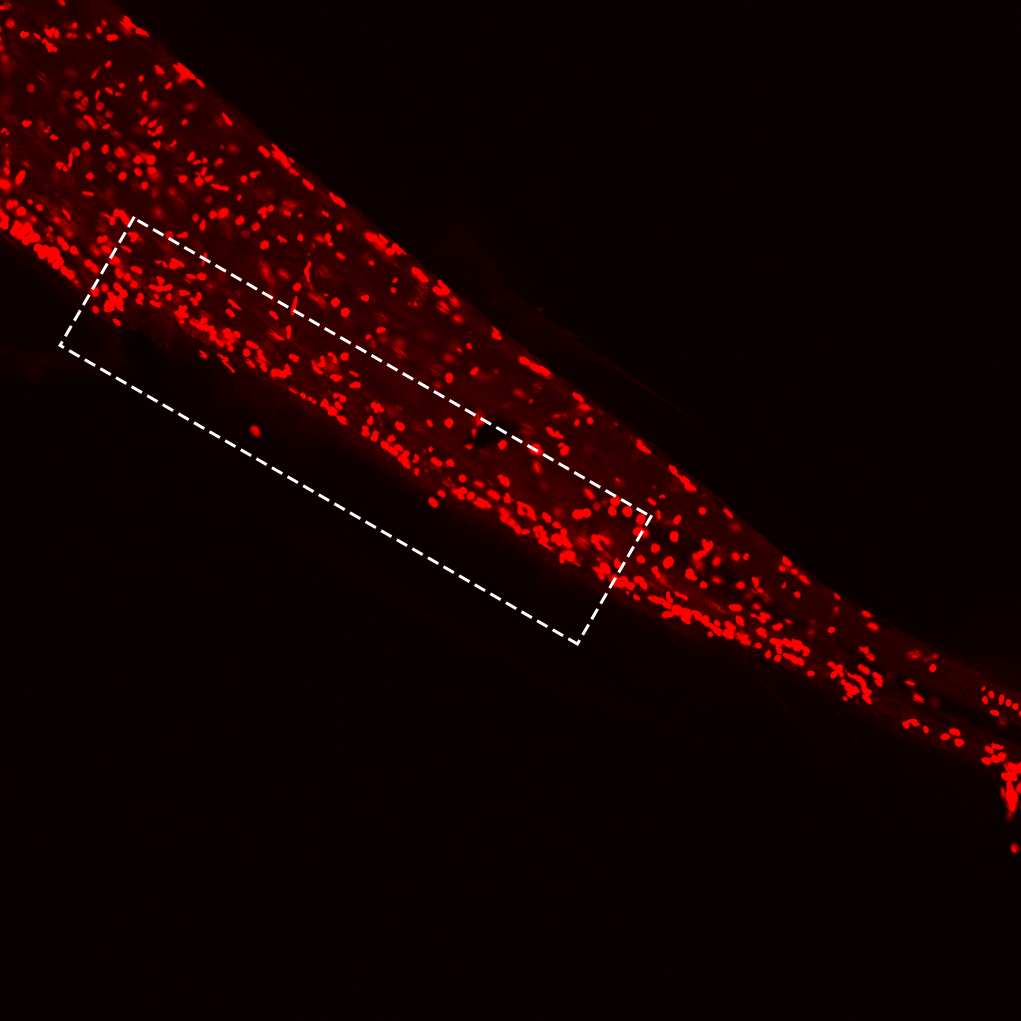

Supplement: Supplementary file 8 — Source data Fig. 3 [file 44318_2024_82_MOESM8_ESM.zip › Figure 3/3A/4dpf mettl16-- EDU.tif]

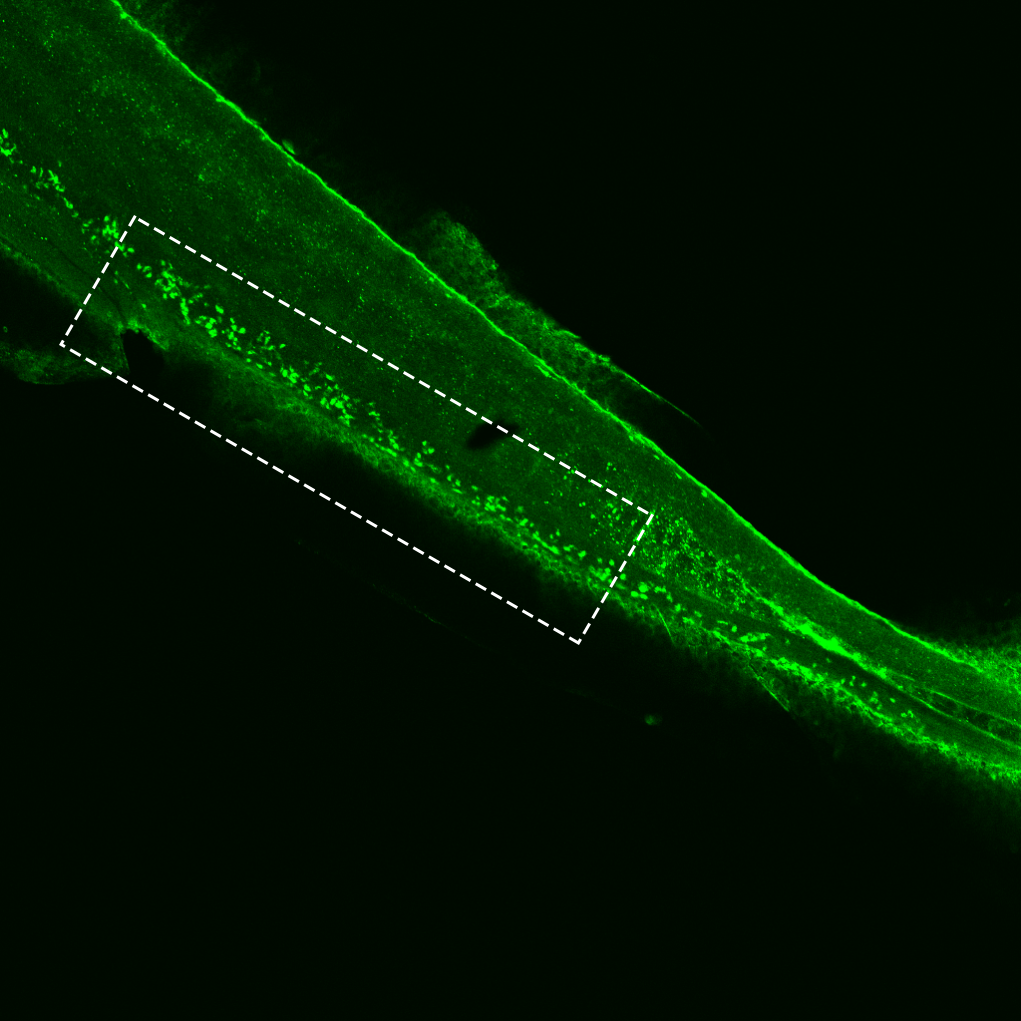

Supplement: Supplementary file 8 — Source data Fig. 3 [file 44318_2024_82_MOESM8_ESM.zip › Figure 3/3A/4dpf mettl16-- GFP.tif]

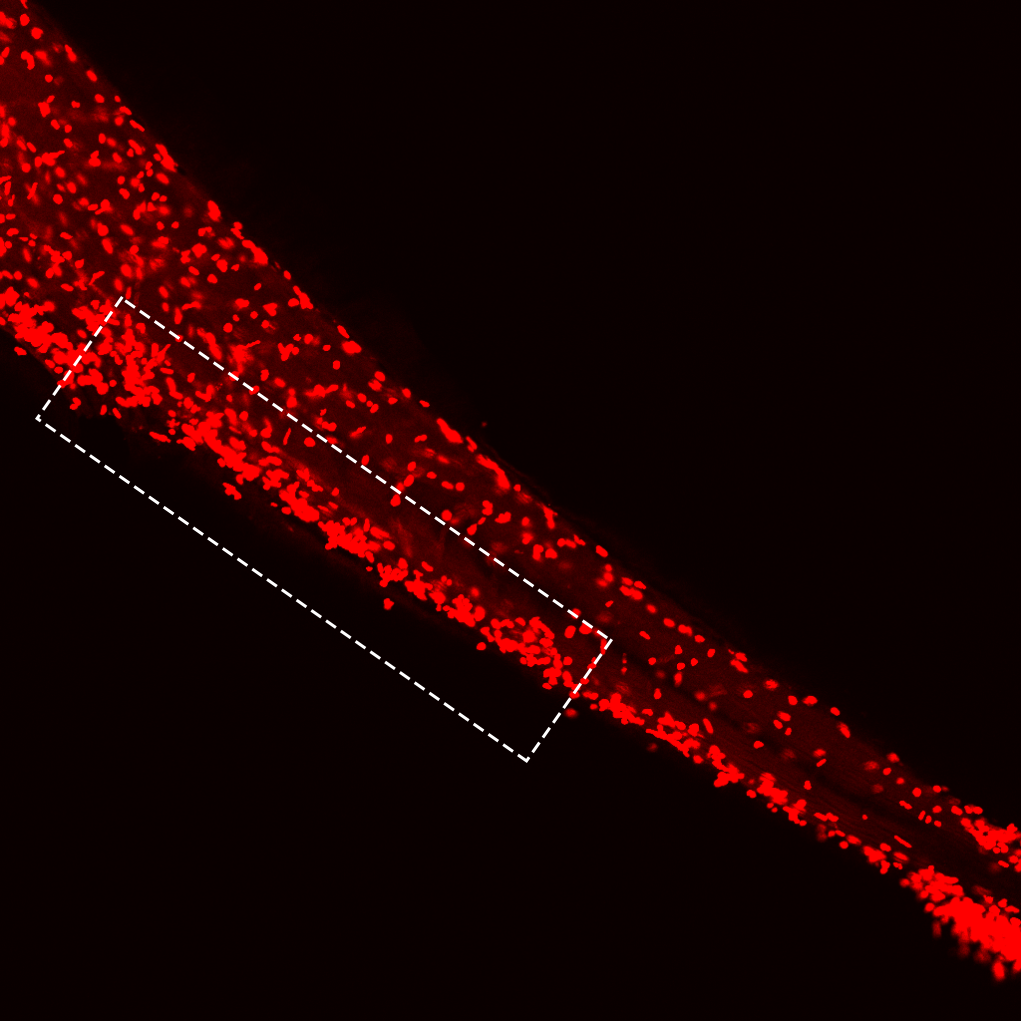

Supplement: Supplementary file 8 — Source data Fig. 3 [file 44318_2024_82_MOESM8_ESM.zip › Figure 3/3A/4dpf sibling EDU.tif]

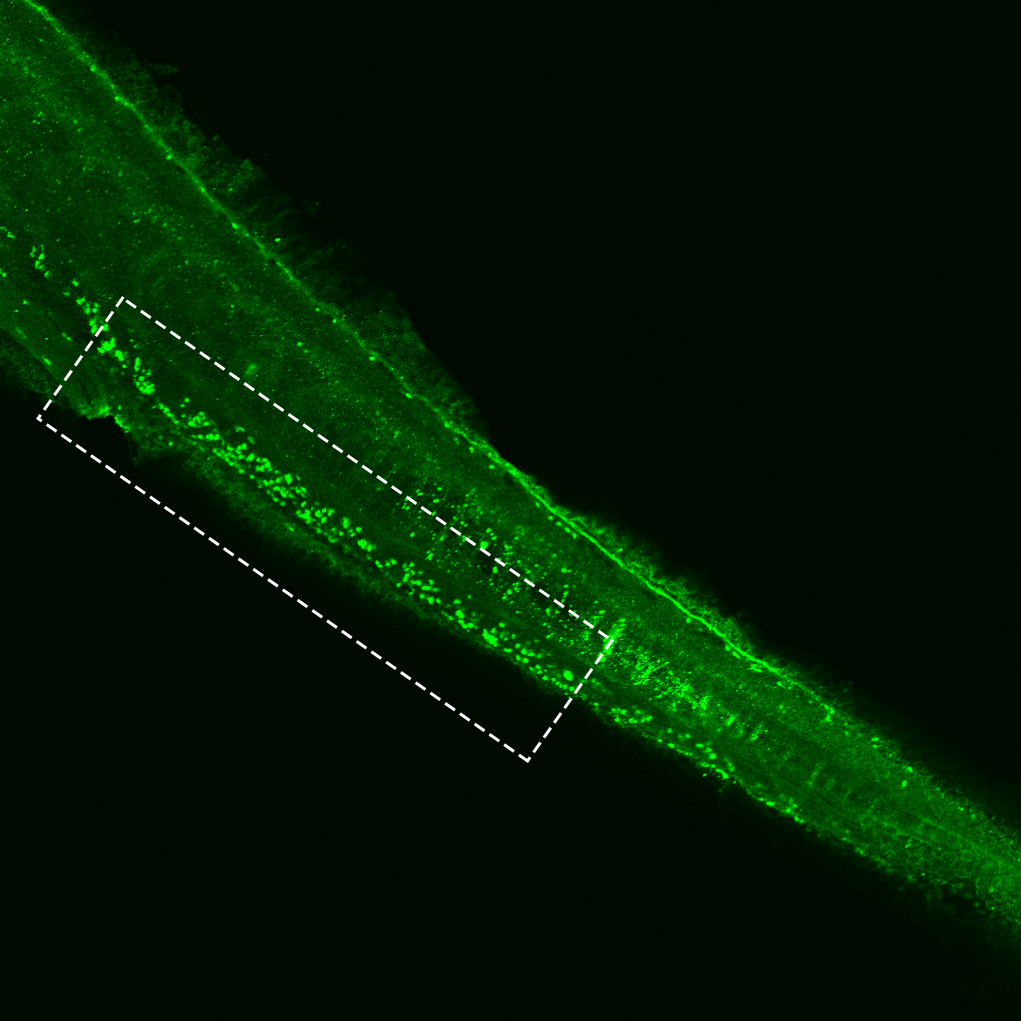

Supplement: Supplementary file 8 — Source data Fig. 3 [file 44318_2024_82_MOESM8_ESM.zip › Figure 3/3A/4dpf sibling GFP.tif]

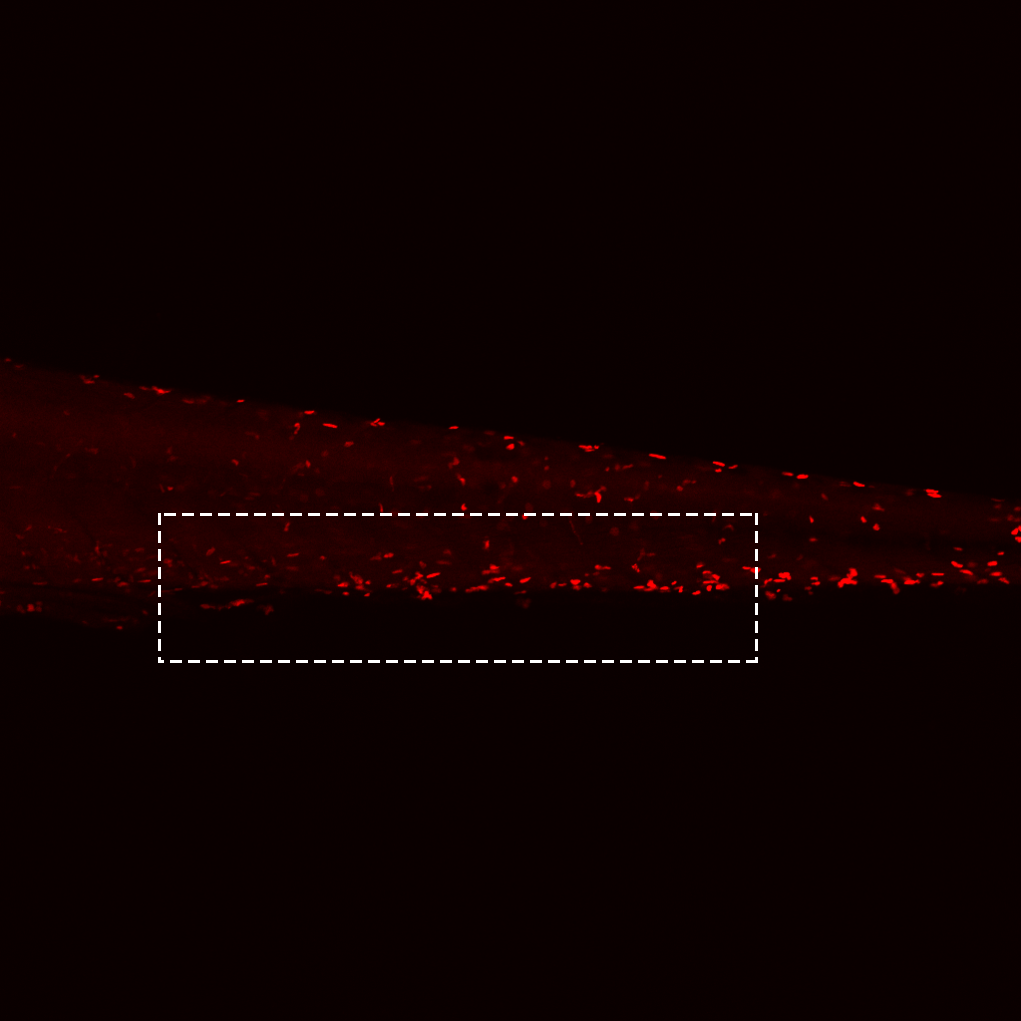

Supplement: Supplementary file 8 — Source data Fig. 3 [file 44318_2024_82_MOESM8_ESM.zip › Figure 3/3A/5dpf mettl16-- EDU.tif]

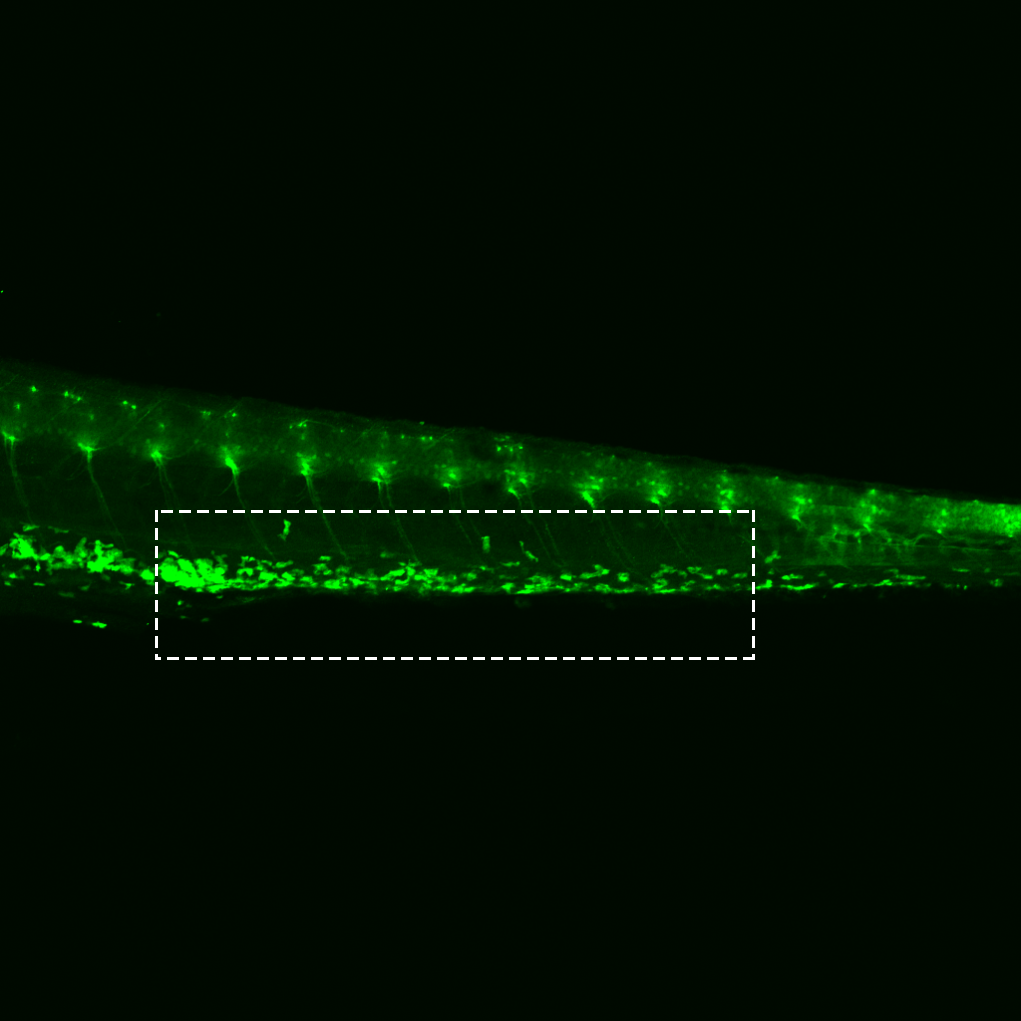

Supplement: Supplementary file 8 — Source data Fig. 3 [file 44318_2024_82_MOESM8_ESM.zip › Figure 3/3A/5dpf mettl16-- GFP.tif]

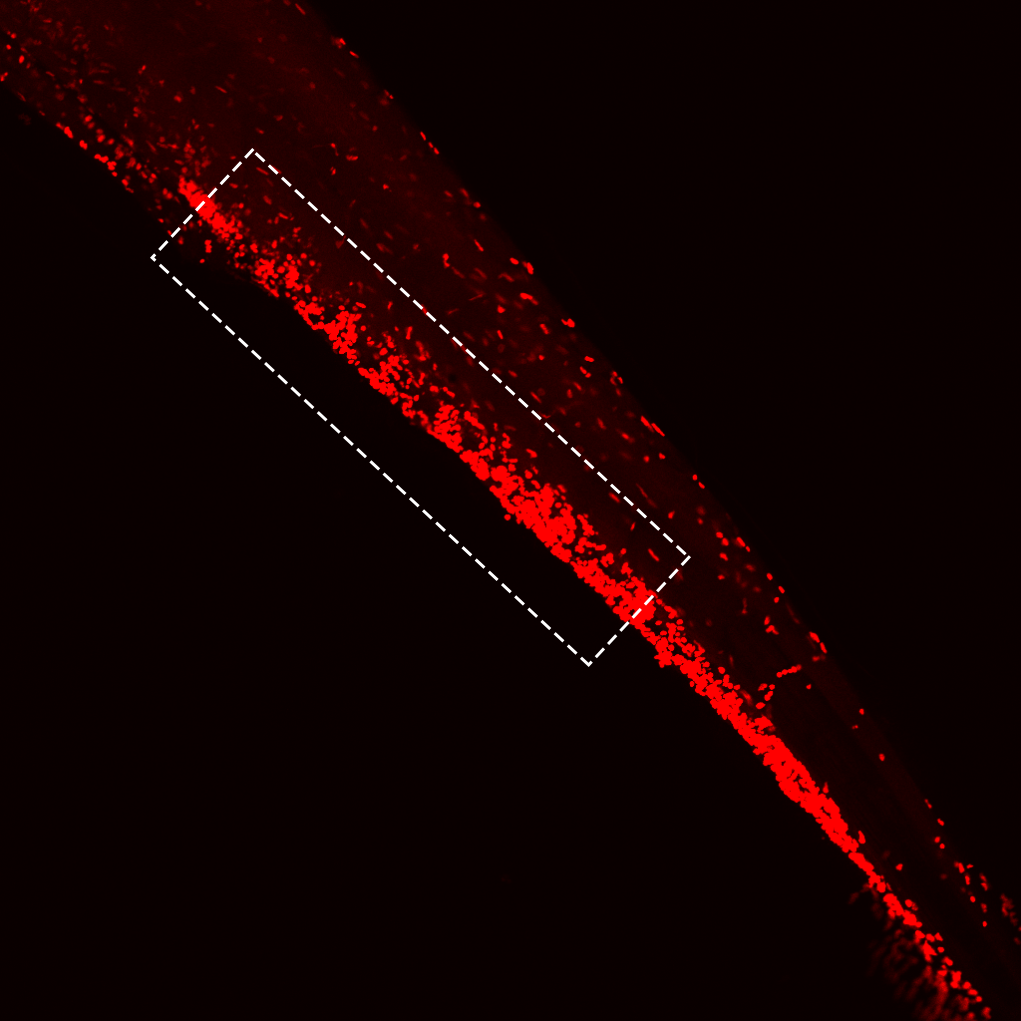

Supplement: Supplementary file 8 — Source data Fig. 3 [file 44318_2024_82_MOESM8_ESM.zip › Figure 3/3A/5dpf sibling EDU.tif]

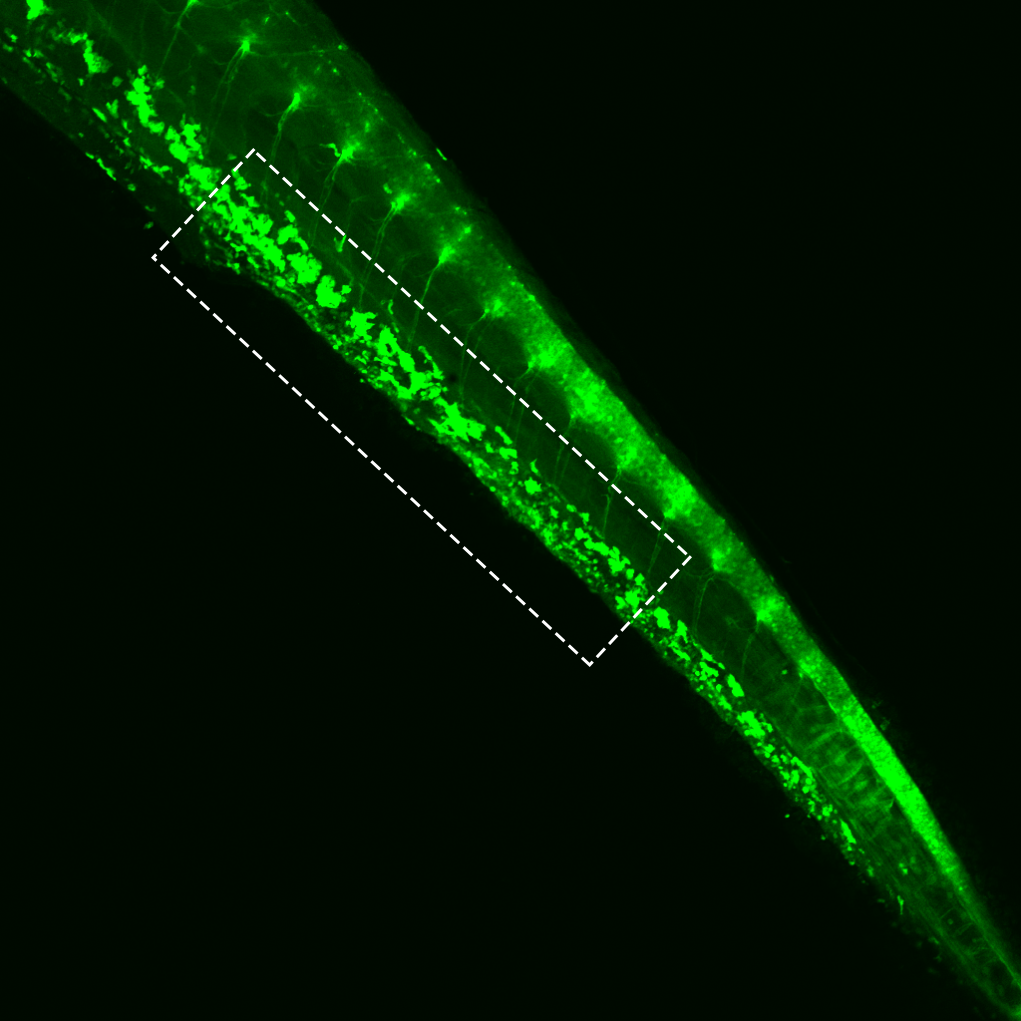

Supplement: Supplementary file 8 — Source data Fig. 3 [file 44318_2024_82_MOESM8_ESM.zip › Figure 3/3A/5dpf sibling GFP.tif]

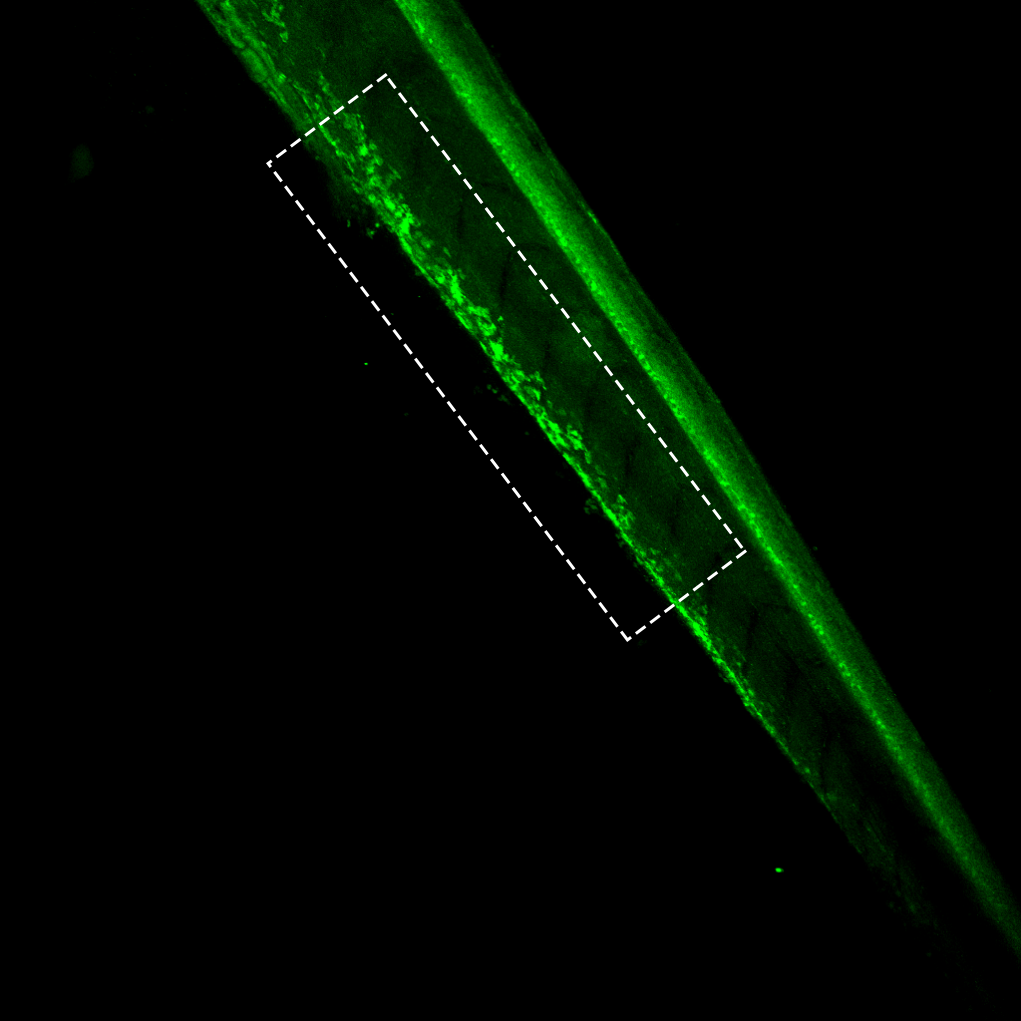

Supplement: Supplementary file 8 — Source data Fig. 3 [file 44318_2024_82_MOESM8_ESM.zip › Figure 3/3B/PCNA/5dpf mettl16-- GFP.tif]

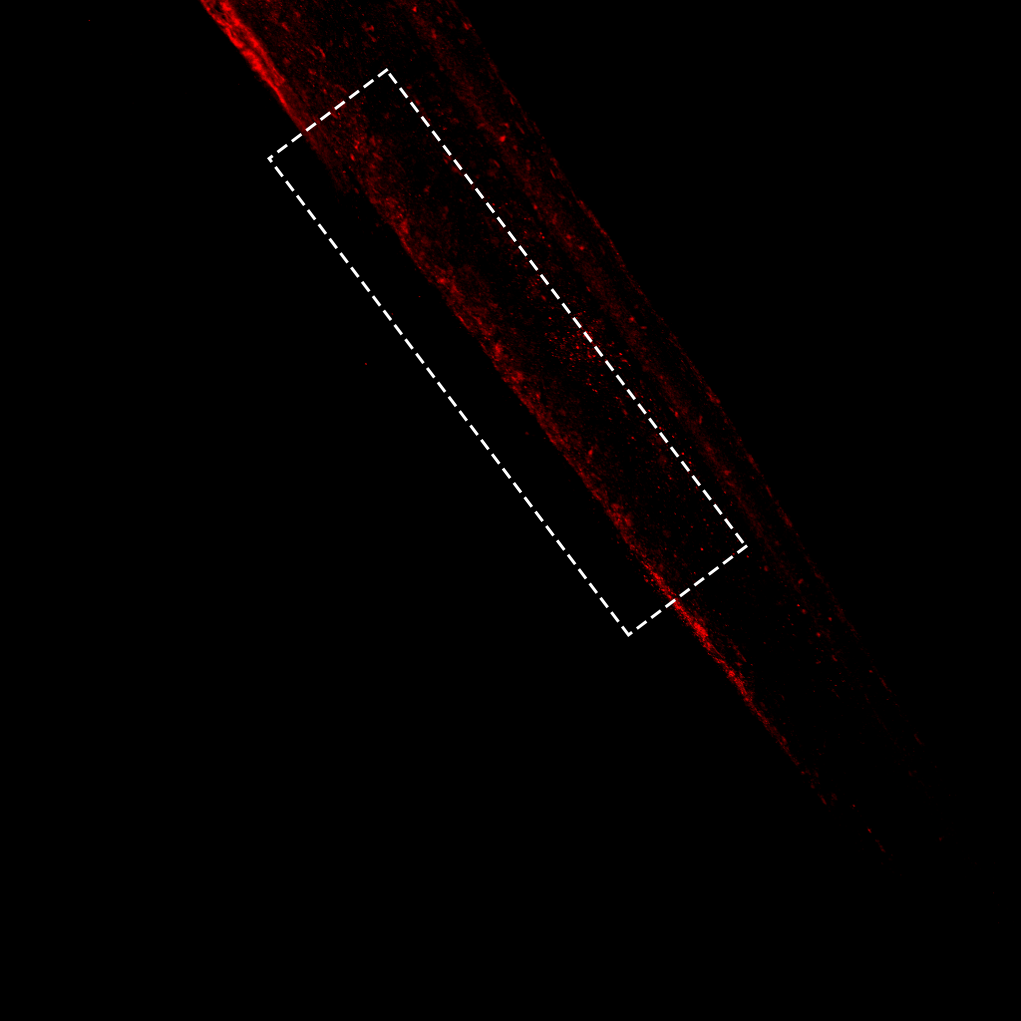

Supplement: Supplementary file 8 — Source data Fig. 3 [file 44318_2024_82_MOESM8_ESM.zip › Figure 3/3B/PCNA/5dpf mettl16-- PCNA.tif]

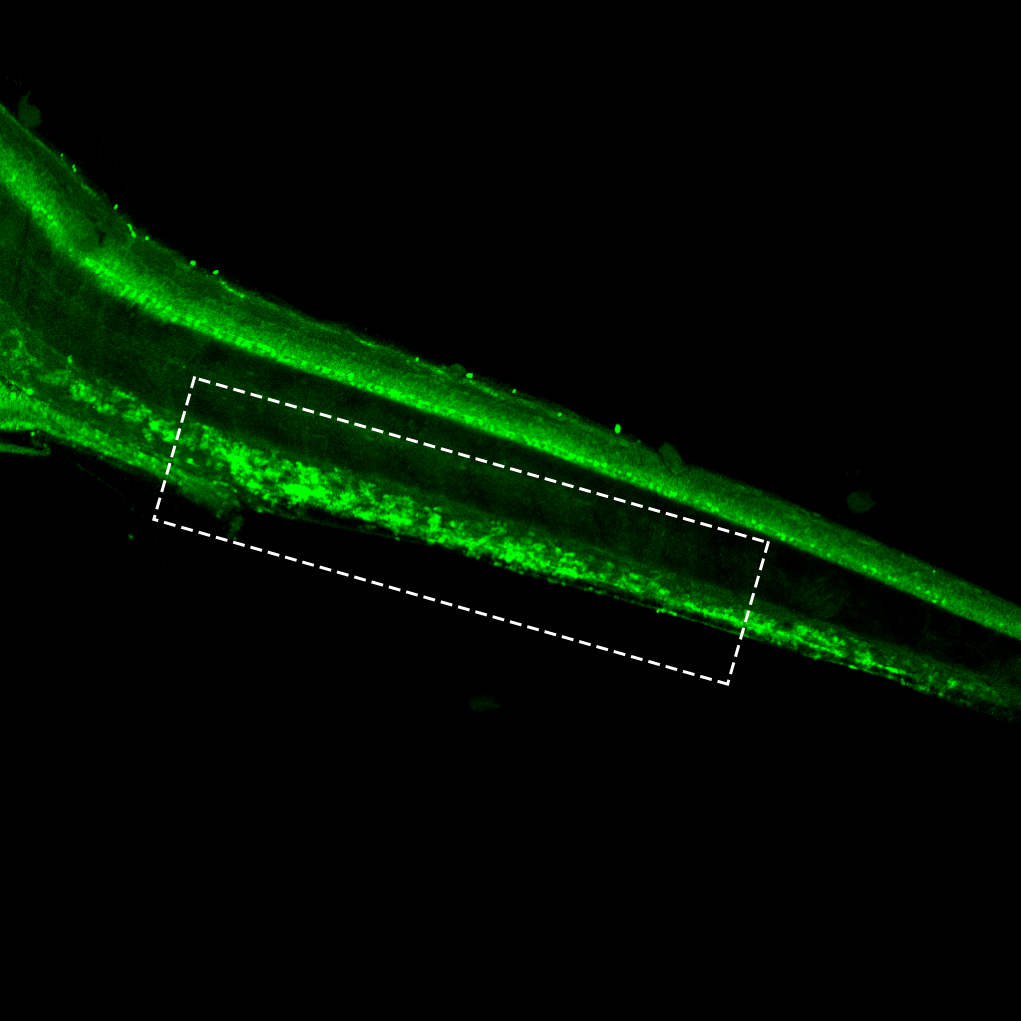

Supplement: Supplementary file 8 — Source data Fig. 3 [file 44318_2024_82_MOESM8_ESM.zip › Figure 3/3B/PCNA/5dpf sibling GFP.tif]

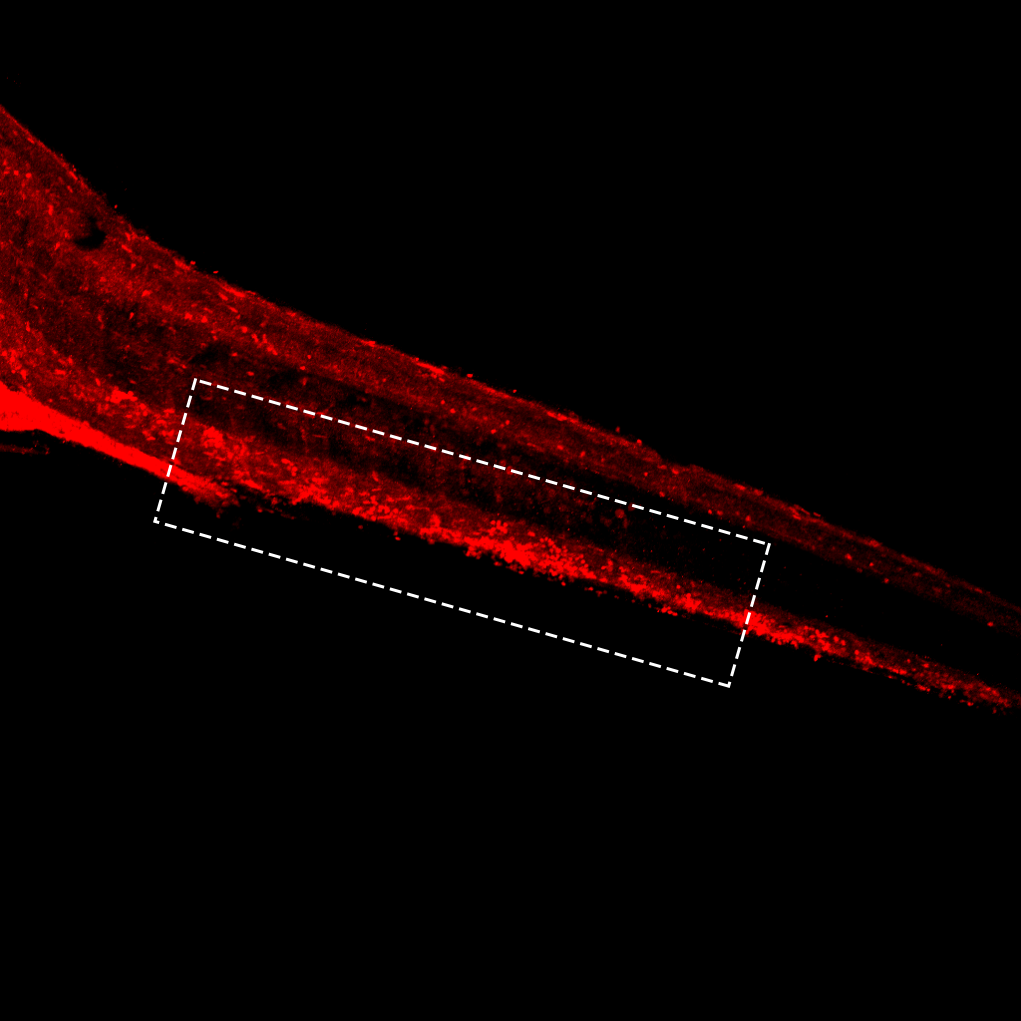

Supplement: Supplementary file 8 — Source data Fig. 3 [file 44318_2024_82_MOESM8_ESM.zip › Figure 3/3B/PCNA/5dpf sibling PCNA.tif]

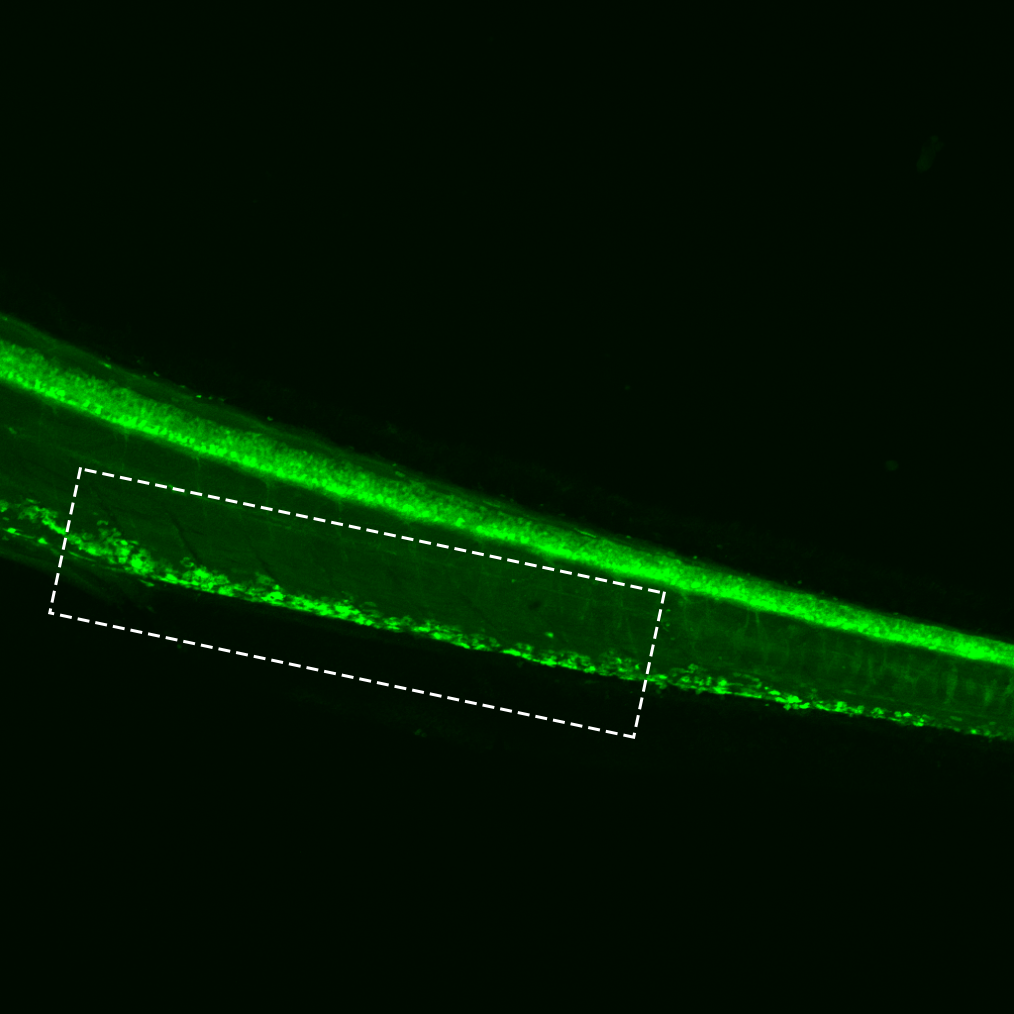

Supplement: Supplementary file 8 — Source data Fig. 3 [file 44318_2024_82_MOESM8_ESM.zip › Figure 3/3B/PH3/5dpf mettl16-- GFP.tif]

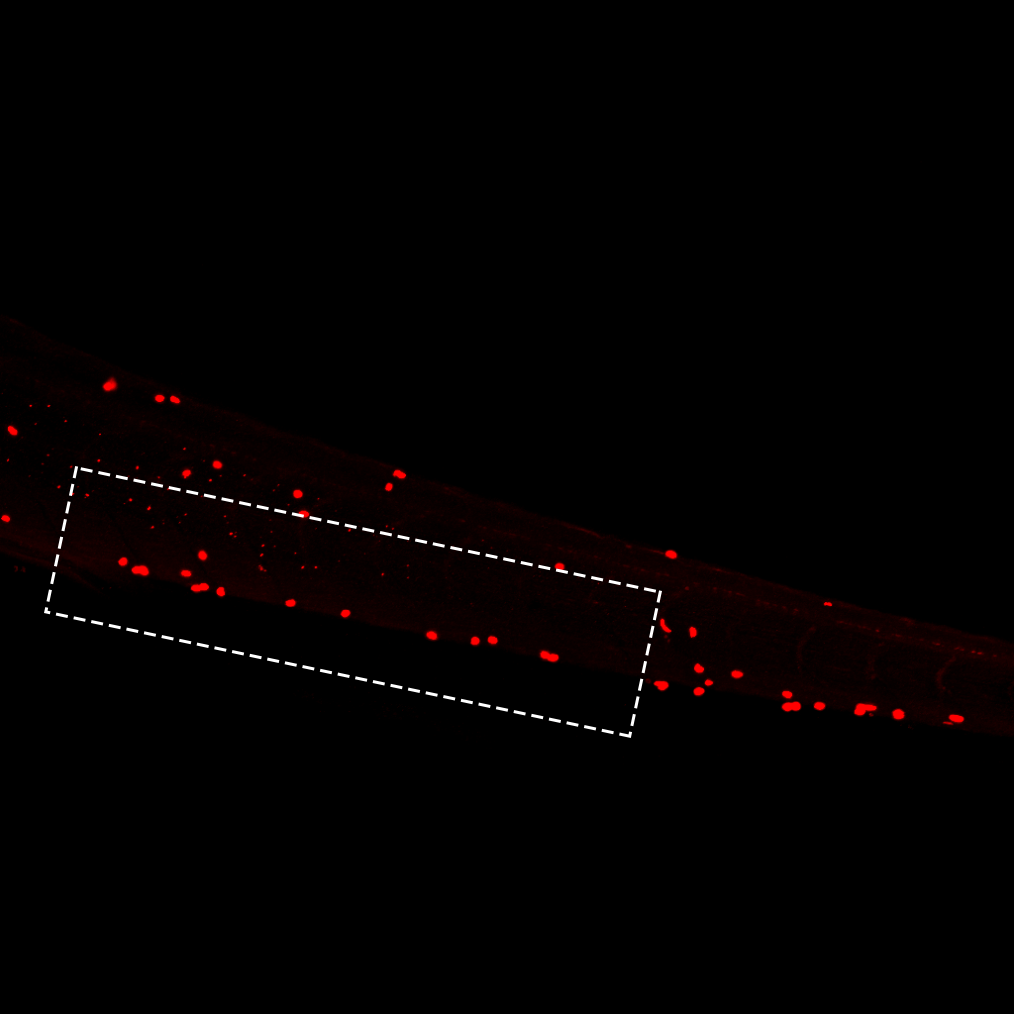

Supplement: Supplementary file 8 — Source data Fig. 3 [file 44318_2024_82_MOESM8_ESM.zip › Figure 3/3B/PH3/5dpf mettl16-- PH3.tif]

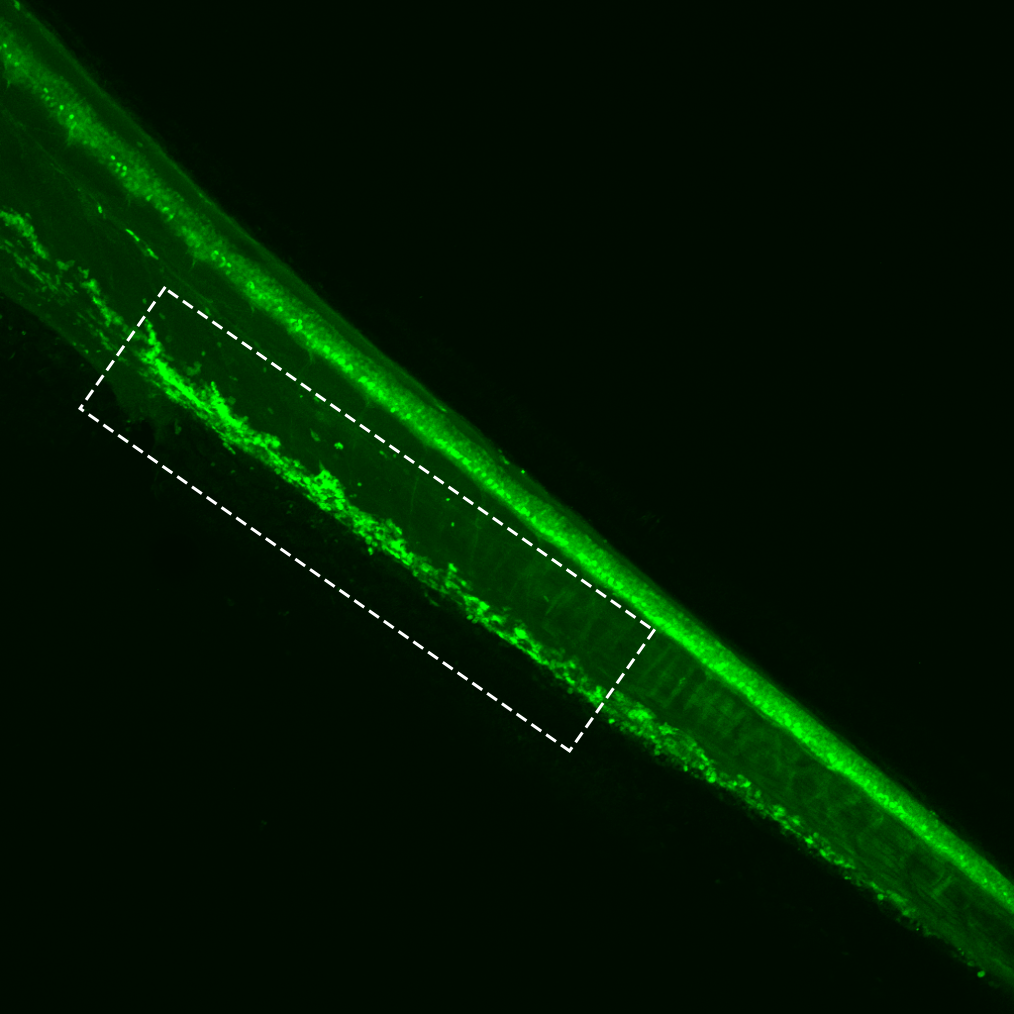

Supplement: Supplementary file 8 — Source data Fig. 3 [file 44318_2024_82_MOESM8_ESM.zip › Figure 3/3B/PH3/5dpf sibling GFP.tif]

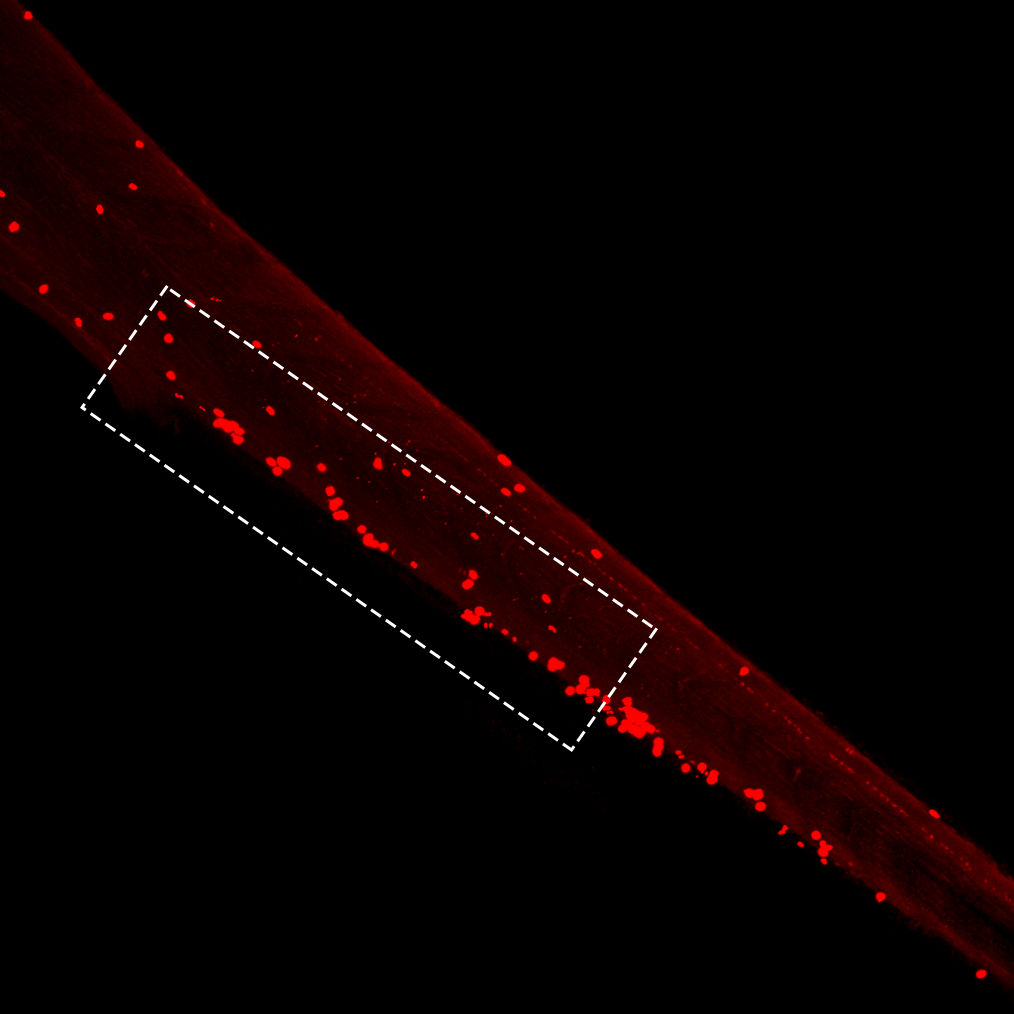

Supplement: Supplementary file 8 — Source data Fig. 3 [file 44318_2024_82_MOESM8_ESM.zip › Figure 3/3B/PH3/5dpf sibling PH3.tif]

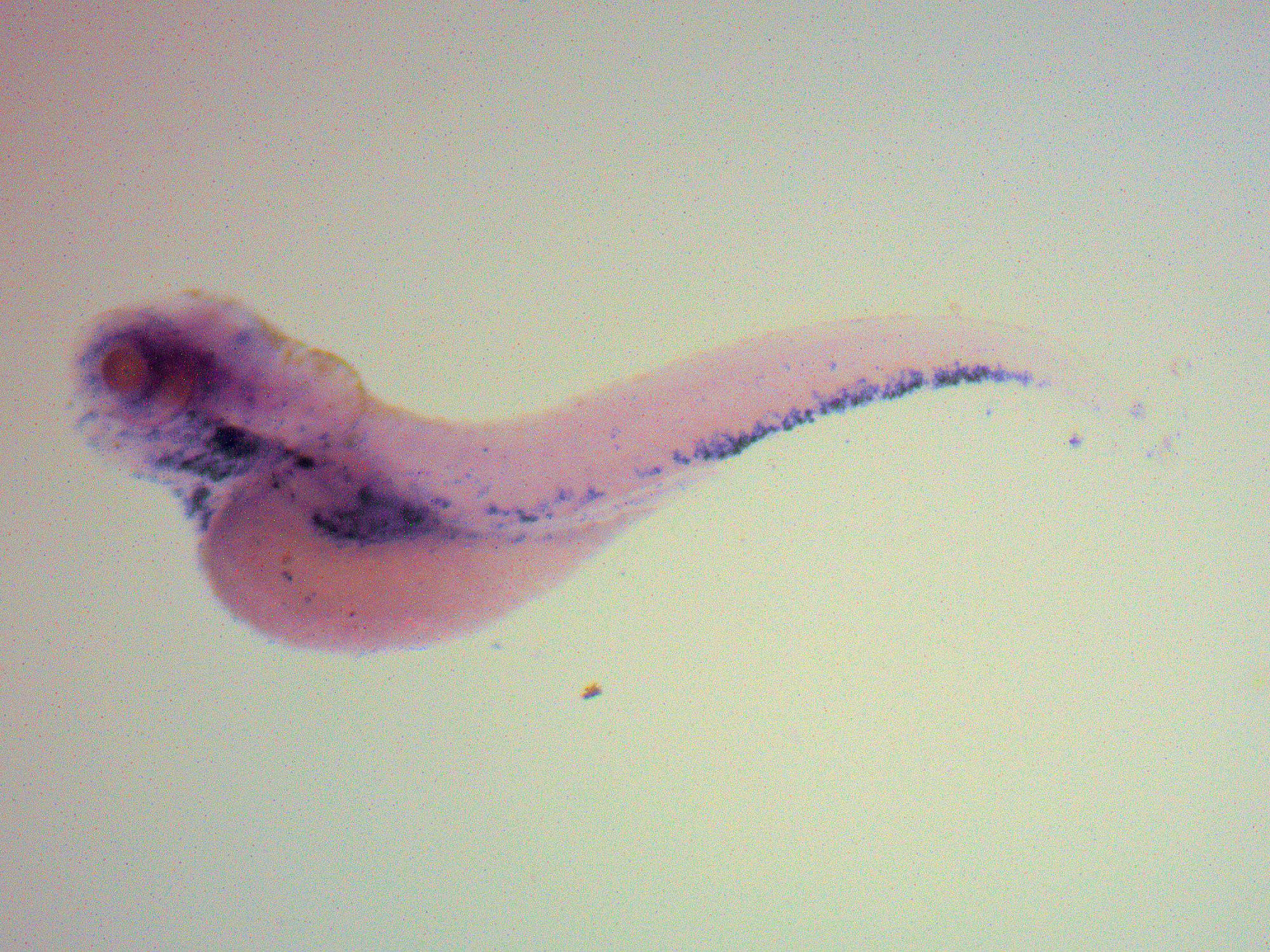

Supplement: Supplementary file 9 — Source data Fig. 4 [file 44318_2024_82_MOESM9_ESM.zip › Figure 4/4C/cmyb control+vector 4dpf.tif]

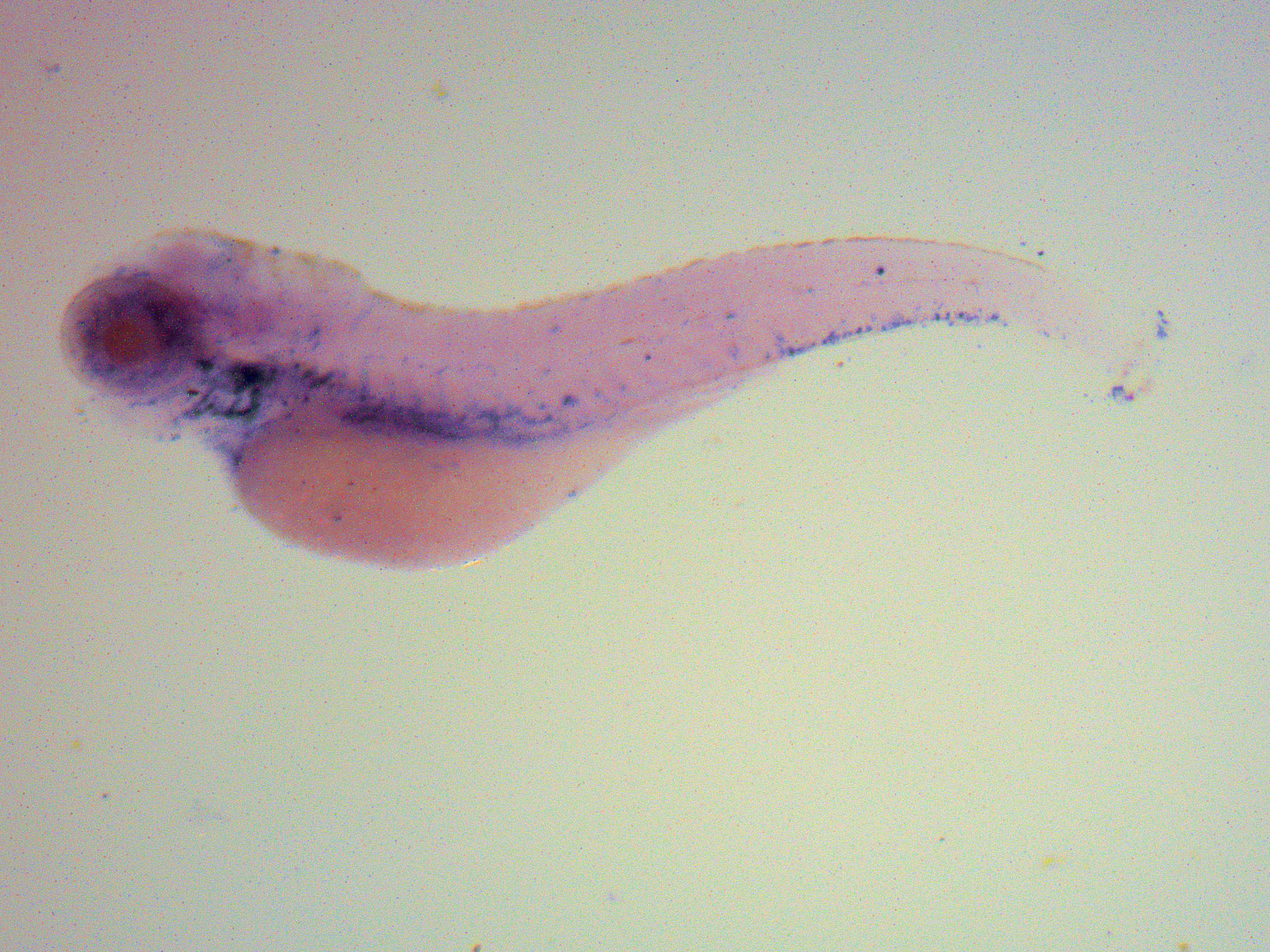

Supplement: Supplementary file 9 — Source data Fig. 4 [file 44318_2024_82_MOESM9_ESM.zip › Figure 4/4C/cmyb mo+mettl16-del NLS 4dpf.tif]

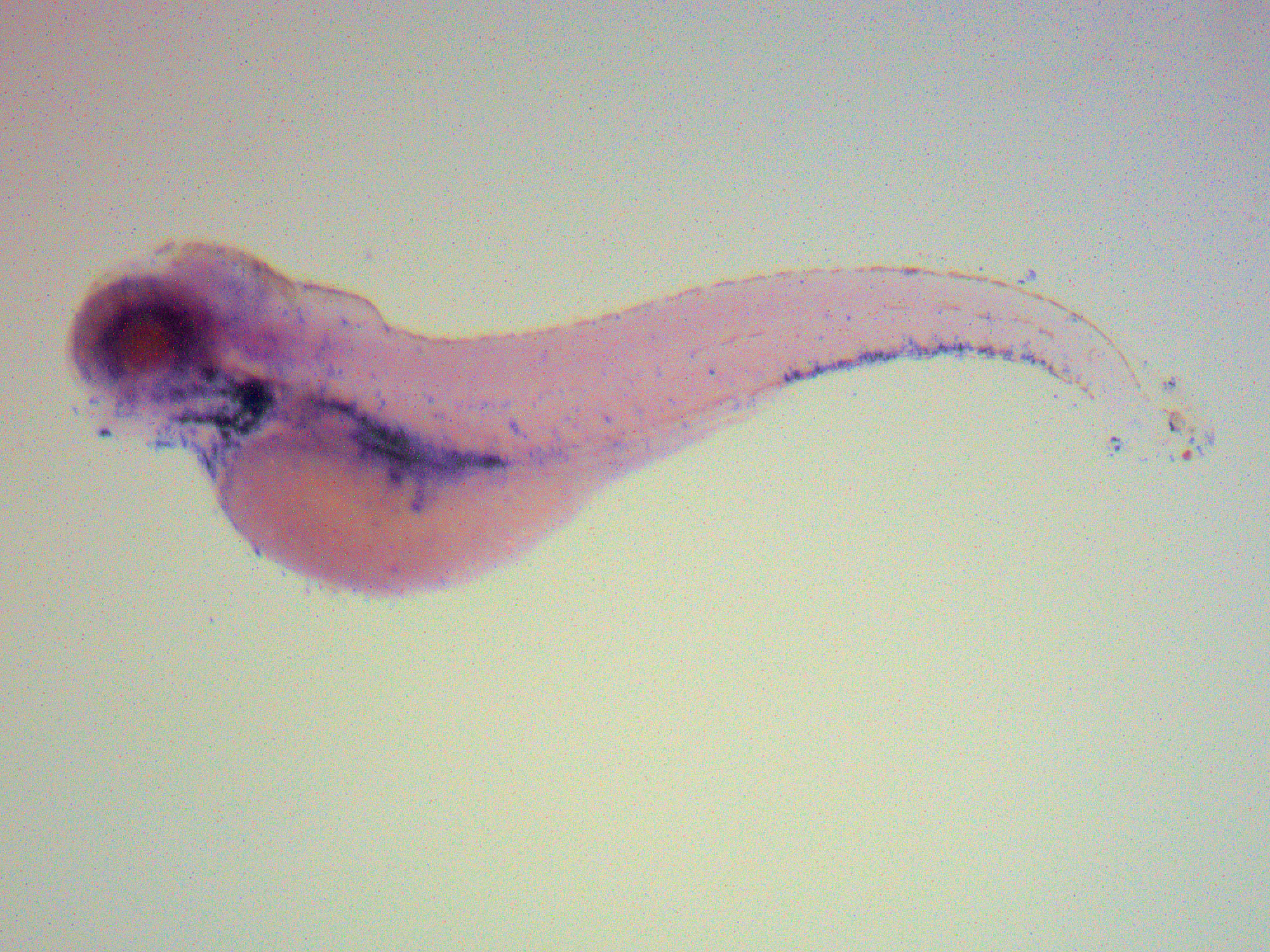

Supplement: Supplementary file 9 — Source data Fig. 4 [file 44318_2024_82_MOESM9_ESM.zip › Figure 4/4C/cmyb mo+Mettl16-PP180 181AA.tif]

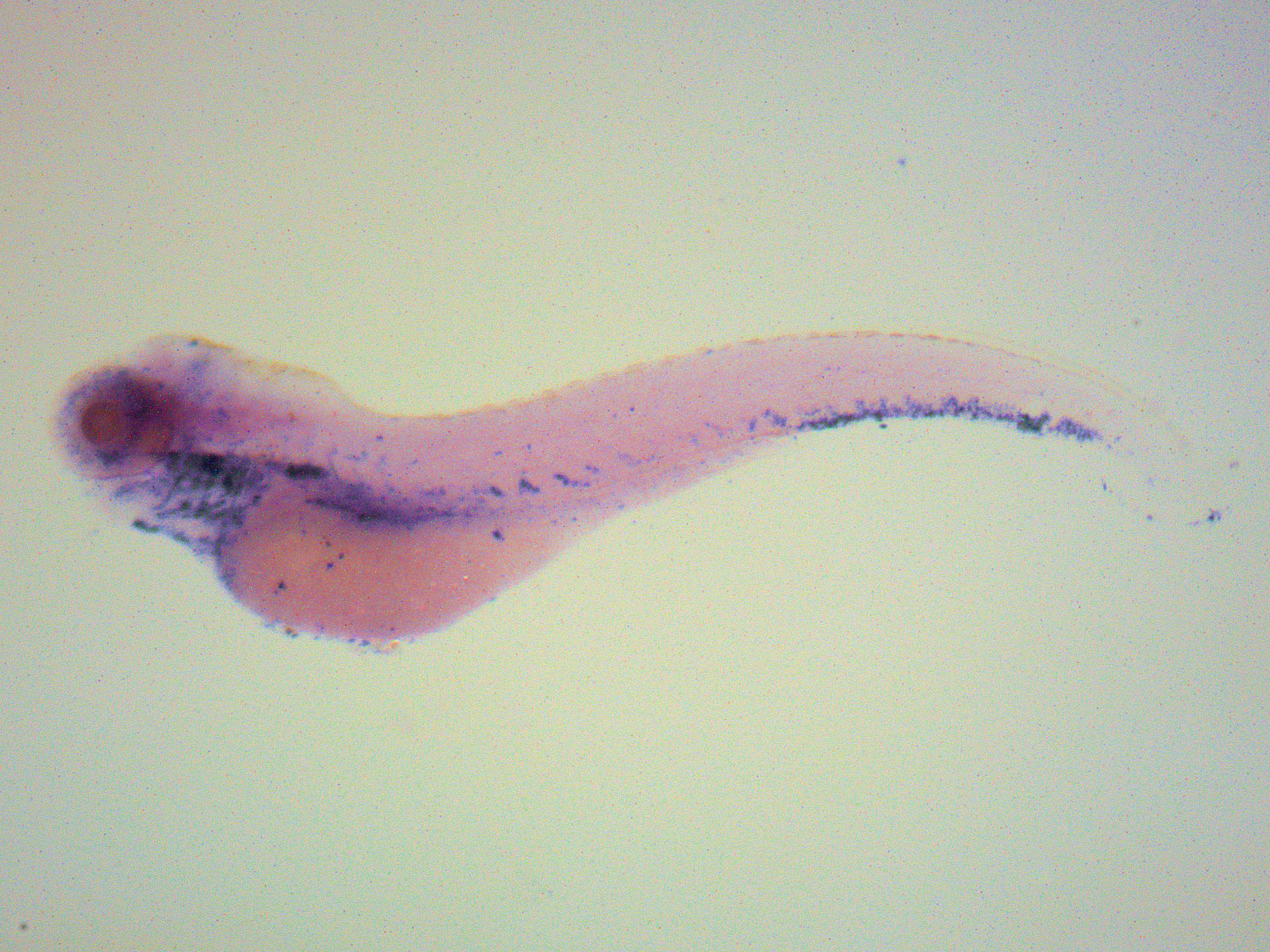

Supplement: Supplementary file 9 — Source data Fig. 4 [file 44318_2024_82_MOESM9_ESM.zip › Figure 4/4C/cmyb mo+mettl16-WT.tif]

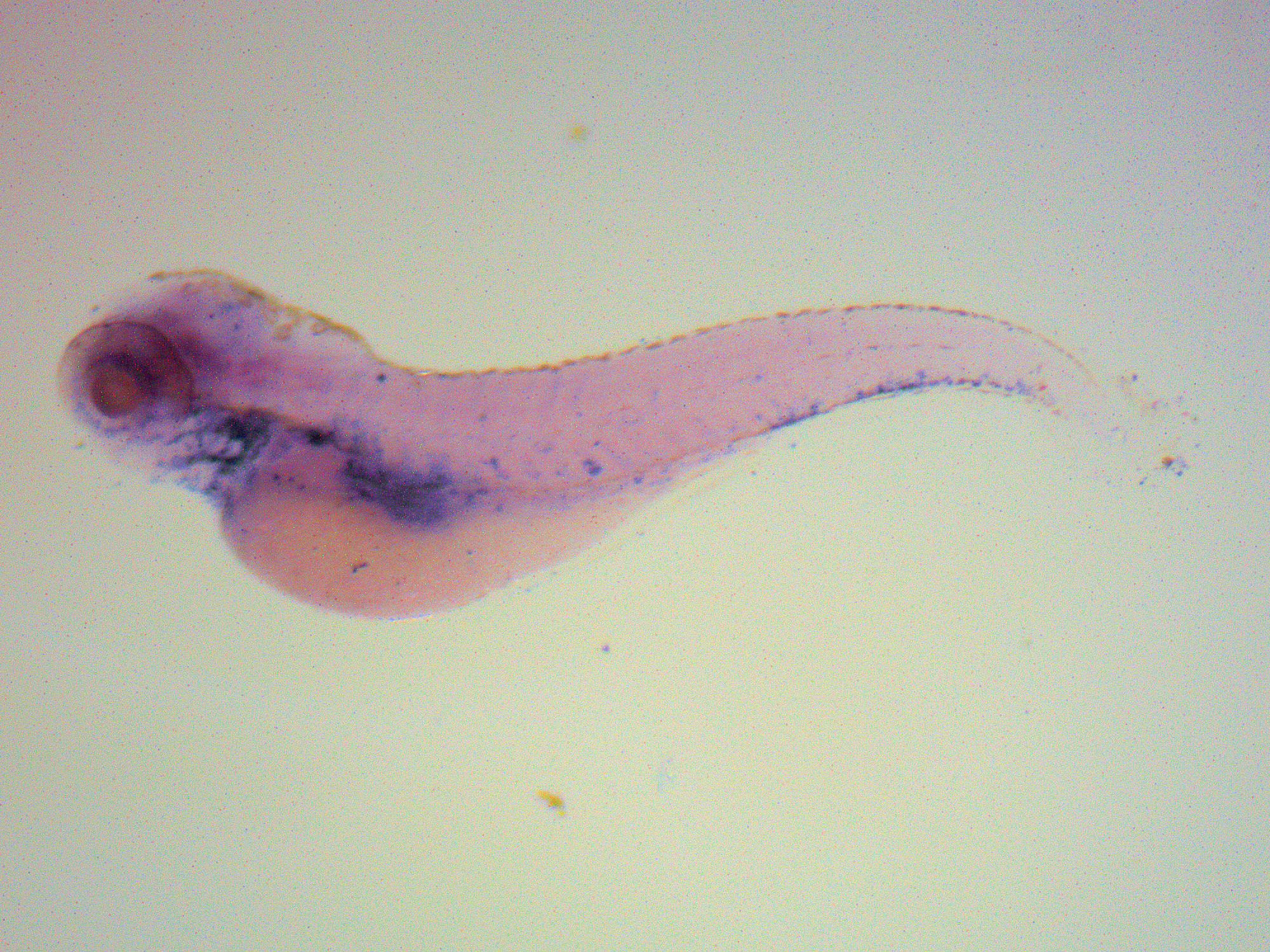

Supplement: Supplementary file 9 — Source data Fig. 4 [file 44318_2024_82_MOESM9_ESM.zip › Figure 4/4C/cmyb mo+vector.tif]

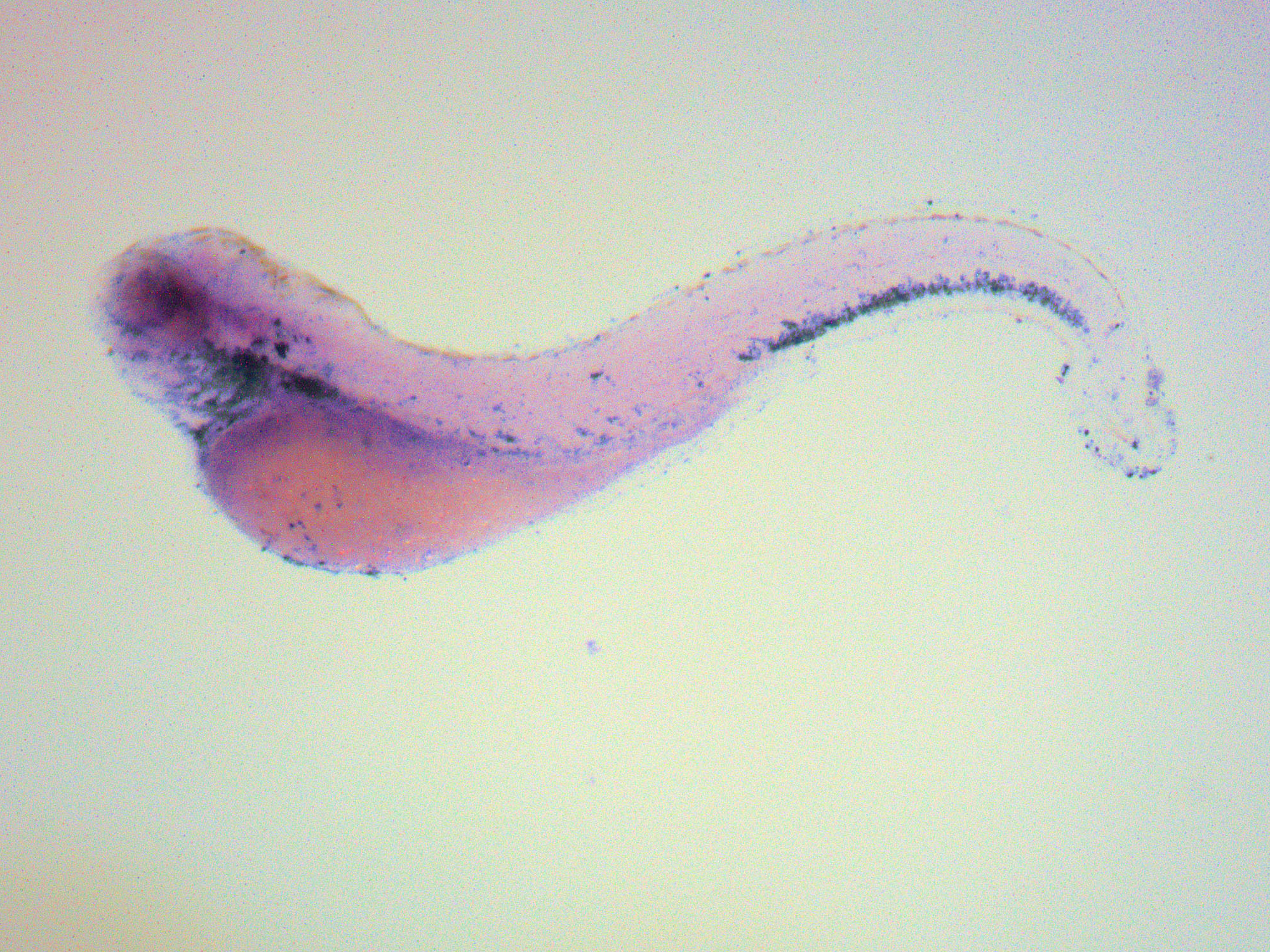

Supplement: Supplementary file 10 — Source data Fig. 5 [file 44318_2024_82_MOESM10_ESM.zip › Figure 5/5H/cmyb Control+vector.tif]

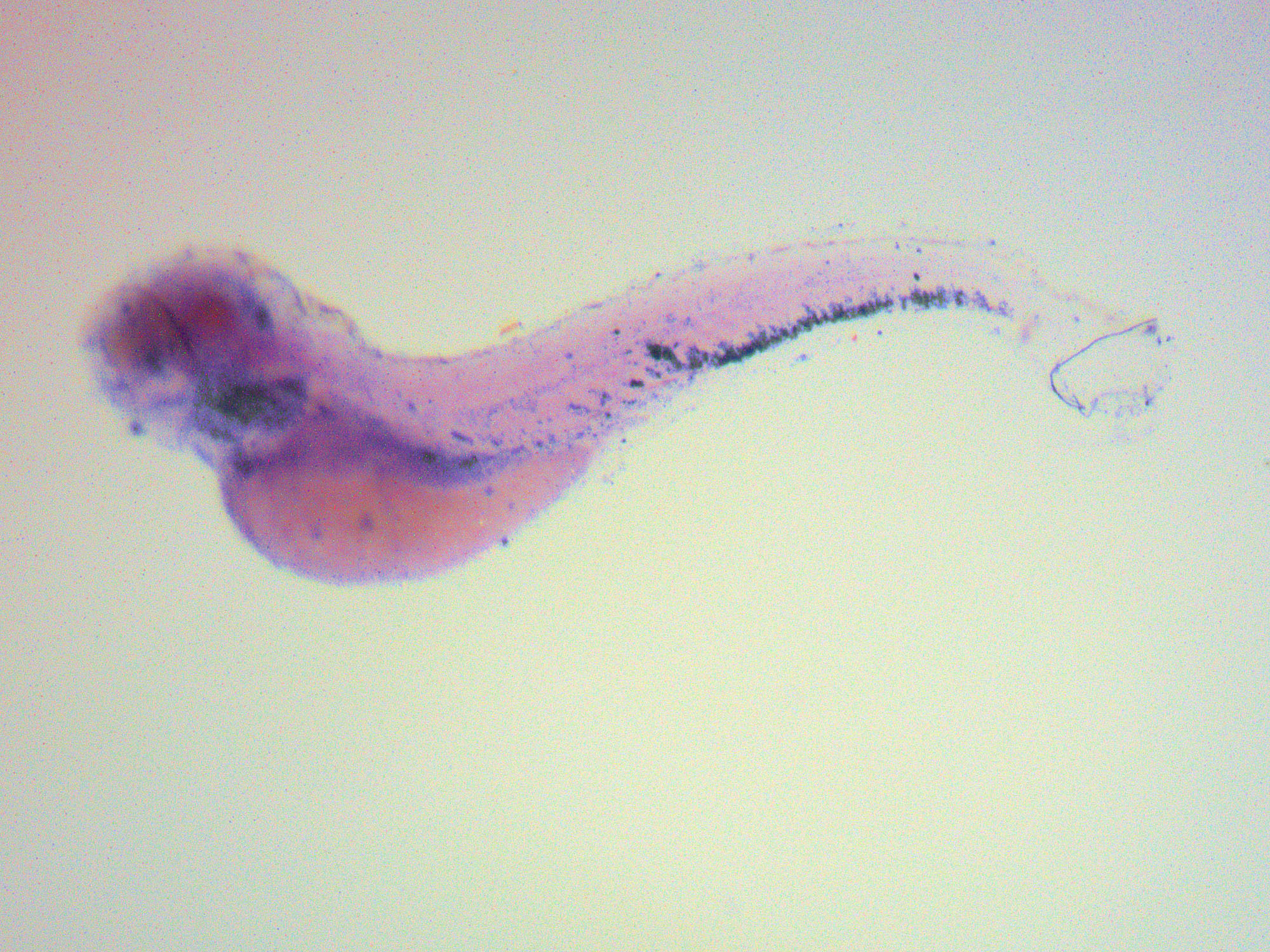

Supplement: Supplementary file 10 — Source data Fig. 5 [file 44318_2024_82_MOESM10_ESM.zip › Figure 5/5H/cmyb MO+mybl2b.tif]

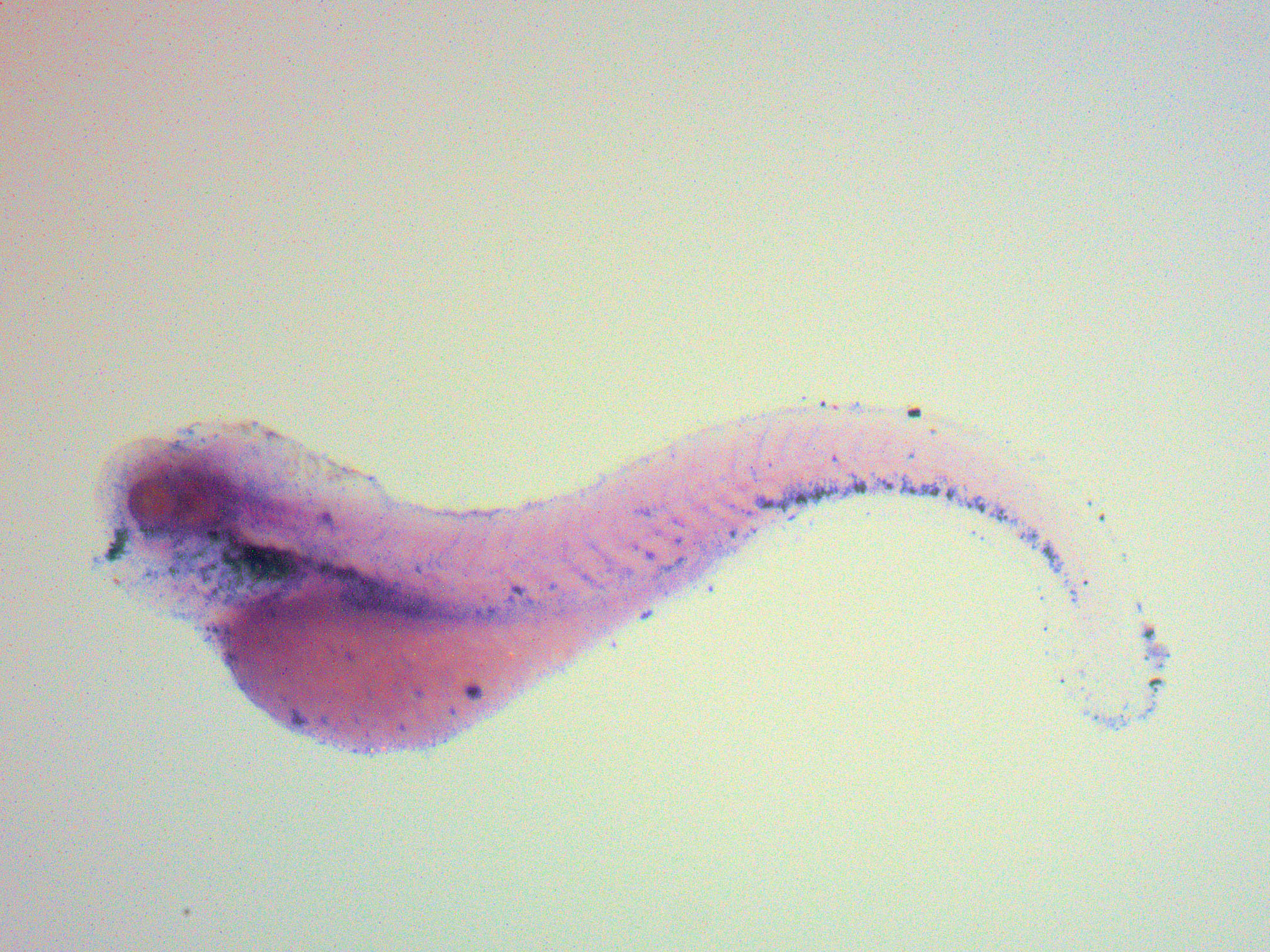

Supplement: Supplementary file 10 — Source data Fig. 5 [file 44318_2024_82_MOESM10_ESM.zip › Figure 5/5H/cmyb MO+vector.tif]

Source data for Fig 6F

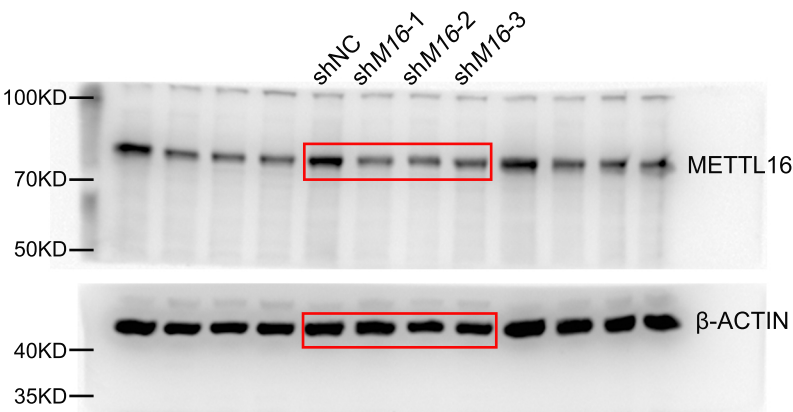

Supplement: Supplementary file 11 — Source data Fig. 6 [file 44318_2024_82_MOESM11_ESM.zip › Figure 6/6F/western blot METTL16.pdf]

Source data for Fig 6Q

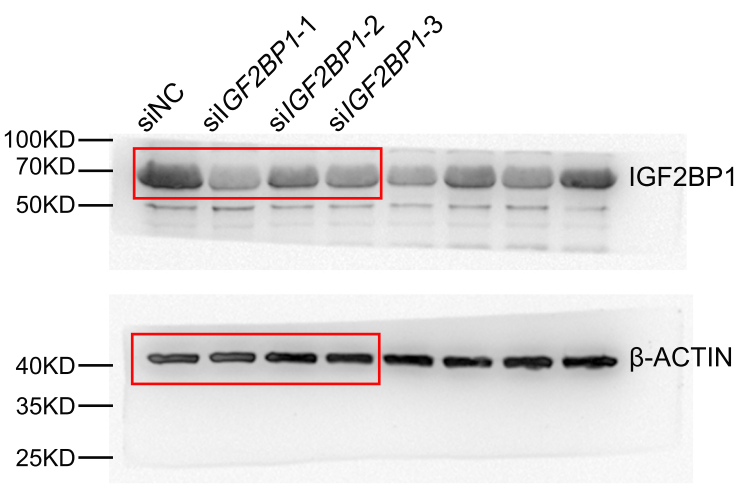

Supplement: Supplementary file 11 — Source data Fig. 6 [file 44318_2024_82_MOESM11_ESM.zip › Figure 6/6Q/western blot IGF2BP1.pdf]
