# Supplementary material for: A Mettl16/m6A/mybl2b/Igf2bp1 axis ensures cell cycle progression of embryonic hematopoietic stem and progenitor cells
Source: EMBO J. 2024 Apr 11;43(10):1990–2014. doi: 10.1038/s44318-024-00082-9 (PMC11099167; doi:10.1038/s44318-024-00082-9)
Supplement: Supplementary file 13 — Expanded View Figures [file 44318_2024_82_MOESM13_ESM.pdf]

Expanded View Figures

Figure EV1. Mettl16 governs HSPC maintenance in the CHT in a direct and cell-autonomous fashion.

(**A**) Expression of the HSPC markers *runx-1* and *cmyb* in the hemogenic endothelium of the ventral dorsal aorta in *mettl16* mutants by WISH at 28 hpf, 36 hpf, and 60 hpf, respectively. Numbers at the bottom right indicate the number of embryos with similar staining patterns among all embryos examined. $n = 3$ independent experiments. Scale bars, 100 µm. (**B**) Quantification of live imaging in Fig. 2J. '*n*' indicates the number of individuals analyzed for GFP^{+} cells in the CHT of siblings and *mettl16* mutants at 2 and 5 dpf, respectively. (**C**) WISH analysis showing the expression of arterial markers *dll4, efnb2a* and venous markers *dab2* in *mettl16* mutants at 30 hpf. Numbers at the bottom right indicate the number of embryos with similar staining patterns among all embryos examined. $n = 3$ independent experiments. Scale bars, 50 µm. (**D**) WISH analysis showing the expression of vascular markers *flk1* in *mettl16* mutants at 3 dpf (upper). Scale bars, 100 µm. Live imaging of artery and vein in the aorta-gonad-mesonephros (AGM) and CHT region within Tg (*flk1*: EGFP) background in siblings and *mettl16*$^{-/-}$ embryos at 30 hpf-4 dpf (lower). A artery, V vein. Scale bars, 40 um. Numbers at the bottom right indicate the number of embryos with similar staining patterns among all embryos examined. $n = 3$ independent experiments. (**E**) Dot plot showing the expression of *mettl16* in *kdrl*$^{+}$ cluster in Fig. 1E. (**F**) The spatiotemporal expression pattern of *mettl16* in the CHT analyzed from GSE120581. HSC hematopoietic stem cell, EC endothelial cell, HE hemogenic endothelium, NC non-endothelial and nonhematopoietic cells. (**G**) The expression of *desma* (a somite marker), *ifabp* (an intestine marker) and *huc* (a neuro marker) in sibling and *mettl16*$^{-/-}$ mutant embryos at 4 dpf. Numbers at the bottom right indicate the number of embryos with similar staining patterns among all embryos examined. $n = 3$ independent experiments. Scale bars, 100 µm. Data information: In (**B**), data were represented as mean ± SEM, ****adjusted $P < 0.0001$, n.s. non-significant, Student's *t*-test.

 © The Author(s)

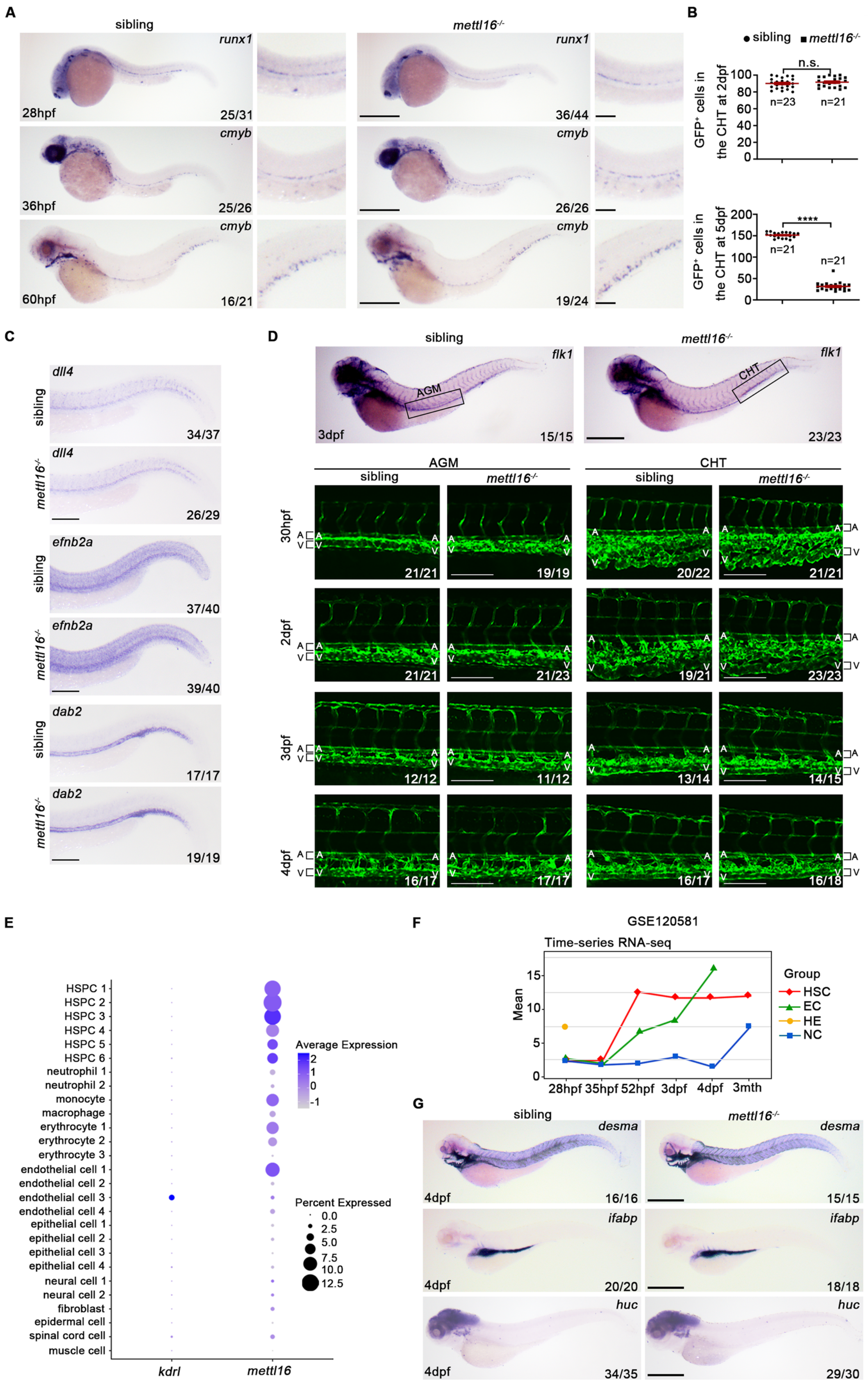
A
sibling
mettl16-/-
runx1
28hpf
25/31
36/44
cmyb
36hpf
25/26
26/26
60hpf
16/21
19/24
B
sibling
mettl16-/-
n.s.
GFP+ cells in the CHT at 2dpf
n=23
n=21
GFP+ cells in the CHT at 5dpf
****
C
dll4
34/37
26/29
efnb2a
37/40
39/40
dab2
17/17
19/19
D
flk1
AGM
CHT
3dpf
15/15
23/23
30hpf
2dpf
3dpf
4dpf
E
Average Expression
Percent Expressed
kdrl
mettl16
HSPC 1
HSPC 2
HSPC 3
HSPC 4
HSPC 5
HSPC 6
neutrophil 1
neutrophil 2
monocyte
macrophage
erythrocyte 1
erythrocyte 2
erythrocyte 3
endothelial cell 1
endothelial cell 2
endothelial cell 3
endothelial cell 4
epithelial cell 1
epithelial cell 2
epithelial cell 3
epithelial cell 4
neural cell 1
neural cell 2
fibroblast
epidermal cell
spinal cord cell
muscle cell
F
GSE120581
Time-series RNA-seq
Mean
Group
HSC
EC
HE
NC
28hpf 35hpf 52hpf 3dpf 4dpf 3mth
G
desma
4dpf
16/16
15/15
ifabp
20/20
18/18
huc
34/35
29/30

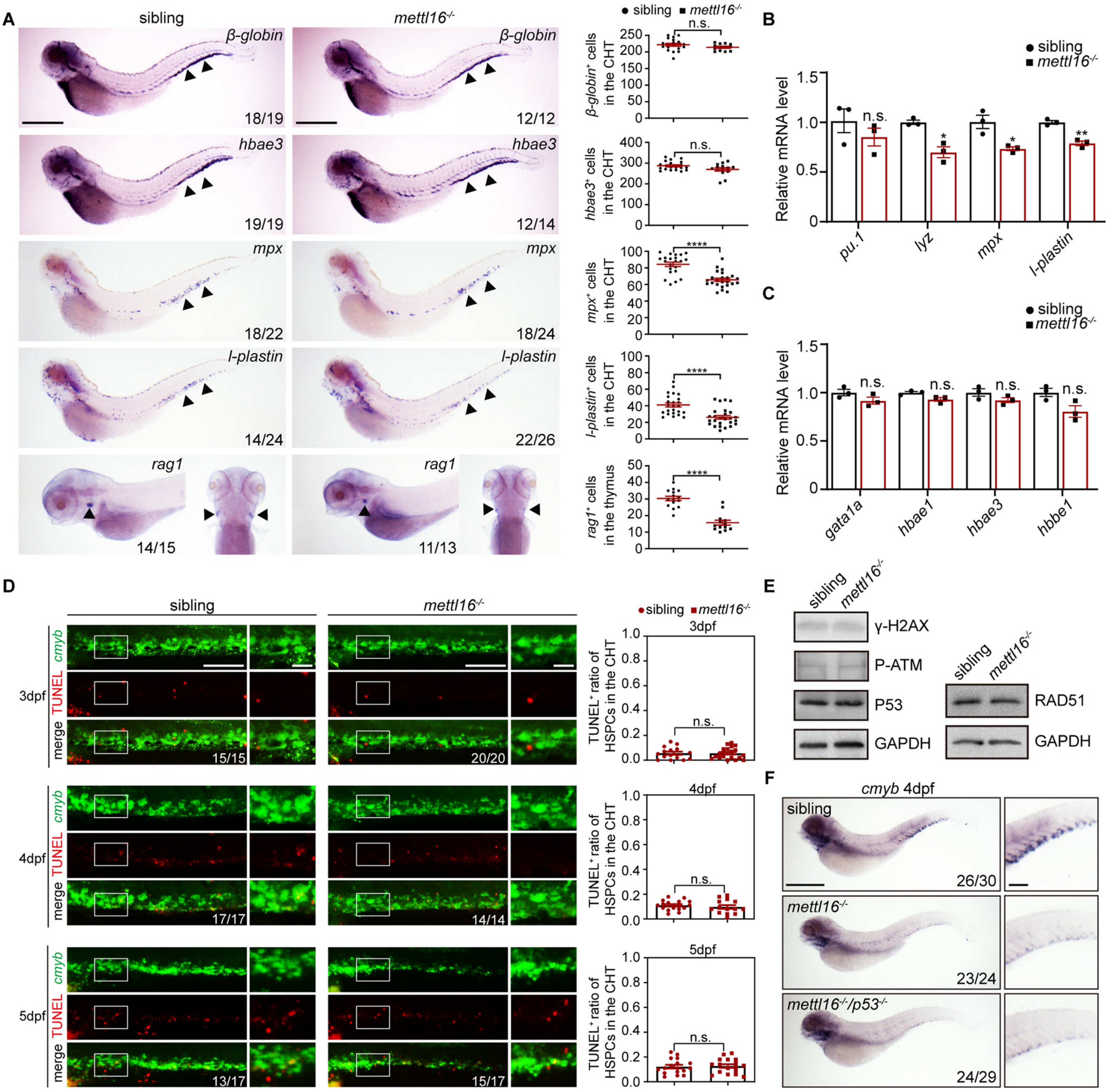

Figure EV2. Loss of Mettl16 does not cause excessive differentiation or apoptosis of HSPCs.

(**A**) The expression of erythroid cell markers *b-globin* and *hbae3*, myeloid cell markers *mpx* and *l-plastin* at 3 dpf, as well as lymphocytes marker *rag1* at 4 dpf in siblings and *mettl16* mutants by WISH. Numbers at the bottom right indicate the number of embryos with similar staining patterns among all embryos examined. $n = 3$ independent experiments. The black arrowheads indicate differentiated blood cells. Scale bars, 100 μm. (**B, C**) qPCR showing the expression of myeloid cell markers (**B**) and erythroid cell markers (**C**) in siblings and *mettl16* mutants at 3 dpf. $n \geq 15$ per group, performed with three biological replicates. (**D**) Double immunostaining of *cmyb*: EGFP and TUNEL showing the number of apoptotic HSPCs in siblings and *mettl16* mutants from 3 to 5 dpf. Numbers at the bottom right indicate the number of embryos with similar staining patterns among all embryos examined. $n = 3$ independent experiments. Scale bars, 40 μm. (**E**) Western blot showing the protein expression level of γ-H2AX, P-ATM, P53, and RAD51 in siblings and *mettl16* mutants at 4 dpf. $n \geq 20$ per group, performed with three biological replicates. (**F**) Effect of restoration of P53 deletion on the expression of HSPC marker *cmyb* in *mettl16*$^{-/-}$ embryos at 4 dpf. Numbers at the bottom right indicate the number of embryos with similar staining patterns among all embryos examined. $n = 3$ independent experiments. Scale bars, 100 μm. Data information: In (**A–D**), data were represented as mean ± SEM, *adjusted $P < 0.05$, **adjusted $P < 0.01$, **** adjusted $P < 0.0001$, n.s. non-significant, Student's *t*-test.

 © The Author(s)

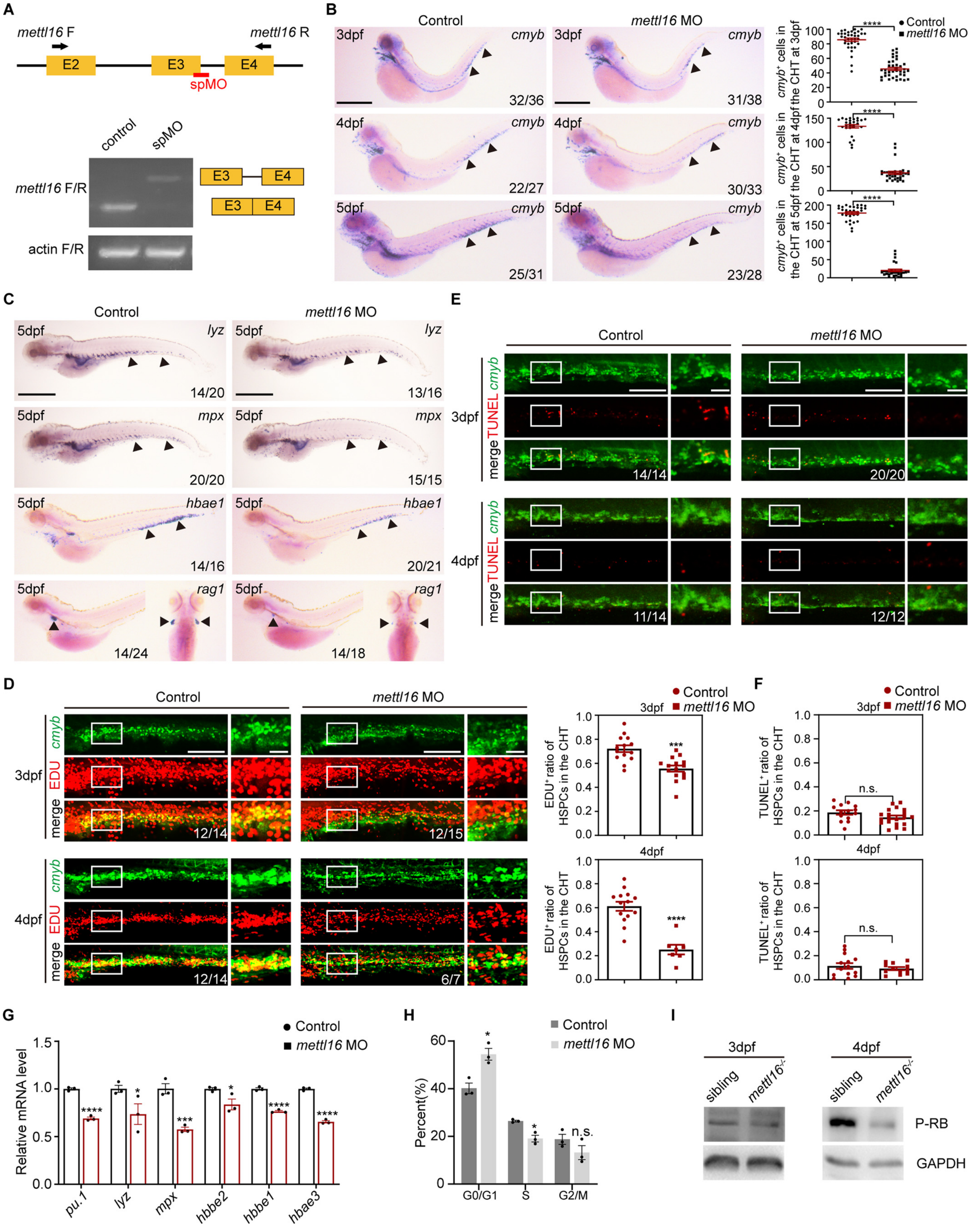
A
mettl16 F
mettl16 R
E2
E3
E4
spMO
control
spMO
mettl16 F/R
actin F/R
B
Control
mettl16 MO
3dpf
4dpf
5dpf
cmyb
32/36
31/38
22/27
30/33
25/31
23/28
cmyb+ cells in the CHT at 3dpf
cmyb+ cells in the CHT at 4dpf
cmyb+ cells in the CHT at 5dpf
Control
mettl16 MO
****
C
lyz
mpx
hbae1
rag1
14/20
13/16
20/20
15/15
14/16
20/21
14/24
14/18
E
3dpf
4dpf
cmyb
TUNEL
merge
14/14
20/20
11/14
12/12
D
EDU
12/14
12/15
6/7
EDU+ ratio of HSPCs in the CHT
***
F
TUNEL+ ratio of HSPCs in the CHT
n.s.
G
Relative mRNA level
pu.1
lyz
mpx
hbbe2
hbbe1
hbae3
H
Percent(%)
G0/G1
S
G2/M
I
sibling
mettl16-/-
P-RB
GAPDH

◀ **Figure EV3. *mettl16* morpholino reproduces the *mettl16* knockout effect on HSPCs.**

(**A**) The *mettl16* splice MO was designed to target the boundary of exon3 and intron 3 (red line marks the target region). The primers were used to amplify the exon region for PCR validation of this MO. (**B**) WISH assay showing the number of HSPCs in the CHT of *mettl16* morphants from 3 dpf to 5 dpf. Numbers at the bottom right indicate the number of embryos with similar staining patterns among all embryos examined. $n = 3$ independent experiments. The black arrowheads indicate *cmyb*$^{+}$ cells in the CHT. Scale bars, 100 µm. (**C**) Expression of definitive hematopoiesis markers-*lyz* (myeloid), *mpx* (myeloid), *hbae1* (erythroid), and *rag1* (lymphoid) in *mettl16* morphants compared with control at 5 dpf. Numbers at the bottom right indicate the number of embryos with similar staining patterns among all embryos examined. $n = 3$ independent experiments. The black arrowheads indicate differentiated blood cells. Scale bars, 100 µm. (**D**) Double immunostaining of *cmyb*: EGFP and EDU showing the proliferation of HSPCs in the CHT of morphants at 3 dpf and 4 dpf. Numbers at the bottom right indicate the number of embryos with similar staining patterns among all embryos examined. $n = 3$ independent experiments. Scale bars, 40 µm. (**E**, **F**) Double immunostaining of *cmyb*: EGFP and TUNEL showing the number of apoptotic HSPCs at 3-4 dpf. Numbers at the bottom right indicate the number of embryos with similar staining patterns among all embryos examined. $n = 3$ independent experiments. Scale bars, 40 µm. (**G**) qPCR showing the expression of myeloid cell markers (*pu.1*, *lyz*, and *mpx*) and erythroid cell markers (*hbbe2*, *hbbe1*, and *hbae3*) in *mettl16* morphants compared with control at 4 dpf. $n \geq 15$ per group, performed with three biological replicates. (**H**) Flow analysis showing the cell cycle of HSPCs of *mettl16*-deficient zebrafish at 5 dpf. $n \geq 200$ per group, performed with three biological replicates. (**I**) The protein expression level of P-RB in *mettl16*$^{-/-}$ embryos compared with siblings at 3–4 dpf. $n \geq 20$ per group, performed with three biological replicates. Data information: In (**B**, **D**, **F**, **G**, **H**), data were represented as mean ± SEM, *adjusted $P < 0.05$, ***adjusted $P < 0.001$, ****adjusted $P < 0.0001$, n.s. non-significant, Student's *t*-test.

 © The Author(s)

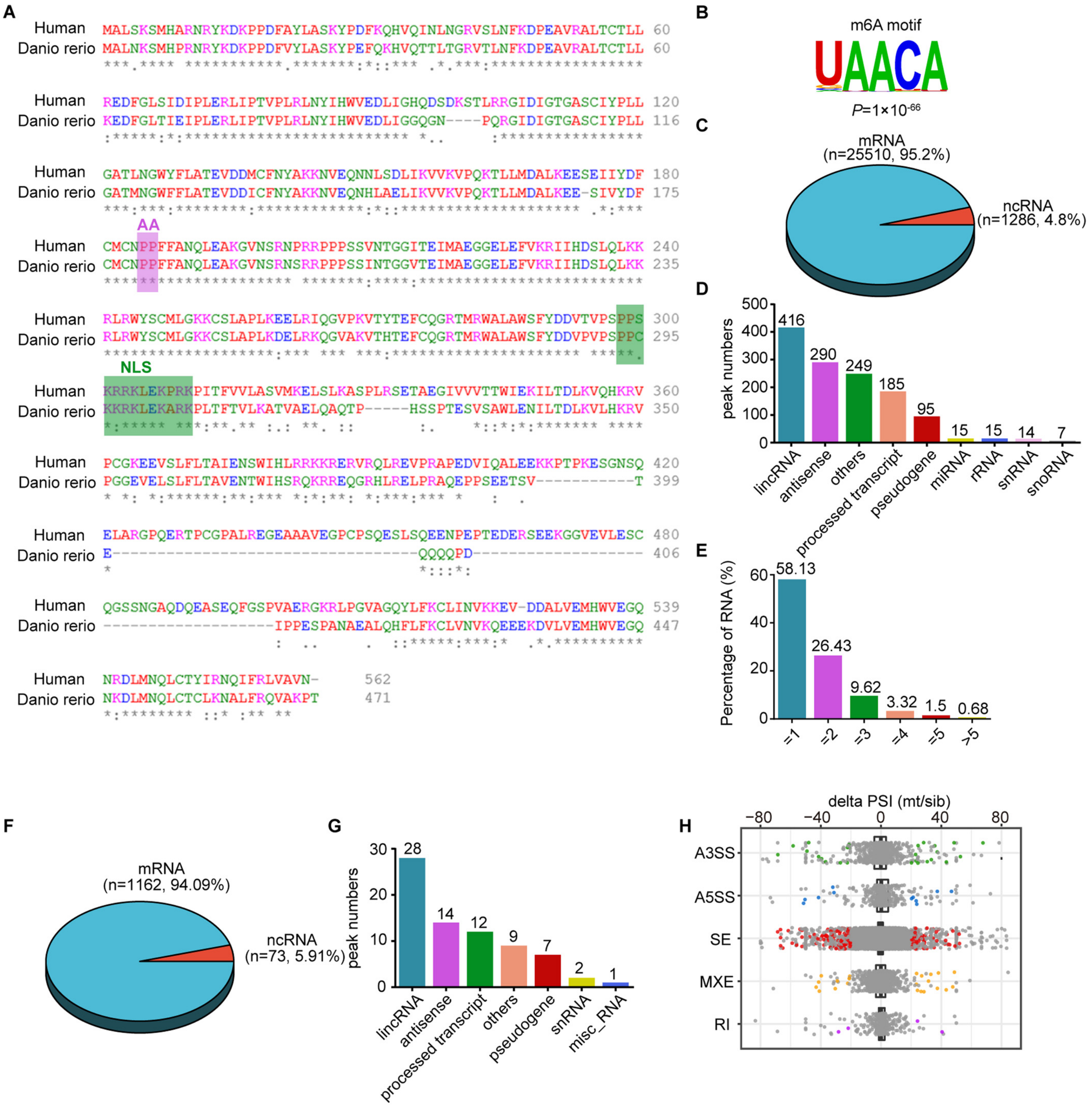

Figure EV4. Disruption of Mettl16 invokes the alteration of m⁶A methylome and transcriptome.

(**A**) Protein sequence alignment of METTL16 proteins between zebrafish and humans. The pink rectangle indicates the site of PP180/181AA, and the green rectangle indicates the site of predicted nuclear localization signal (NLS). (**B**) Sequence motif identified within m^6A peaks by HOMER database in siblings. (**C**) Percentage of mRNAs and non-coding RNAs containing m^6A peaks in siblings. (**D**) Bar plots showing the number of representative non-coding RNAs containing m^6A peaks in siblings. (**E**) Percentage of m^6A-methylated mRNAs with different numbers of m^6A peaks in siblings. (**F**) Percentage of mRNAs and non-coding RNAs containing hypomethylated m^6A peaks in *mettl16* mutants. (**G**) Bar plots showing the number of representative non-coding RNAs containing hypomethylated m^6A peaks in *mettl16* mutants. (**H**) Categories of differentially spliced genes based on the changed PSI value in the *mettl16* mutants (mt). The percent-spliced-in index (PSI) indicates the efficiency of splicing a specific exon into the transcript population of a gene. A3SS alternative 3′ splice site, A5SS alternative 5′ splice site, SE skipped exon, RI retained intron, MXE mutually exclusive exon.

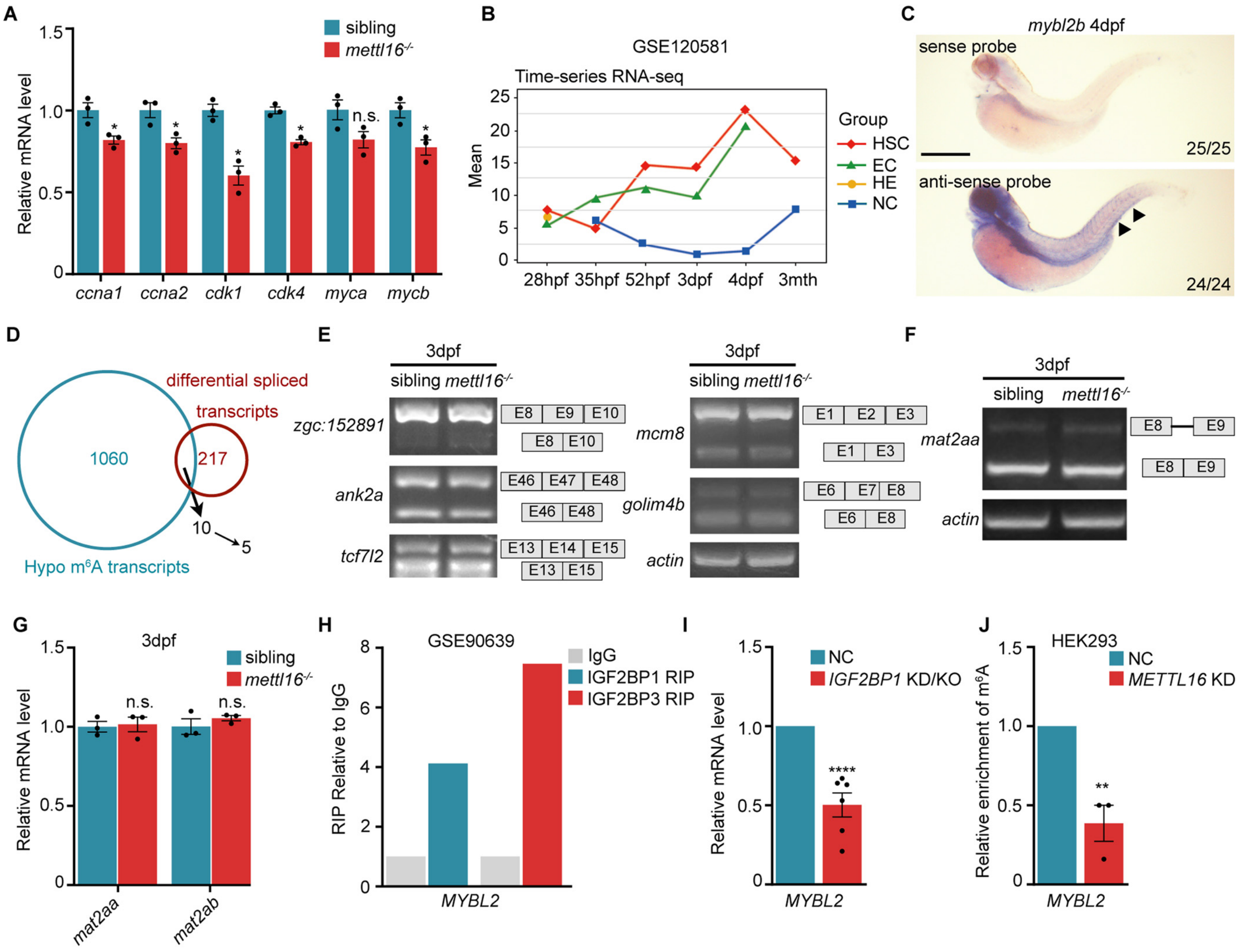

Figure EV5. METTL16 regulates the expression of *MYBL2* in an m⁶A-dependent manner mediated by IGF2BP1.

(**A**) qPCR analysis showing the mRNA expression level of the downstream targets associated with G1/S arrest of *mybl2b* in *mettl16*$^{-/-}$ embryos at 3 dpf. $n \geq 15$ per group, performed with three biological replicates. (**B**) The spatiotemporal expression pattern of *mybl2b* in the CHT generated from GSE120581. HSC hematopoietic stem cell, EC endothelial cell, HE hemogenic endothelium, NC non-endothelial and nonhematopoietic cells. (**C**) WISH analysis of *mybl2b* spatiotemporal expression in siblings at 4 dpf. Numbers at the bottom right indicate the number of embryos with similar staining patterns among all embryos examined. $n = 3$ independent experiments. The black arrowheads indicate *mybl2b*$^{+}$ cells in the CHT. Scale bars, 100 μm. (**D**) Venn diagram showing the overlap between differentially spliced genes ($P < 0.05$, |delta PSI| ≥0.2) and hypomethylated transcripts in *mettl16* mutants compared with siblings. Statistical analysis was performed by likelihood-ratio test. (**E**) Semi-qPCR analysis showing the splicing of G0/G1 arrest-related genes with differential splicing and hypomethylated m⁶A peaks in *mettl16* mutants. $n \geq 15$ per group, performed with three biological replicates. (**F**) Semi-qPCR analysis showing the splicing of *mat2aa* in *mettl16* mutants at 3 dpf. $n \geq 15$ per group, performed with three biological replicates. (**G**) qPCR analysis showing the mature mRNA expression level of *mat2aa* and *mat2ab* in *mettl16* mutants at 3 dpf. $n \geq 15$ per group, performed with three biological replicates. (**H**) IGF2BP1 and IGF2BP3 can bind *MYBL2* mRNA directly in HEK293 cells by RIP-seq (GSE90639). (**I**) RNA-seq analysis showed the mRNA expression level of *MYBL2* in *IGF2BP1* knockdown or knockout cells. Each data point in the figure corresponds to independent data sourced from GSE146546, GSE146803, GSE161087, GSE158258, GSE133097, and GSE161086, respectively. (**J**) m⁶A-seq analysis showed the m⁶A level of *MYBL2* upon *METTL16* knockdown in HEK293 cells. Each data point in the figure corresponds to three biological replicates from GSE90914. Data information: In (**A**, **G**, **I**, **J**), data were represented as mean ± SEM, *adjusted $P < 0.05$, **adjusted $P < 0.01$, **** adjusted $P < 0.0001$, n.s. non-significant, Student's *t*-test.

 © The Author(s)

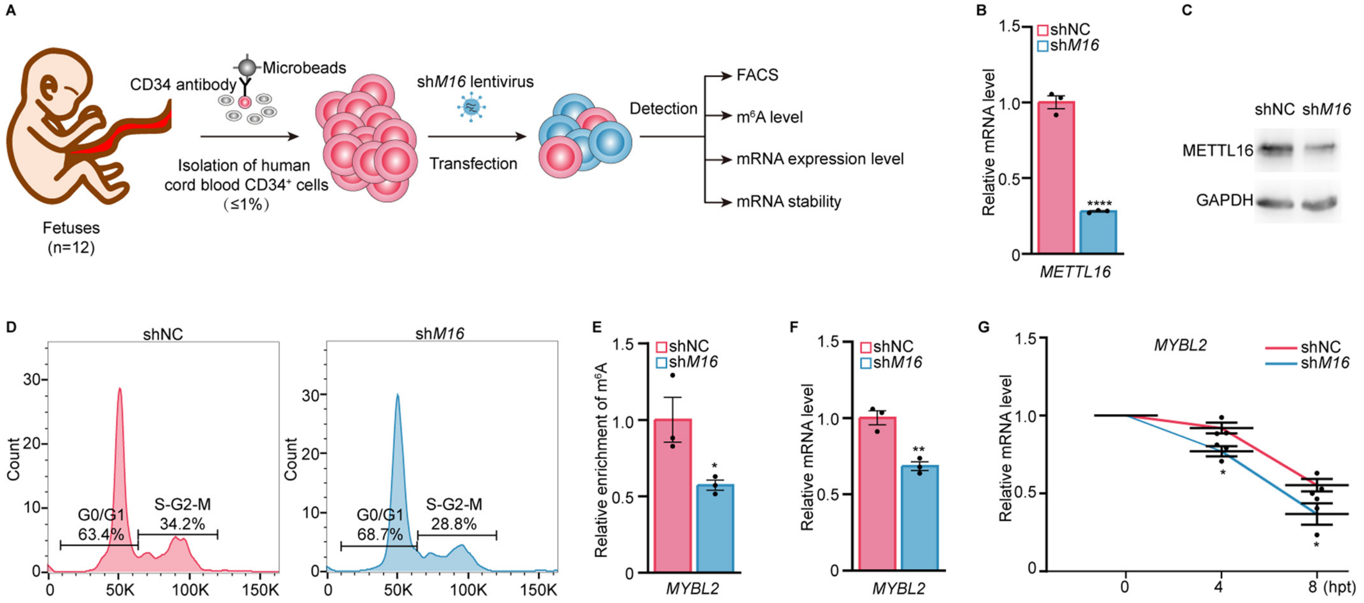

Figure EV6. The METTL16/m^{6}A/*MYBL2* axis in G1/S progression is conserved in human cord blood CD34$^+$ cells.

(**A**) Flow chart of human cord blood CD34$^+$ cell isolation and lentivirus transduction. (**B**) Validation of the knockdown (KD) efficiency of the shRNA against *METTL16* by qPCR in CD34$^+$ cord blood cells derived from healthy donors. $n = 3$ biological replicates, 2 healthy donors per group. (**C**) Validation of the KD efficiency of the shRNA against *METTL16* by western blot in CD34$^+$ cord blood cells derived from healthy donors. $n = 2$ biological replicates, 2 healthy donors per group. (**D**) Flow analysis showing increased G0/G1 phase in CD34$^+$ cord blood cells derived from healthy donors after *METTL16* knockdown. $n = 2$ biological replicates, 2 healthy donors per group. (**E**) m^{6}A enrichment in *MYBL2* mRNA in CD34$^+$ cord blood cells derived from healthy donors after *METTL16* knockdown by meRIP-qPCR. n = 3 biological replicates, 2 healthy donors per group. (**F**) qPCR analysis showing that the mRNA expression level of *MYBL2* in CD34$^+$ cord blood cells derived from healthy donors after *METTL16* knockdown. $n = 3$ biological replicates, 2 healthy donors per group. (**G**) qPCR analysis of CD34$^+$ cord blood cells derived from healthy donors treated with actinomycin D for 4 and 8 h showing accelerated *MYBL2* mRNA degradation after *METTL16* knockdown. $n = 3$ biological replicates, 2 healthy donors per group. Data information: In (**B**, **E**–**G**), data were represented as mean ± SEM, *adjusted $P < 0.05$, **adjusted $P < 0.01$, **** adjusted $P < 0.0001$, Student's *t*-test (**B**, **E**, **F**), two-way ANOVA analysis with post hoc test of Tukey's multiple comparison correction (**G**).
